# Interhomolog polymorphism shapes meiotic crossover within the Arabidopsis *RAC1* and *RPP13* disease resistance genes

**DOI:** 10.1371/journal.pgen.1007843

**Published:** 2018-12-13

**Authors:** Heïdi Serra, Kyuha Choi, Xiaohui Zhao, Alexander R. Blackwell, Juhyun Kim, Ian R. Henderson

**Affiliations:** 1 Department of Plant Sciences, University of Cambridge, Cambridge, United Kingdom; 2 Department of Life Sciences, Pohang University of Science and Technology, Pohang, Gyeongbuk, Republic of Korea; Weizmann Institute, ISRAEL

## Abstract

During meiosis, chromosomes undergo DNA double-strand breaks (DSBs), which can be repaired using a homologous chromosome to produce crossovers. Meiotic recombination frequency is variable along chromosomes and tends to concentrate in narrow hotspots. We mapped crossover hotspots located in the *Arabidopsis thaliana RAC1* and *RPP13* disease resistance genes, using varying haplotypic combinations. We observed a negative non-linear relationship between interhomolog divergence and crossover frequency within the hotspots, consistent with polymorphism locally suppressing crossover repair of DSBs. The *fancm*, *recq4a recq4b*, *figl1* and *msh2* mutants, or lines with increased *HEI10* dosage, are known to show increased crossovers throughout the genome. Surprisingly, *RAC1* crossovers were either unchanged or decreased in these genetic backgrounds, showing that chromosome location and local chromatin environment are important for regulation of crossover activity. We employed deep sequencing of crossovers to examine recombination topology within *RAC1*, in wild type, *fancm*, *recq4a recq4b* and *fancm recq4a recq4b* backgrounds. The *RAC1* recombination landscape was broadly conserved in the anti-crossover mutants and showed a negative relationship with interhomolog divergence. However, crossovers at the *RAC1* 5′-end were relatively suppressed in *recq4a recq4b* backgrounds, further indicating that local context may influence recombination outcomes. Our results demonstrate the importance of interhomolog divergence in shaping recombination within plant disease resistance genes and crossover hotspots.

## Author summary

Sexually reproducing plants and animals produce gametes with half the number of chromosomes, which can participate in fertilization. A specialized cell division called meiosis generates gametes, where the chromosomes are copied once and segregated twice. A further key feature of meiosis is that chromosomes physically pair and undergo reciprocal exchanges, called crossovers. Due to independent chromosome segregation and crossovers, meiosis creates gametes that are genetically diverse, which has a major effect on patterns of sequence variation in populations. Interestingly, the frequency of crossover is also highly variable along the lengths of chromosomes and tends to be concentrated in narrow hotspots. Here we studied two crossover hotspots in detail that are located within disease resistance genes, using the model plant Arabidopsis. We show that within these hotspots, greater levels of genetic difference between the recombining chromosomes locally inhibits crossover formation. We also show that hotspots within one of these resistance genes are surprisingly resistant to genetic backgrounds that increase crossovers elsewhere in the genome. This indicates that patterns of polymorphism and hotspot location along the chromosome are both important for control of recombination activity.

## Introduction

Meiosis is a specialized cell division that is central to sexual reproduction in eukaryotes [1,2]. It is characterized by a single round of DNA replication, followed by two successive rounds of chromosome segregation, generating four haploid gametes from a single diploid mother cell [1,2]. During prophase I, homologous chromosomes also pair and undergo reciprocal genetic exchange, termed crossover [3]. Crossovers ensure accurate chromosome segregation, by creating a physical link between homologous chromosomes that, together with chromosome cohesion, promote balanced segregation during the first meiotic division [1,2]. Importantly, meiotic crossovers also create genetic diversity by recombining linked variation [1,2,4]. Meiotic recombination thus impacts upon genetic adaptation in sexual populations, by combining independently arising mutations more rapidly than in asexual species [4].

Meiotic recombination initiates via DNA double-strand breaks (DSBs) generated by SPO11 topoisomerase VI-related transesterases [5–7]. In Arabidopsis ~100–200 meiotic DSBs form per meiosis, estimated from immunostained RAD51, DMC1, RPA1 and _Ɣ_H2A.X foci that occur along paired chromosomes at leptotene stage [8–10]. In budding yeast, endonuclease and exonuclease activities (Mre11-Rad50-Xrs2, Sae2 and Exo1) act at DSB sites to generate 3′-overhanging single-strand DNA (ssDNA) [11–14], between 100s and 1000s of nucleotides in length [15,16]. Resected ssDNA is bound first by RPA1 and then RAD51 and DMC1 proteins, which together promote interhomolog invasion and formation of a displacement loop (D-loop) [17,18]. Stabilization of the D-loop likely involves template-driven DNA synthesis from the invading 3′-end [3,19]. Strand invasion intermediates may then undergo second-end capture to form double Holliday junctions (dHJs), which can be resolved as a crossover or non-crossover, or dissolved [20,21].

The conserved ZMM pathway acts to promote meiotic DSB repair via dHJs and crossovers [2,3,22]. In Arabidopsis ~10 DSBs per meiosis are repaired as crossovers [23–26]. The majority (~90%) of these crossovers are dependent on the ZMM pathway in Arabidopsis [2]. This pathway includes ZIP4, the SHOC1 XPF endonuclease and its interacting partner PTD, the MER3 DNA helicase, the HEI10 E3 ligase, the MSH4/MSH5 MutS-related heterodimer and the MLH1/MLH3 MutL-related heterodimer [2,22]. ZMM factors are thought to stabilise interhomolog joint molecules, including dHJs, and promote crossover resolution [27]. ZMM-dependent crossovers (also known as Class I) also show the phenomenon of interference, meaning that they are more widely distributed than expected at random [2,22,28,29].

In plants and other eukaryotes a large excess of initiating meiotic DSBs proceed to resection and strand invasion, but are repaired as non-crossovers (that may be detectable as gene conversions), or via inter-sister repair [2]. Disassociation of strand invasion events occurs via partially redundant anti-crossover pathways in Arabidopsis that include, (i) the FANCM helicase and its cofactors MHF1 and MHF2 [30–32], (ii) the BTR complex: RECQ4A, RECQ4B, TOPOISOMERASE3a and RECQ4-MEDIATED INSTABILITY1 (RMI1) [33–37], and (iii) FIDGETIN-LIKE1 (FIGL1) and FLIP1 [38,39]. Plants mutated in these anti-crossover pathways show increased non-interfering crossovers, which are also known as Class II events [2]. This likely occurs as a consequence of reduced disassociation of interhomolog strand invasion events, which are alternatively repaired by non-interfering crossover pathway(s) [30,34,38], including via MUS81 [40,41]. Hence, alternative repair pathways act on SPO11-dependent DSBs during meiosis to balance crossover and non-crossover outcomes.

Due to the formation of interhomolog joint molecules during meiotic recombination, sequence polymorphisms between chromosomes can result in mismatched base pairs [42]. During the mitotic cell cycle DNA mismatches, or short insertion-deletions (indels), caused by base mis-incorporation during replication, or exogenous DNA damage, can be detected by MutS-related heterodimers [43]. MutS recognition of mismatches and the subsequent promotion of repair plays a major anti-mutagenic role *in vivo* [43]. MutS complexes also play anti-crossover roles during meiosis when heterozygosity leads to sequence mis-matches, following interhomolog strand invasion [44–47]. Accumulating evidence also indicates that Class I and II crossover repair pathways show differential sensitivity to levels of interhomolog polymorphism. For example, Arabidopsis *fancm* mutations show increased crossovers in inbred, but not in hybrid contexts, whereas *figl1* and *recq4a recq4b* mutations are effective at increasing crossovers in both situations [34,38,48–51]. This implies that the non-interfering crossover repair pathways acting in these backgrounds are influenced differently by interhomolog polymorphism. Genome-wide mapping of crossovers in anti-crossover mutants, or backgrounds with additional copies of the ZMM gene *HEI10*, have further shown that the resulting recombination increases are highly distalized towards the sub-telomeres, correlating with regions of lowest interhomolog polymorphism [49–51]. At larger physical scales (e.g. kb to Mb) structural rearrangements, such as translocations and inversions, are potently associated with crossover suppression [52,53], and increased levels of divergence within the Arabidopsis *14a* hotspot correlated with reduced crossover frequency [54].

Despite the suppressive effects of interhomolog polymorphism on recombination, at the chromosome scale wild type crossovers in Arabidopsis show a weak positive relationship with interhomolog diversity, i.e. heterozygosity [49,50]. Linkage disequilibrium (LD) based historical crossover estimates are also positively correlated with population diversity in Arabidopsis [48,55,56]. Furthermore, juxtaposition of megabase scale heterozygous and homozygous regions in Arabidopsis associates with increased crossover frequency in the heterozygous regions, which is dependent on the Class I repair pathway [48]. Therefore, the relationship between interhomolog polymorphism and meiotic crossover frequency is complex, with both negative and positive relationships, depending on the scale and region analysed.

In this work we explore the influence of interhomolog polymorphism on meiotic recombination at the scale of crossover hotspots in *Arabidopsis thaliana*. Specifically, we map crossovers across the *RAC1* and *RPP13* disease resistance genes, which encode proteins that recognise effector proteins from the oomycete pathogens *Albugo laibachii* and *Hylaoperonospora parasitica*, respectively [57,58]. We observe a non-linear negative relationship between interhomolog polymorphism and crossover frequency within both *RAC1* and *RPP13*, supporting a local inhibitory effect of mismatches on crossover formation. This relationship was observed using different *RAC1* haplotypic combinations, which vary in the density and pattern of polymorphism. Despite recombination rates increasing genome-wide in anti-crossover mutants and *HEI10* transgenic lines, *RAC1* crossover frequency was stable or significantly decreased in these backgrounds. The resistance of *RAC1* to genome-wide crossover increases may relate to the high level of interhomolog polymorphism at this locus, the pericentromeric location or local chromatin environment. Using deep sequencing of *RAC1* crossover molecules we show that the negative relationship between crossovers and interhomolog divergence is maintained in the *fancm*, *recq4a recq4b* and *fancm recq4a recq4b* anti-crossover mutants. However, crossover frequency at the 5′ end of *RAC1* was relatively decreased in *recq4a recq4b* mutant backgrounds, indicating an influence of local context on recombination outcomes.

## Results

### Meiotic recombination and chromatin at the *RAC1* and *RPP13* disease resistance genes

**Fig 1:**
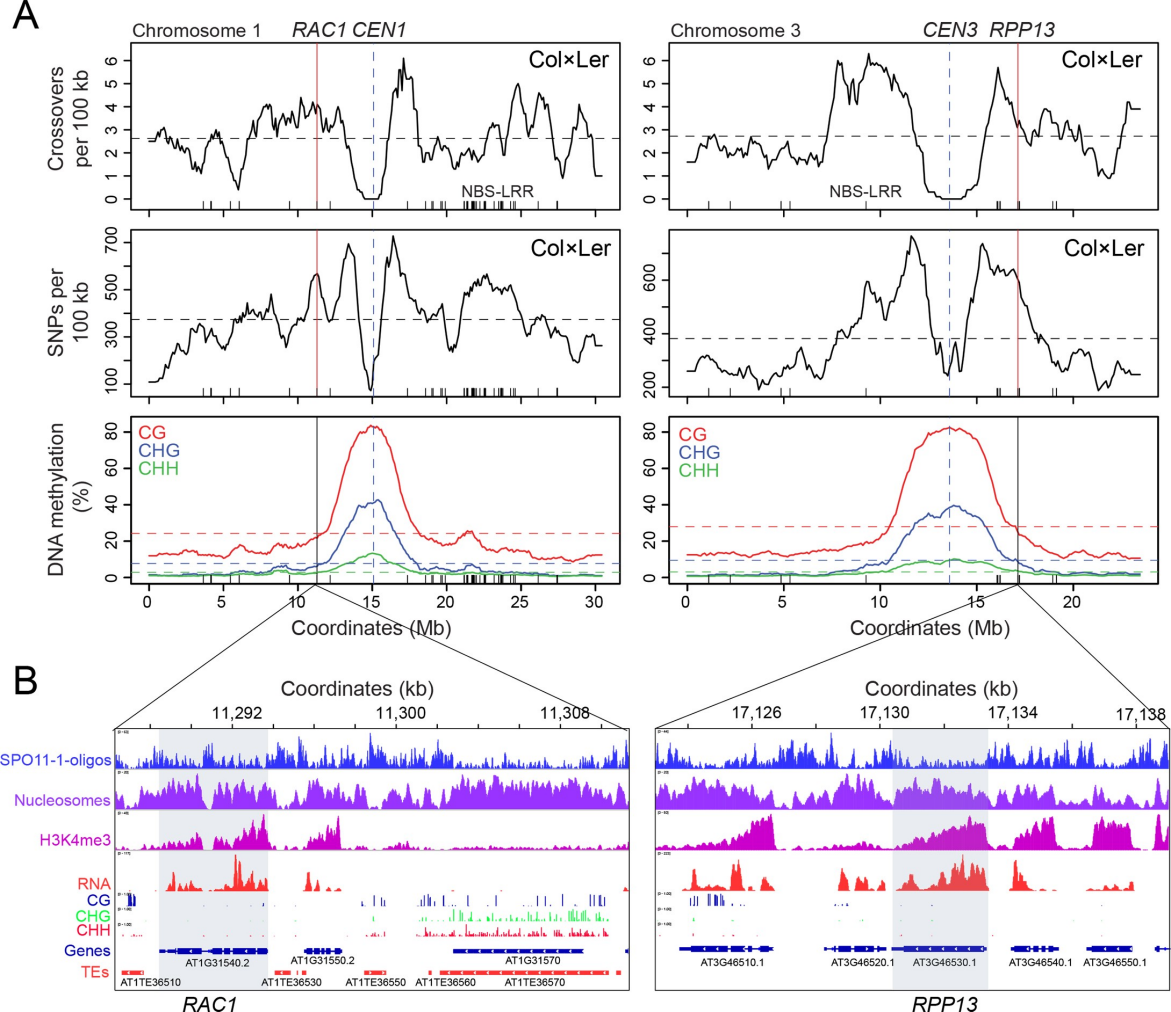
Chromatin and recombination landscapes around the *RAC1* and *RPP13* NBS-LRR disease resistance genes. **(A)** Crossover frequency (crossovers/100 kb mapped by genotyping-by-sequencing of Col×Ler F_2_) [50,60], interhomolog divergence (Col/Ler SNPs/100 kb) [63], and % DNA methylation (CG red, CHG blue, CHH green) [64], along chromosomes 1 and 3. Vertical dotted lines indicate the centromeres. Mean values are indicated by horizontal dotted lines. NBS-LRR gene positions are indicated by ticks on the x-axis. The position of *RAC1* and *RPP13* are indicated by the solid vertical lines. **(B)** Histograms for the *RAC1* and *RPP13* regions showing library size normalized coverage values for SPO11-1-oligonucleotides (blue), nucleosome occupancy (purple, MNase-seq), H3K4me3 (pink, ChIP-seq), RNA-seq (red) and % DNA methylation (BSseq) in CG (blue), CHG (green) and CHH (red) sequence contexts [64,66]. Gene (blue) and transposon (red) annotations are highlighted, and the positions of *RAC1* and *RPP13* are indicated by grey shading.

We previously identified the *RESISTANCE TO ALBUGO CANDIDA1* (*RAC1*) Arabidopsis disease resistance gene region as containing crossover hotspots, using both historical linkage disequilibrium (LD) based estimates and experimental pollen-typing in Col×Ler F_1_ hybrids [59,60]. *RAC1* encodes a TIR-NBS-LRR domain resistance protein, which recognises effectors from the oomycete pathogens *Albugo candida* and *Hylaoperonospora parasitica* [57,61,62]. *RAC1* exists as a singleton TIR-NBS-LRR gene in most accessions and shows high levels of population genetic diversity (e.g. θ = 0.012–0.013; π = 0.043–0.054) [55,56,59,60]. We compared the *RAC1* locus to a recombination map of 3,320 crossovers mapped by genotyping-by-sequencing (GBS) of 437 Col×Ler F_2_ individuals (mean crossover resolution = 970 bp) (Fig 1A) [50,60]. We also assessed levels of interhomolog polymorphism by measuring the density of Col/Ler SNPs per 100 kb [63], in addition to levels of DNA methylation as an indication of heterochromatin (Fig 1A) [64]. Together this showed that *RAC1* is located on the edge of pericentromeric heterochromatin, in a region of higher than average crossover frequency and interhomolog polymorphism (Fig 1A).

Using historical recombination maps generated by analysing the 1,001 Genomes Project SNP data, we identified *RPP13* as a further NBS-LRR gene with higher than average historical crossover frequency (10.56–10.57 cM/Mb), and high levels of population SNP diversity (θ = 0.011–0.013, π = 0.044–0.045) [55,56,59,60]. These levels of diversity and recombination were comparable to those observed at *RAC1*. RPP13 recognizes the *Hylaoperonospora parasitica* effector ATR13 to mediate disease resistance, and which together display co-evolutionary dynamics [58,65]. Similar to *RAC1*, *RPP13* is a singleton NBS-LRR gene located on the edge of pericentromeric heterochromatin, in a region of higher than average crossover frequency and interhomolog polymorphism (Fig 1A).

We examined the *RAC1* and *RPP13* regions using genome-wide maps of chromatin and meiotic recombination [60,64,66]. Nucleosome occupancy was assessed using MNase-seq data, which showed enrichment within the gene exons and was depleted within the promoter, intron and terminator regions (Fig 1B). Arabidopsis SPO11-1-oligonucleotides mark meiotic DSB sites and show an inverse pattern to nucleosome occupancy [66]. Consistently, at *RAC1* and *RPP13* we observed higher levels of SPO11-1-oligonucleotides in the nucleosome-depleted intergenic regions (Fig 1B). H3K4me3 ChIP-seq showed enrichment at the 5′-end of the genes, consistent with active transcription [67], and we observed *RAC1* and *RPP13* transcription using RNA-seq data from stage 9 flowers (Fig 1B) [60,66]. Both *RAC1* and *RPP13* show low levels of DNA methylation, in contrast to the *ATENSPM3* EnSpm/CACTA (AT1TE36570) element located adjacent to *RAC1*, which is heavily methylated, nucleosome-dense and suppressed for SPO11-1-oligos (Fig 1B). The *RAC1* promoter intergenic region contains short fragments of several transposable elements, including *HELITRONY3* and *ATREP15* Helitrons (Fig 1B). Transposable elements in these families have relatively low DNA methylation, are nucleosome-depleted and show higher levels of SPO11-1-oligos, compared with other repeat families in Arabidopsis (Fig 1B) [66]. Therefore, despite the location of *RAC1* and *RPP13* on the edge of pericentromeric heterochromatin, these genes display euchromatic chromatin and recombination features (Fig 1A and 1B) [64,66].

### Crossover hotspots within the *RAC1* and *RPP13* disease resistance genes

**Fig 2:**
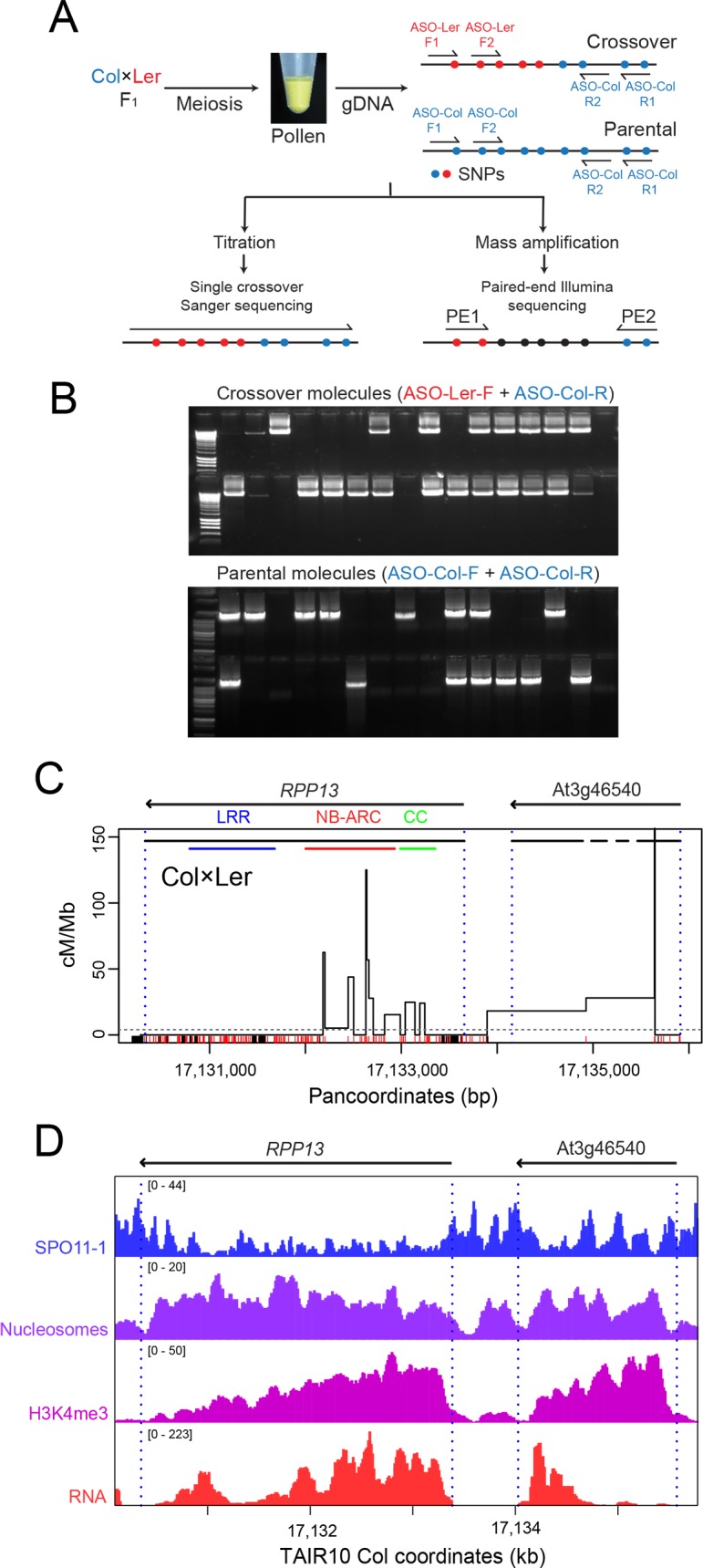
Crossover hotspots within the *RPP13* disease resistance gene. **(A)** Schematic of the pollen-typing method using allele-specific PCR to quantify and sequence crossover molecules. **(B)** Inset are representative ethidium bromide stained gels showing crossover and parental molecule *RAC1* PCR amplification products from diluted pollen F_1_ Col/Ler genomic DNA. **(C)** Crossover frequency (cM/Mb) within the region of the disease resistance gene *RPP13* measured using titration and sequencing of individual crossover molecules from Col×Ler pollen F_1_ genomic DNA. Gene TSS and TTS are indicated by vertical dotted blue lines. Horizontal lines indicate exon (black) positions, in addition to protein domains (coiled coil (green), NB-ARC (red) and LRR (blue)) for RPP13. Col/Ler SNPs (red) and indels (black) are indicated on the x-axis. Data is plotted against the Col/Ler panmolecule, which includes all insertions and deletions. The horizontal dotted line indicates the genome-average recombination rate for male Col×Ler crosses [74]. **(D)** Histograms for the *RPP13* region showing library size normalized values for SPO11-1-oligonucleotides (blue), nucleosome occupancy (purple, MNase-seq), H3K4me3 (pink, ChIP-seq) and RNA-seq (red) [60,66].

In order to experimentally measure crossovers within *RAC1* and *RPP13* we used pollen-typing [68,69]. This method uses allele-specific PCR amplification from F_1_ hybrid genomic DNA extracted from gametes, in order to quantify and sequence crossover molecules (Fig 2A and S1 Fig) [68,69]. This method is directly analogous to mammalian sperm-typing methods [70–73]. Genomic DNA is extracted from pollen (male gametophytes) collected from individuals that are heterozygous over a known crossover hotspot (Fig 2A). Allele-specific primers annealing to polymorphic sites flanking the region of interest are used to PCR amplify single crossover or parental molecules, using diluted DNA samples (Fig 2A). Titration is used to estimate the concentrations of amplifiable crossover and parental molecules, which are used to calculate genetic distance (cM = (crossovers/(crossovers+parentals))×100) (Fig 2A). Sanger sequencing of PCR products amplified from single crossover molecules is performed to identify internal recombination points, to the resolution of individual polymorphisms (Fig 2A). Together this information describes the recombination rate (cM/Mb) topology throughout the PCR amplified region [68,69]. It is also possible to mass amplify crossover molecules, which may be pooled and sequenced using paired-end reads to identify crossover locations (Fig 2A) [68].

To investigate whether *RPP13* was associated with crossover hotspots we designed and optimised Col/Ler allele-specific oligonucleotides (ASOs) flanking this resistance gene (S1A Fig). The *RPP13* ASO primers specifically amplified crossovers from pollen, and not leaf DNA, extracted from the same Col/Ler F_1_ plants (S1B Fig). We performed DNA titrations to quantify crossover and parental molecules across *RPP13* and observed a genetic distance of 0.055 cM, equivalent to 9.78 cM/Mb across the 5,626 bp amplicon (S1 Table). When analysing crossovers we plot their frequency against panmolecules, where we include all bases from both accessions (S2 Fig and S2–S5 Tables). For example, the *RPP13* amplicon is 5,431 bp in Col, 5,526 bp in Ler and 5,626 bp in the Col/Ler panmolecule, with 195 inserted bases from Ler and 100 from Col (S2 Fig and S5 Table). We sequenced 44 single crossover molecules and observed clustering of recombination events at the 5′-end of *RPP13*, overlapping regions encoding the coiled-coil and NB-ARC domains (Fig 2C). *RPP13* shows a peak crossover rate of 125 cM/Mb (S6 Table), compared to the genome average of 4.82 cM/Mb for male Col/Ler F_1_ hybrids [74]. Crossovers were also observed in the adjacent gene At3g46540 (Fig 2C). A single crossover was observed in a 5 bp interval within At3g46540, which results in a high recombination estimate (250 cM/Mb) (S6 Table). However, as a single crossover event is responsible for this recombination measurement, this may reflect sampling, rather than the presence of a high activity hotspot. The region of highest crossover activity within *RPP13* overlaps with nucleosome-occupied, H3K4me3-modified exon sequences (Fig 2D). In contrast, highest SPO11-1-oligos occur in flanking nucleosome-depleted intergenic regions (Fig 2D).

**Fig 3:**
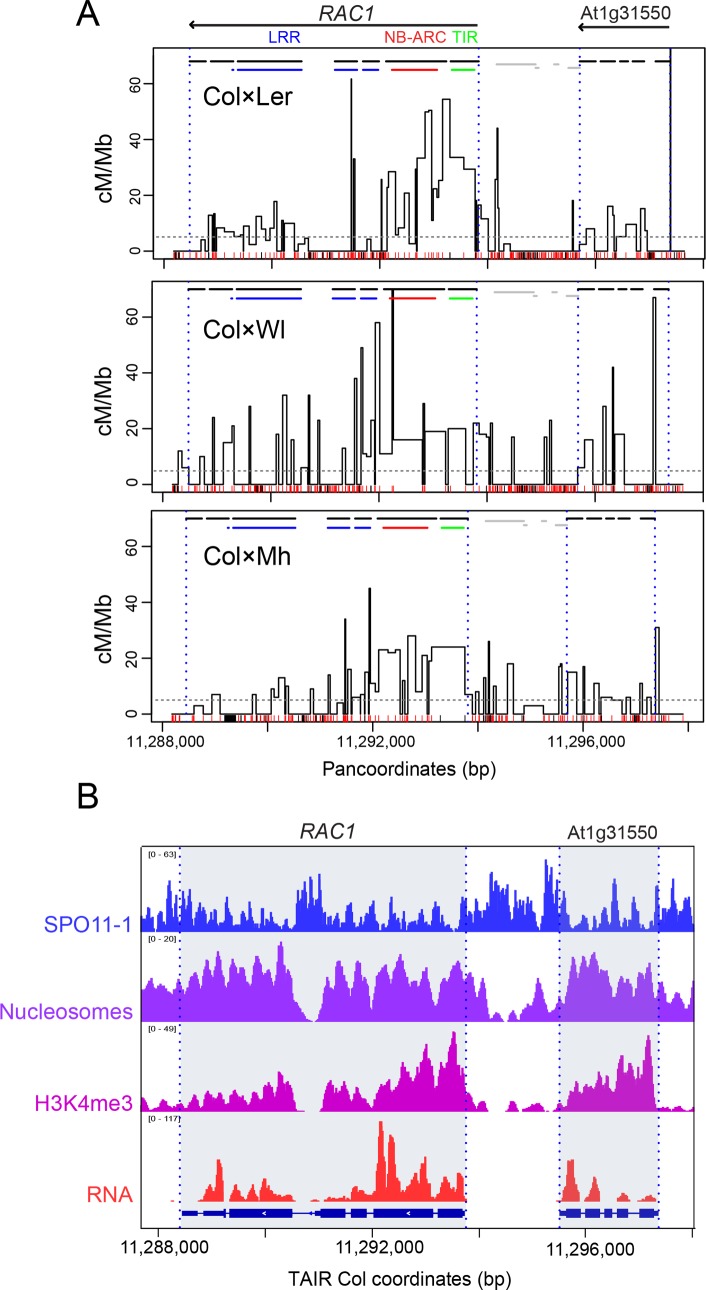
*RAC1* crossover hotspots in Col×Ler, Col×Wl and Col×Mh hybrids. **(A)** Crossover frequency (cM/Mb) within the region of the *RAC1* disease resistance gene measured using titration and sequencing of single crossover molecules from Col×Ler (0.074 cM), Col×Wl (0.074 cM) and Col×Mh (0.064 cM) pollen F_1_ genomic DNA. Recombination is plotted against the panmolecules, which include all bases from both parental accessions. Gene TSS/TTS are indicated by vertical dotted lines and exons by horizontal black lines. The position of RAC1 TIR (green), NB-ARC (red) and LRR (blue) domain-encoding sequences are indicated by the colored horizontal lines. SNPs (red) and indels (black) are indicated by the ticks on the x-axis. The horizontal dotted line indicates the genome-average recombination rate from male Col×Ler crosses [74]. **(B)** Histograms for the *RAC1* region showing library size normalized values for SPO11-1-oligonucleotides (blue), nucleosome occupancy (purple, MNase-seq), H3K4me3 (pink, ChIP-seq) and RNA-seq (red) [60,64,66]. The positions of *RAC1* and *GDSL* (At1g31550) are indicated by grey shading.

The *RAC1* gene is located within a 9,482 bp (Col/Ler) pollen-typing PCR amplicon (Fig 3). We previously reported analysis of 181 single crossover molecules within the *RAC1* amplicon [60], which we combined with an additional 59 events here to give a total of 240 crossovers (S7 and S8 Tables). We observed a peak recombination rate of 61.7 cM/Mb within *RAC1* (Fig 3A and S8 Table). An adjacent gene contained within the amplicon, At1g31550 (*GDSL*), also showed intragenic crossovers (Fig 3A) [60]. Similar to *RPP13*, elevated crossover frequency within *RAC1* overlapped nucleosome-occupied and H3K4me3-enriched exon sequences (Fig 3B) [66]. Highest crossover frequency occurred within the *RAC1* 5′ exons encoding the NB-ARC and TIR domains (Fig 3A). A further similarity with *RPP13*, is that highest levels of SPO11-1-oligos are observed in the nucleosome-depleted intergenic regions flanking *RAC1*, in addition to the largest intron (Figs 2 and 3). Hence, both *RPP13* and *RAC1* have highest crossover frequency within transcribed gene 5′ regions, despite higher levels of initiating SPO11-1 dependent DSBs occurring in the adjacent intergenic regions.

### Interhomolog divergence suppresses crossovers within *RAC1* and *RPP13*

*RAC1* and *RPP13* show high levels of interhomolog polymorphism between Col and Ler, with 27.4 and 34.5 SNPs/kb, respectively (compared to the genome average of 3.85 SNPs/kb) [63]. This is also reflected in high levels of population genetic diversity at *RAC1* and *RPP13* [55,56,59,60]. Therefore, we repeated *RAC1* pollen-typing with crosses using different parental accessions, where the pattern of interhomolog divergence varied, in order to investigate its influence on crossover frequency (Fig 3A). Pollen-typing relies on allele-specific primers that anneal at SNPs or indels [68,75]. Therefore, we used the 1,001 Genomes Project data to identify accessions sharing the Col/Ler allele-specific primer polymorphisms, but differing with respect to internal polymorphisms within the *RAC1* amplicon (Fig 3A and S2 Fig). This identified Mh-0 (Mühlen, Poland) and Wl (Wildbad, Germany) as meeting these criteria. Col×Wl and Col×Mh have 33.0 and 21.1 SNPs/kb within the *RAC1* amplicon, respectively. Therefore, we extracted pollen genomic DNA from Col×Wl and Col×Mh F_1_ hybrids and amplified and sequenced 92 and 124 crossover molecules, respectively (Fig 3A and S9 and S10 Tables). For Col×Ler and Col×Mh we performed DNA titration experiments and did not observe a significant difference in crossover frequency (*P =* 0.309) (S7 Table).

**Fig 4:**
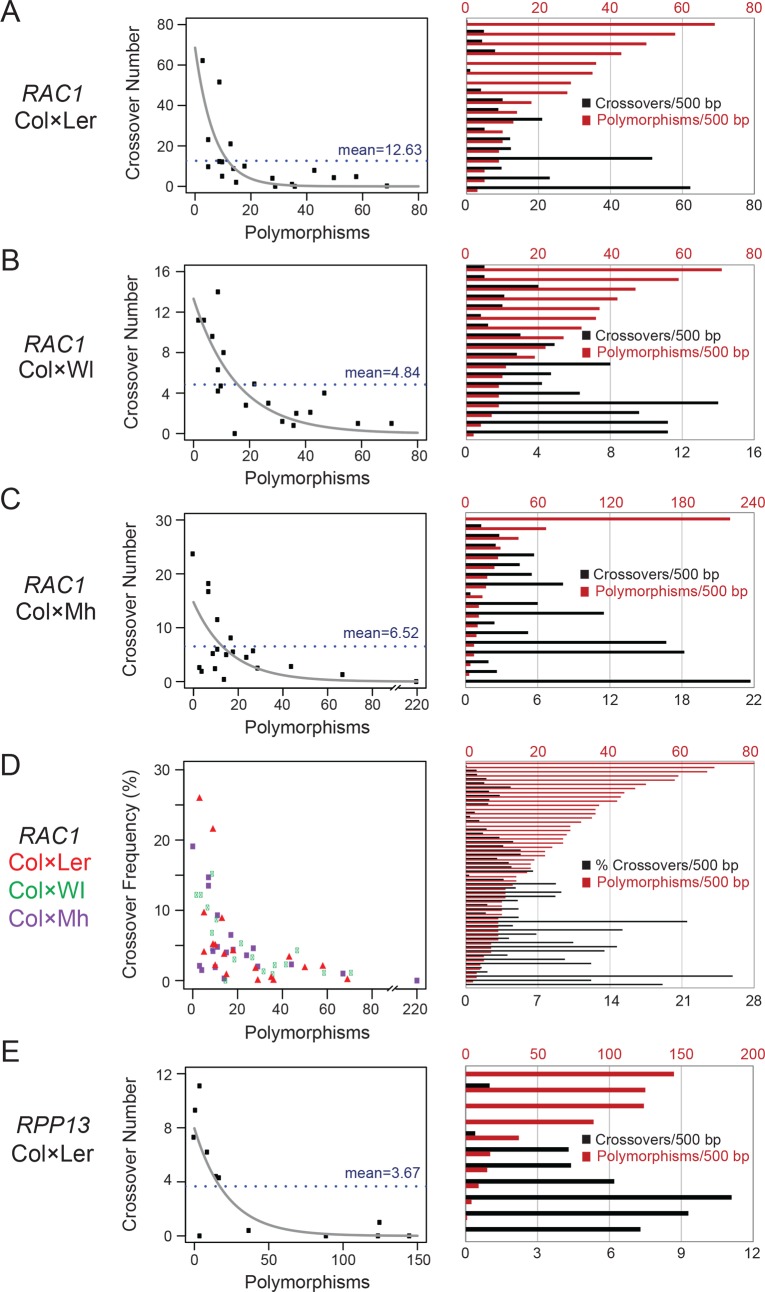
Interhomolog divergence suppresses crossovers within *RAC1* and *RPP13*. **(A)** Col×Ler crossovers and polymorphisms were calculated in adjacent 500 bp windows throughout the *RAC1* region, where SNPs are counted as one and indels by their length, using panmolecule coordinates. The blue horizontal dotted lines indicate the value of crossovers per window if they were evenly distributed. The grey line represents a non-linear model fitted to the data using the formula; y = log(a)+b×x^(-c), where y is the number of crossovers, x is polymorphisms, a is the intercept and b and c are scale parameters. On the right the same data are plotted as histograms of crossover (black) and polymorphisms (red) per 500 bp window. **(B)** As for (A), but analysing Col×Wl. **(C)** As for (A), but analysing Col×Mh. **(D)** As for (A), but analysing all windows from Col×Ler (red), Col×Wl (green) and Col×Mh (purple). Due to total crossovers analysed varying between hybrids, crossovers were first calculated as a % for each window. **(E)** As for (A), but analysing the *RPP13* amplicon from a Col×Ler hybrid.

Crossover topology within the *RAC1* amplicon was conserved between the three haplotype combinations tested (Fig 3A and S8–S10 Tables). For instance, by comparing crossovers in adjacent 500 bp windows (counted against the Col reference sequence) we observed significant positive correlations between the recombination maps (Spearman’s Col×Ler vs Col×Wl *r* = 0.595 *P =* 9.14×10^−3^; Col×Ler vs Col×Mh *r* = 0.612 *P =* 6.91×10^−3^; Col×Wl vs Col×Mh *r* = 0.723 *P =* 6.96×10^−4^). For each cross, highest crossover frequency was observed within the *RAC1* and *GDSL* transcribed regions (Fig 3A and S8–S10 Tables). In each case, we calculated the number of crossovers and polymorphisms in adjacent 500 bp windows (Fig 4 and S11 Table), where SNPs were counted as one and indels were counted according to their length in base pairs. In all cases, a significant negative relationship was observed between crossovers and polymorphisms (all *RAC1* windows, Spearman’s *r* = -0.685 *P* = 1.11×10^−8^) (Fig 4 and S11 Table). This was also observed when analysing the *RPP13* Col×Ler data in the same manner (Spearman’s *r* = -0.890, *P* = 2.43×10^−4^) (Fig 4E and S12 Table). We fitted a non-linear model to the data using the formula y = log(a)+b×x^(-c), where y is the number of crossovers, x is polymorphisms, a is the intercept and b and c are scale parameters. Together this shows a strong, negative non-linear relationship between interhomolog polymorphisms and crossover frequency within *RAC1* and *RPP13*. We previously found that at the *RAC1* 5′-end there is a strong CTT motif, which have been associated with high crossover frequency in Arabidopsis [23,59,60,76]. Ler and Wl share a SNP in this motif but this does not obviously associate with differences in recombination rate [Col/Mh: CTTCGTCATCTTCTTCT; Ler/Wl: CTTCTTCATCTTCTTCT].

### *RAC1* crossover frequency is resistant to changes in meiotic recombination pathways

**Fig 5:**
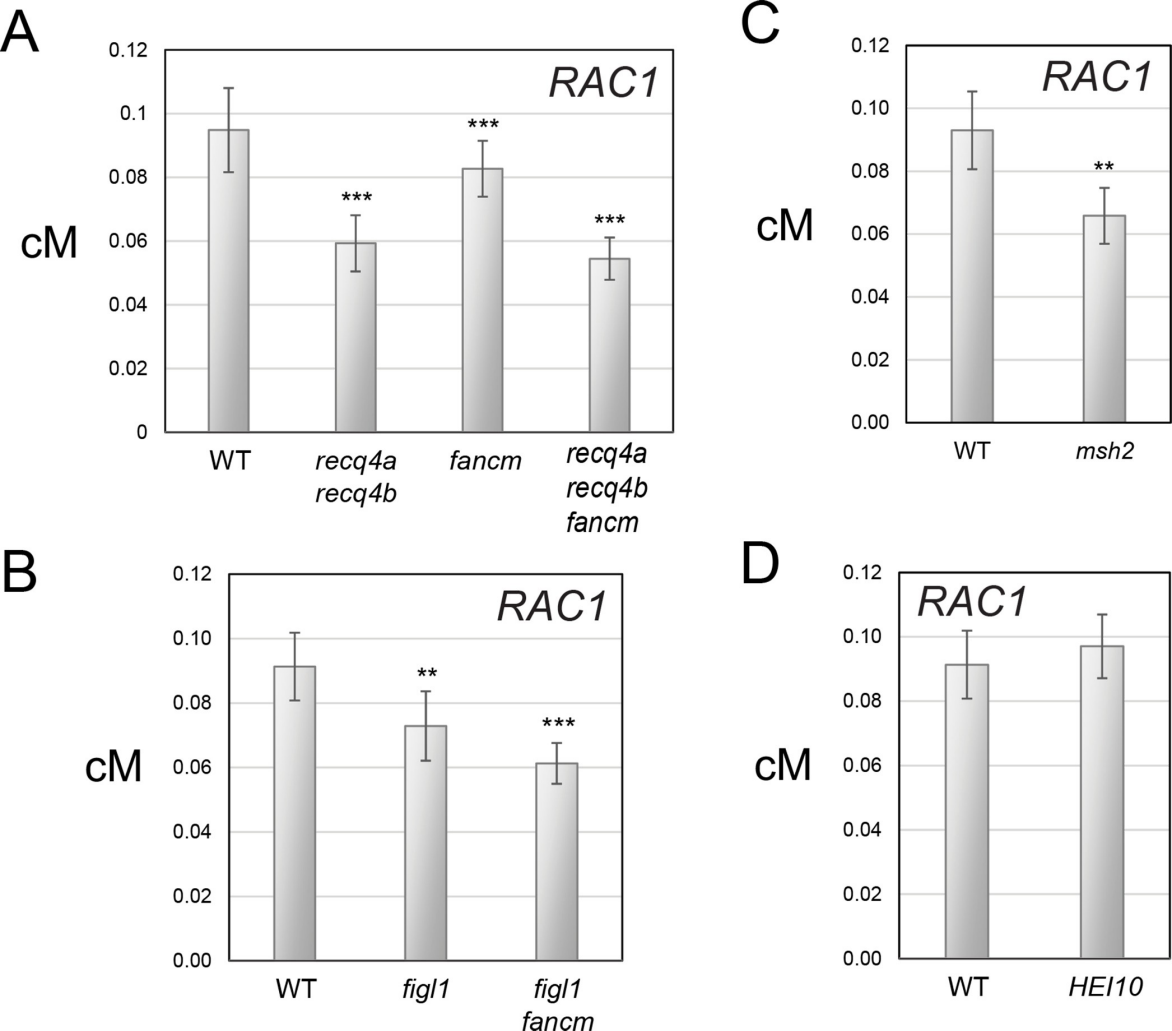
*RAC1* genetic distance in backgrounds with altered meiotic recombination pathways. **(A)** Barplots showing *RAC1* genetic distance (cM) measured in wild type, *recq4a recq4b*, *fancm* and *recq4a recq4b fancm* using single crossover and parental molecule titration. Error bars represent measurement standard deviation (square root of the variance). To test for differences the mean number of crossovers and parental molecules per μl were used to construct 2×2 contingency tables and Chi-square tests performed. The significance indicators ** and *** report a *P-*value of between 0.01–0.0001 and <0.0001, respectively. **(B)**. As for (A), but showing data for wild type, *figl1* and *figl1 fancm*. **(C)** As for (A), but showing data for wild type and *msh2*. **(D)** As for (A), but showing data for wild type and *HEI10*.

Previous work has revealed an influence of interhomolog polymorphism on meiotic recombination pathways in Arabidopsis [38,46,48–50]. Therefore, we sought to investigate *RAC1* crossover frequency in backgrounds with altered recombination pathways. Specifically we tested, (i) mutations in the anti-crossover genes *recq4a recq4b*, *fancm* and *figl1* [30,33,34,38,49], (ii) mutations in the *msh2* MutS homolog [46], and (iii) transgenic lines with additional *HEI10* copies [51]. Each of these genotypes was available in Col and Ler backgrounds, which could be crossed together to generate Col/Ler F_1_ hybrids used for *RAC1* pollen-typing. We measured *RAC1* crossover frequency via DNA titration experiments (Fig 5 and S13–S16 Tables). The mean number of crossovers and parental molecules per μl were used to test for significant differences, by constructing 2×2 contingency tables and performing Chi-square tests. We compared three biological replicates of wild type Col/Ler F_1_ hybrids using this method, which did not show significant differences (Fig 5A–5C and S13–S16 Tables). Previous findings have demonstrated genome-wide crossover increases in hybrid *recq4a recq4b* and *figl1* mutants [34,49,50], whereas *fancm* increases strongly in inbred, but not in hybrid backgrounds [38,48]. Despite the crossover increases in these backgrounds, we observed that *RAC1* genetic distance significantly decreased in the *recq4a recq4b*, *fancm*, *figl1*, *msh2*, *recq4a recq4b fancm* and *figl1 fancm* mutants (Fig 5A–5C and S13–S16 Tables). Furthermore, when we compared wild type with lines containing additional *HEI10* copies we did not observe a significant difference in *RAC1* crossover frequency (Fig 5D and S13–S16 Tables). Therefore, in backgrounds with either increased Class I (*HEI10*) or Class II (*fancm*, *figl1*, *recq4a recq4b*) crossover repair, the *RAC1* hotspot is unexpectedly resistant to increasing recombination frequency.

### *RAC1* crossover topology in *fancm* and *recq4a recq4b* anti-crossover mutants

To analyse *RAC1* crossover distributions in wild type versus *fancm*, *recq4a recq4b* and *recq4a recq4b fancm* anti-crossover mutants, we mass amplified crossovers and performed pollen-sequencing [60,68]. In this approach, allele-specific PCR amplification is performed using multiple independent reactions seeded with an estimated ~1–3 crossover molecules per reaction (Fig 2A). Crossover concentrations are first estimated using titration experiments (Fig 5 and S13 Table). Mass amplified allele-specific PCR products are then pooled, sonicated and used for sequencing library construction (S3 Fig) [60,68]. These libraries were subjected to paired-end 2×75 bp read sequencing (S17 Table).

**Fig 6:**
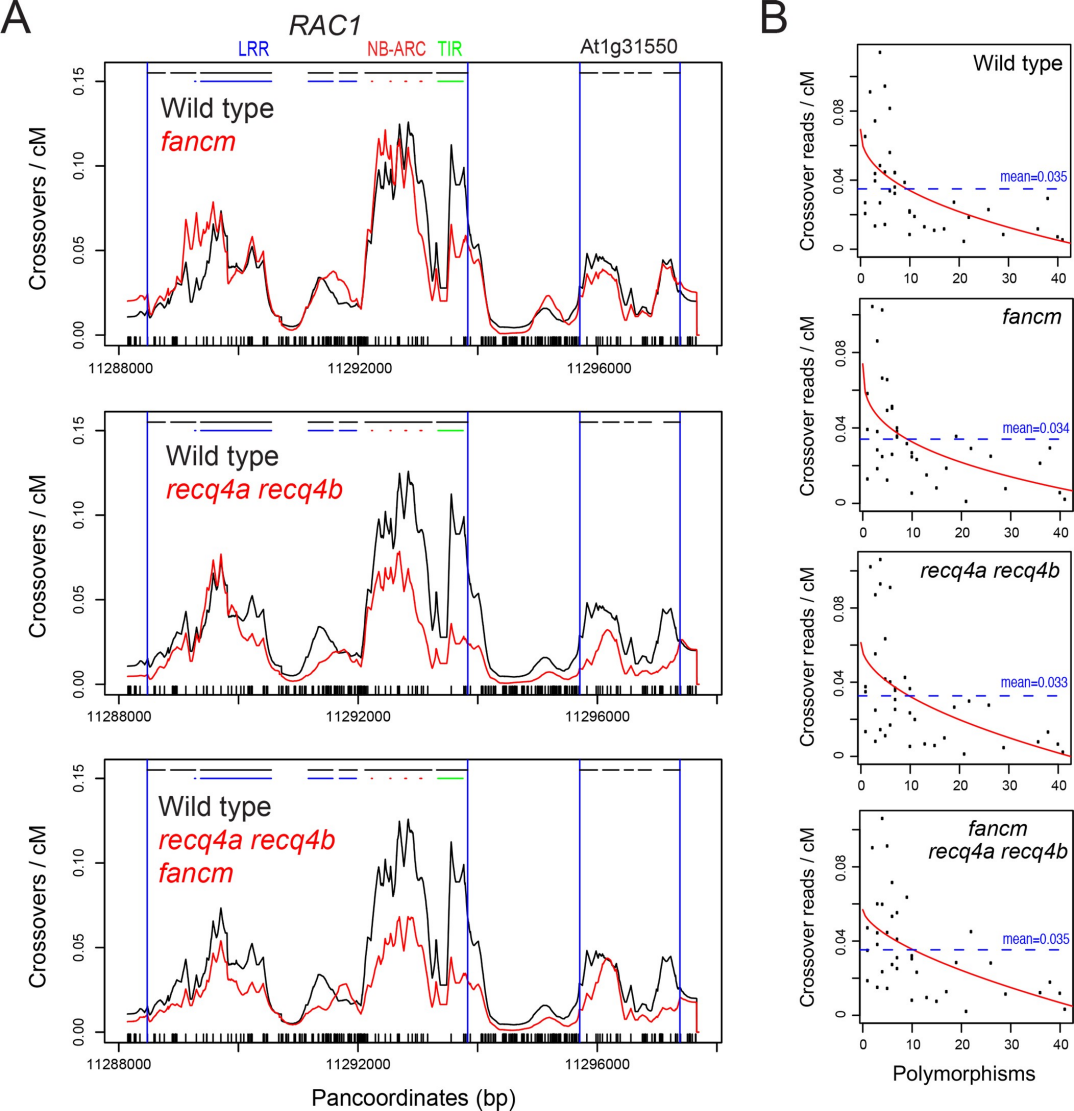
Crossover frequency across *RAC1* in wild type and *fancm*, *recq4a recq4b* and *fancm recq4a recq4b* mutants. **(A)** The coverage of crossover reads aligned against the Col×Ler panmolecule was calculated and normalized by the total number of reads analysed, and also normalized by *RAC1* genetic distance (cM) measured previously by titration. Col×Ler polymorphisms are indicated by black ticks on the x-axis. Gene TSS and TTS are indicated by vertical blue lines, and exons by horizontal black lines. In each plot wild type (black) is plotted alongside mutant backgrounds (red), which are either *fancm*, *recq4a recq4b* or *recq4a recq4b fancm*. **(B)** 250 base pair adjacent windows were used to calculate the values of crossover reads/cM and polymorphisms (SNPs = one, indels = length) and plotted. The fitted line (red) was generated using the the non-linear model y = log(a) + b×x^(-c). y is reads/cM, x is polymorphisms, a is the intercept and b and c are scale parameters. The dotted horizontal line indicates the mean level of crossover reads/cM within the analysed region.

The Col and Ler *RAC1* haplotypes from our laboratory strains were Sanger sequenced, in order to generate templates for aligning sequence data to. Read pairs were split and aligned to Col and Ler haplotypes separately using Bowtie allowing no mismatches (-v 0), such that BAM files were obtained for the reads aligned to either Col or Ler [77]. We then filtered for read pairs where one member mapped distally to Col and the other member mapped proximally to Ler, on opposite strands. This mapping configuration was expected due to the allele-specific primer orientation used during pollen-typing amplification. Consistent with these read pairs representing crossover molecules, their width distributions are similar to that of the sonicated PCR amplification products, prior to adapter ligation (S3 Fig). The crossover reads were then matched to the Col/Ler panmolecule, and counts were added to intervening sequences. These values were then normalized by the total number of crossover read pairs per library. Finally, the profiles were weighted by *RAC1* genetic distance (cM), measured previously via DNA titration (S13 Table). For wild type, *fancm*, *recq4a recq4b* and *fancm recq4a recq4b* we generated two biologically independent libraries for each genotype, sampling either ~300 or ~1,000 crossovers and the recombination profiles were found to be similar (Figs 6A and S4). Therefore, for subsequent analysis the reads from the 300 and 1,000 crossover libraries were pooled for each genotype. The wild type 1,000 crossover dataset was previously reported [60].

Overall recombination topology was similar between wild type, *fancm*, *recq4a recq4b*, and *recq4a recq4b fancm* mutants (Spearman’s wild type vs *fancm r* = 0.923 <2.2×10^−16^; wild type vs *recq4a recq4b r* = 0.902 <2.2×10^−16^; *recq4a recq4b fancm r* = 0.925 <2.2×10^−16^) (Fig 6A, S4 and S5 Figs and S14 Table). Crossovers occurred predominantly within the gene transcribed regions and were reduced in the highly polymorphic intergenic regions, in all genotypes (Fig 6A and S4 and S5 Figs). In wild type, highest crossover frequency was observed at the *RAC1* 5'-end, with distinct peaks associated with the first and second exons, in addition to elevated crossovers occurring within the last three LRR domain-encoding exons (Fig 6A and S4 Fig). Crossovers were also evident at the 5′ and 3′ ends of the adjacent gene (*GDSL*), although at a lower level to those observed in *RAC1* (Fig 6A and S4 Fig). In *fancm* the crossover profile was similar, except for in the first *RAC1* exon where crossover frequency was reduced compared to wild type (Fig 6A). In the *recq4a recq4b* and *fancm recq4a recq4b* mutants we observed that the *RAC1* 5′ crossover peaks in exons 1 and 2 were relatively reduced (Fig 6A). The *RAC1* LRR crossover peaks in *recq4a recq4b* and *fancm recq4a recq4b* backgrounds were also less broad and became focused towards the end of exon 5 (Fig 6A). The 5′-end of *GDSL* was reduced in the *recq4a recq4b* and *fancm recq4a recq4b* mutants (Fig 6A). The local changes to crossover frequency in these recombination mutant backgrounds may reflect differential interactions with interhomolog polymorphism or chromatin within the analysed region.

To investigate the relationship between crossovers and polymorphisms, we calculated recombination (crossover read pairs/cM) and polymorphism values in adjacent 250 bp windows, against the *RAC1* Col/Ler panmolecule. Consistent with our previous observations, all genotypes showed a significant negative correlation between crossovers and polymorphisms (Spearman’s: WT *r* = -0.64, *fancm r* = -0.58, *recq4a recq4b r* = -0.57, *fancm recq4a reqc4b r* = -0.57) (S18 Table). As described above, a non-linear model fitted the data using the formula y = log(a)+b×x^(-c), where y is the crossovers, x is polymorphisms, a is the intercept and b and c are scale parameters (Fig 6B). Hence, the suppressive effect of polymorphisms was observed within *RAC1* in both wild type and anti-crossover mutants.

## Discussion

The concentration of meiotic DSBs and crossovers in narrow hotspots is widespread among eukaryotes, which has important implications for genetic diversity and adaptation [78–80]. For example, sequencing of SPO11-oligonucleotides has revealed meiotic DSB hotspots in fungi, animals and plants [66,81–83]. Varying genetic and epigenetic factors control DSB hotspot location in these species. SPO11-oligo hotspots in budding yeast and plants are highest in nucleosome-free regions associated with genes and transposons [66,81,84,85], which demonstrates the importance of chromatin for initiation of meiotic recombination. Variation in nucleosome occupancy and SPO11-1-oligos in plants correlates with AT-sequence richness [66], which is known to exclude nucleosomes [86]. In fission yeast SPO11-oligo hotspots are broader, located intergenically and do not show a clear association with nucleosome occupancy [83]. Mammalian meiotic DSB hotspots are directed to specific DNA sequences by binding of the PRDM9 KRAB-zinc finger protein [80,82,87]. PRDM9 possesses a histone methyltransferase SET domain which directs H3K4me3 and H3K36me3 histone modifications to nucleosomes flanking the bound DNA target sites [82,88–90]. Hence, depending on the species, chromatin and DNA sequence make varying contributions to the fine-scale distributions of meiotic DSBs.

In many species, including budding yeast and plants, there is a positive correlation between meiotic DSB levels and crossover frequency at the chromosome scale [66,81]. However, extensive variation in the ratio of DSBs to crossovers is observed along chromosomes [66,81–83]. Equally, at the fine-scale there is a weak correlation between crossovers and DSB frequency within Arabidopsis hotspot regions [66], as observed at *RAC1* and *RPP13*. An extreme example of similar variation occurs in fission yeast, where an inverse relationship is observed, with DSB hotspots occurring in regions of lowest crossover formation [83,91]. Variation in crossover:non-crossover ratios has also been observed between mammalian hotspots [71,72,78,92]. For example, crossover:non-crossover variation occurs at heterochiasmic mouse hotspots, where DSBs occur in both male and female meiosis, but crossovers only form in male meiosis [93]. Furthermore, data in budding yeast indicate that interhomolog joint molecules may be mobile [94], and repeated rounds of strand invasion and dissolution may occur during repair [95,96], which could cause local differences in the locations of the initiating DSB and final crossover resolution. Hence, the levels of initiating DBSs are important for crossover levels, but they are not the sole determinant of recombination outcomes.

In plants, somatic homologous recombination has been analysed using ‘split GUS’ reporter systems [97]. Recombination between repeated *GUS* sequences located on the same or different reporter T-DNAs restores β-glucuronidase activity [97]. Increasing levels of polymorphism in the recombining *GUS* repeats was found to inhibit homologous recombination [98,99]. For example, 1.9% sequence divergence between the *GUS* repeats caused a 10-fold reduction in somatic recombination [98]. In a related study, a single mismatch in a 618 bp *GUS* repeat caused a 3-fold suppression of recombination, although addition of further SNPs had less effect, suggesting ‘divergence saturation’ in this system [99]. These data are consistent with genetic analysis in budding yeast where mitotic and meiotic recombination are inhibited by polymorphism, with similar kinetics [47,100]. For example, progressive addition of SNPs at the *URA3* hotspot reduced meiotic crossovers, with a simultaneous increase in non-crossover repair [101]. Consistent with these previous studies, at *RAC1* and *RPP13* we observe a non-linear, negative relationship between interhomolog polymorphism and meiotic crossover formation.

A likely mechanism for the suppressive effects of polymorphism on crossover repair during meiosis is via MutS related heterodimers, including MSH2, which are capable of recognising mismatches and promoting disassociation of strand invasion events [44,45,102]. Indeed, evidence exists in Arabidopsis for MSH2 acting as a hybrid-specific anti-crossover factor at the megabase scale [46]. However, this relationship appears complex, as we observe a significantly decreased *RAC1* crossover frequency in the *msh2* mutant. Our observations may suggest regional changes in crossover distributions in *msh2*, rather than a global increase. The inhibitory effect of interhomolog polymorphism on crossover formation may also account for discrepancies observed between SPO11-1-oligos and crossovers at the fine-scale [66]. It is possible that mismatches in interhomolog joint molecules could alter their mobility and further influence the location of crossover resolution. The phenomenon of crossover interference, which reduces the likelihood of adjacent DSBs being repaired as crossovers in the same meiosis, is also important to consider [29].

In addition to interhomolog polymorphism, chromatin marks may differentially influence meiotic recombination pathways and locally alter crossover:noncrossover ratios. For example, we observe that H3K4me3 is elevated at the 5′-ends of *RAC1*, *GDSL* and *RPP13*, which correlates with regions of high crossover activity. Although it is also notable that substantial 3′-crossovers occur in these genes, where H3K4me3 occurs at lower levels. Although H3K4me3 levels do not strongly correlate with SPO11-oligo levels in animals, fungi or plants [66,81,82,103], this mark is spatially associated with recombination hotspots in multiple species [23,59,76,87,104]. In budding yeast the Spp1 subunit of the COMPASS methylase complex simultaneously interacts with H3K4me3 and the Mer2 meiotic chromosome axis component [105,106], providing direct support for the tethered-loop/axis model for recombination [107]. Analogous interactions are observed between mouse COMPASS CXXC1, PRDM9 and the IHO1 axis protein [108]. Hence, the presence of H3K4me3 at the 5′-ends of *RPP13* and *RAC1* may promote crossover formation via similar mechanisms, downstream of DSB formation. Heterochromatic modifications also show specific interactions with the meiotic recombination pathways. For example, in Arabidopsis loss of CG context DNA methylation via the *met1* mutation, or loss of non-CG DNA methylation/H3K9me2 via *cmt3* or *kyp/suvh4 suvh5 suvh6*, both cause an increase in SPO11-1-oligos in pericentromeric heterochromatin [66,109]. However, the CG and non-CG mutants show increased and decreased pericentromeric crossovers, respectively [66,109]. This indicates that despite these heterochromatic mutants showing greater SPO11-1 DSB activity close to the centromeres, other chromatin features likely modify downstream repair choices.

In this study we measured *RAC1* crossover frequency in backgrounds with, (i) elevated *HEI10* dosage and thereby increased Class I activity [51], (ii) increased Class II crossovers via loss of function *fancm*, *figl1* and *recq4a recq4b* anti-crossover mutations [30,33,34,38,49], and (iii) loss of function mutants in the mismatch repair factor *msh2* [46]. Despite these backgrounds showing elevations in crossover frequency elsewhere in the genome, *RAC1* remained resistant to recombination increases or showed small but significant decreases. In this respect it is notable that genome-wide maps of crossovers in *HEI10*, *figl1*, *fancm* and *recq4a recq4b* backgrounds have shown that recombination increases are highly distalized towards the sub-telomeres, which are chromosome regions of lower interhomolog polymorphism [49–51]. Therefore, the location of *RAC1* on the edge of the chromosome 1 pericentromere may make this locus relatively insensitive to distalized crossover increases. It is also possible that high polymorphism levels within *RAC1*, in addition to the surrounding regions of heterochromatin, may contribute to maintenance of stable crossover frequency between wild type and the high recombination backgrounds tested.

The local inhibitory relationship between polymorphism and crossovers that we observe has implications for the evolution of plant hotspots. Data from several species are consistent with meiotic recombination being mutagenic [110–112]. For example, this may occur as a result of DNA polymerase base misincorporation during DSB-repair associated DNA synthesis [110–112], or mis-alignment during strand invasion causing insertions and deletions via unequal crossover [113]. Therefore, high levels of recombination over many generations may cause higher levels of heterozygosity at hotspots, which may then suppress further recombination in specific crosses. Crossover inhibition is likely to be particularly potent when unequal crossover generates large insertion-deletion polymorphisms, which are commonly observed at plant disease resistance loci and can contribute to functional diversity in pathogen recognition [60,113–115].

Despite the negative relationship that we observe between interhomolog polymorphism and crossovers at *RAC1* and *RPP13*, at the chromosome scale wild type crossovers in Arabidopsis show a weak positive relationship with interhomolog diversity [49,50]. Similarly, LD-based historical estimates are positively correlated with population diversity in Arabidopsis [48,55,56]. These population-scale relationships are likely to be partly explained by hitchhiking/background selection, causing more extensive reductions in diversity in regions of low recombination that are under selection [4]. However, other effects may also contribute. For example, in Arabidopsis juxtaposition of megabase scale heterozygous and homozygous regions increases crossover frequency in the heterozygous region, at the expense of the homozygous region [48]. This heterozygosity juxtaposition effect is dependent on the Class I interfering repair pathway [48]. Therefore, both positive and negative interactions are observed between polymorphism and recombination depending on whether hotspot versus chromosome scales are analysed, with significant additional effects caused by chromosome position and chromatin context.

## Material and methods

### Plant material

Arabidopsis lines used in this study were the *HEI10* line ‘C2’ [51], *recq4a-4* (Col, N419423) *recq4b-2* (Col, N511130) [36], *recq4a* (Ler W387*) [34], *fancm-1* (Col, ‘roco1’) [30], *fancm* (Ler, ml20), *figl1-1* (Col, ‘roco5’) [38], *figl1* (Ler, ml80) and *msh2-1* (Col, SALK_002708) [116]. Genotyping of Col *recq4a-4*, Col *recq4b-2*, Ler *recq4a* and *HEI10* T-DNA was performed as described previously [50]. Col and Ler wild type or mutant backgrounds were crossed to obtain F_1_ hybrids, on which pollen-typing was performed. The *msh2-1* allele was introduced into Ler-0 background by six successive backcrosses. Genotyping of *msh2-1* was performed by PCR amplification using msh2-F (5'-AGCGCAATTTGGGCATGTCT-3') and msh2-R (5'-CCTCCCATGTTAGGCCCTGTT-3') oligonucleotides for the wild type allele, and msh2-F and msh2-T-DNA (5'-ATTTTGCCGATTTCGGAAC-3') oligonucleotides for the *msh2-1* allele.

### *RPP13* and *RAC1* pollen-typing and Sanger sequencing

Pollen-typing was performed as described [68]. Genomic DNA was extracted from hybrid F_1_ pollen (Col×Ler, Col×Wl or Col×Mh), and used for nested PCR amplifications using parental or crossover configurations of allele-specific oligonucleotide (ASO) primers (S19 and S20 Tables). For each genotype replicate, ~140 plants were grown and used for pollen collection. The relative concentrations of parental (non-recombinant) and crossover (recombinant) molecules were estimated by titration [68–70]. Recombination rate was calculated using the formula cM = (crossovers/(parentals+crossovers))×100. Amplified single crossover molecules were treated with exonuclease I (NEB, M0293) and shrimp alkaline phosphatase (Amersham, E70092), and then Sanger sequenced to identify recombination sites to the resolution of individual polymorphisms. For analysis we PCR amplified and sequenced the target regions from Col, Ler, Wl and Mh accessions, and used these data to generate Col×Ler, Col×Wl or Col×Mh panmolecules, which include all bases from both accessions (S2 Fig). To analyse the relationship between crossovers and polymorphisms we used adjacent 500 bp windows along the panmolecules and assigned crossover and polymorphism counts, where SNPs were counted as 1, and indels as their length in base pairs. When crossover events were detected in SNP intervals that overlapped window divisions the crossover number was divided by the proportional distance in each window. For example, if two crossovers were detected in a 150 bp interval, of which 50 bp were in window A and 100 bp in window B, we counted 2×(50/150) = 0.67 crossover in window A, and 2×(100/150) = 1.33 crossover in window B. A non-linear model was fitted to the data using the formula; y = log(a)+b×x^(-c), where y is the number of crossovers, x is polymorphisms, a is the intercept and b and c are scale parameters.

### *RAC1* crossover sequencing

Multiple independent *RAC1* crossover PCR amplifications were performed, where each reaction was estimated to contain between 1–3 crossover molecules, based on previous titration experiments. *RAC1* crossover amplification products were then pooled, concentrated by isopropanol precipitation and gel purified. 1–2 μg of DNA in 100 μl of TE was sonicated for each sample using a Bioruptor (Diagenode) (high setting, 30 seconds ON, 30 seconds OFF for 15 minutes), and fragments of 300–400 bp were gel purified, end-repaired and used to generate sequencing libraries (Tru-Seq, Illumina). The libraries were sequenced on an NextSeq instrument (Illumina) using paired-end 75 bp reads. Reads were aligned to the parental sequences (Col and Ler) using Bowtie, allowing only exact matches [77]. Reads were filtered for those that aligned to one parental sequence only. To identify crossover read pairs, we filtered for read pairs having a centromere-proximal match to Col and a centromere-distal match to Ler, on opposite strands, which is consistent with *RAC1* pollen-typing amplification. Read pair coordinates were then converted into pancoordinates using the Col/Ler key table (S2 Table). A value of 1 was assigned to all panmolecule coordinates between each crossover read pair. This process is repeated for all read pairs and values normalized by the total number of crossover read pairs, and finally weighted by genetic distance (cM).

### Data access

The fastq files associated with *RAC1* crossover sequencing have been uploaded to ArrayExpress accession E-MTAB-6333 “Meiotic crossover landscape within the *RAC1* disease resistance gene”.

## Supporting information

S1 Fig *RPP13* allele specific oligonucleotide PCR amplification. **(A)** Representative ethidium bromide-stained agarose gels showing optimisation of allele-specific amplification of *RPP13*. The indicated allele-specific oligonucleotides (ASOs) were used with universal primers (UF and UR) on either Col or Ler genomic DNA templates. A range of annealing temperatures were used, which are printed above the gel in green. **(B)** *RPP13* crossover molecule amplification was performed from leaf or pollen DNA extracted from Col/Ler F_1_ plants. PCR bands of crossover molecules were detected strongly in pollen ampiflications, but not using leaf DNA (upper). A control PCR amplification for input DNA amount is shown by amplifying with Col-ASO forward and reverse primers (lower), which amplifies parental molecules.
(TIF)

S2 Fig *RAC1* and *RPP13* panmolecules. Plots representing panmolecules for *RAC1* from Col×Ler, Col×Mh and Col×Wl crossoes and *RPP13* from a Col×Ler cross. The panmolecule coordinates are shown along the x-axis and start relative to the cognate position in the TAIR10 Col reference sequence. The position of single nucleotide polymorphisms (SNPs) are indicated by red dots along the plot. Indels are indicated by deviation of the plot line either above (Col) or below (Ler, Mh or Wl) the axis, with indel length indicated by the length of the deviation.
(TIF)

S3 Fig Analysis of crossover sequencing libraries and read-pairs. **(A)** Ethidium bromide stained agarose gel showing the size of final crossover libraries. The library is shown before adapter ligation (‘-‘) and after adapter ligation, PCR amplification and size selection (‘+’). The shift on the gel corresponds to the ligation of adapters (2×60 bp = 120 bp). Libraries constructed using ~300 or ~1,000 independent crossover molecules are indicated. **(B)** Histograms showing the size distribution of the distance between filtered crossover reads, according to genotype and crossover library. Libraries were constructed using either ~300 or ~1,000 independent crossover molecules and analysed separately. The red dotted lines represent the mean crossover read distances for each library.
(TIF)

S4 Fig Crossover frequency across *RAC1* in wild type and *fancm*, *recq4a recq4b* and *fancm recq4a recq4b* mutants analysing ~300 or ~1,000 crossovers. The coverage of crossover reads aligned against the Col×Ler panmolecule was calculated and normalized by the total number of reads analysed, and also by *RAC1* genetic distance (cM), measured previously by titration. For each genotype two biological replicate libraries were analysed, constructed with amplifications from an estimated ~300 (purple) or ~1,000 (black) independent crossover molecules. Col×Ler polymorphisms are indicated by black ticks on the x-axis. Gene TSS and TTS are indicated by vertical blue lines, and exons by horizontal black lines.
(TIF)

S5 Fig Comparison of Sanger and NGS-derived crossover maps within the *RAC1* amplicon in wild type, *fancm*, *recq4a recq4b* and *fancm recq4a recq4b*. Crossover frequency (cM/Mb) within the region of the *RAC1* disease resistance gene measured using titration and Sanger sequencing of single crossover molecules from Col×Ler wild type (0.095 cM), *recq4a recq4b* (0.059 cM), *fancm* (0.083 cM) and *recq4a recq4b fancm* (0.55 cM) pollen F_1_ genomic DNA. Recombination is plotted against the panmolecules, which include all bases from both parental accessions. Gene TSS/TTS are indicated by vertical dotted lines and exons by horizontal black lines. The position of *RAC1* TIR (green), NB-ARC (red) and LRR (blue) domain-encoding sequences are indicated by the horizontal lines. SNPs (red) and indels (black) are indicated by the ticks on the x-axis. On the right, the Sanger data are overlaid with the coverage of crossover reads normalized by the total number of reads analysed and also normalized by *RAC1* genetic distance (cM) (red). Col×Ler polymorphisms are indicated by black ticks on the x-axis.
(TIF)

S1 Table Recombination rate calculated via pollen-typing across the *RPP13* disease resistance gene in Col×Ler. The panmolecule physical distance between the inner pollen-typing ASOs is 5,626 bp.
(DOCX)

S2 Table *RAC1* Col/Ler pangenome key. See separate file ‘S2_Table_Col_Ler_RAC1_key.csv’. The file lists panmolecule coordinates with the cognate position in the Col and Ler templates. The ‘SNP’ column indicates SNP positions by ‘1’ values. The ‘LER insertion’ column indicates the position of additional bases in Ler compared to Col, which are indicated by ‘1’ values, whereas the ‘COL insertion’ column indicates the position of Col inserted bases compared to Ler by ‘2’ values.
(CSV)

S3 Table *RAC1* Col/Wl pangenome key. See separate file ‘S3_Table_Col_Wl_RAC1_key.csv’. The file lists panmolecule coordinates with the cognate position in the Col and Wl templates. The ‘SNP’ column indicates SNP positions by ‘1’ values. The ‘Wl insertion’ column indicates the position of additional bases in Wl compared to Col, which are indicated by ‘1’ values, whereas the ‘COL insertion’ column indicates the position of Col inserted bases compared to Wl by ‘2’ values.
(CSV)

S4 Table *RAC1* Col/Mh pangenome key. See separate file ‘S4_Table_Col_Mh_RAC1_key.csv’. The file lists panmolecule coordinates with the cognate position in the Col and Mh templates. The ‘SNP’ column indicates SNP positions by ‘1’ values. The ‘MH insertion’ column indicates the position of additional bases in Mh compared to Col, which are indicated by ‘1’ values, whereas the ‘COL insertion’ column indicates the position of Col inserted bases compared to Mh by ‘2’ values.
(CSV)

S5 Table *RPP13* Col/Ler pangenome key. See separate file ‘S2_Table_Col_Ler_RPP13_key.csv’. The file lists panmolecule coordinates with the cognate position in the Col and Ler templates. The ‘LER insertion’ column indicates the position of additional bases in Ler compared to Col, which are indicated by ‘1’ values, whereas the ‘COL insertion’ column indicates the position of Col inserted bases compared to Ler by ‘2’ values.
(CSV)

S6 Table Crossover distributions within the *RPP13* amplicon from Col×Ler F_1_ analysed via pollen-typing. Interval lengths are calculated according to the panmolecule, and these distances are used to calculate cM/Mb.
(DOCX)

S7 Table Recombination rate calculated via pollen-typing across the *RAC1* disease resistance gene in Col×Ler and Col×Mh. Recombination rate (cM/Mb) was calculated by dividing genetic distance (cM) by panmolecule physical length. A chi-square test using a 2×2 contingency table was used to test for a significant difference between the genotypes.
(DOCX)

S8 Table Crossover distributions across the *RAC1* amplicon from Col×Ler F_1_ analysed via pollen-typing. We have combined 181 crossovers reported previously with an additional 59, to give a new set of 240 crossovers. Crossover frequency (cM/Mb) was calculated using Col×Ler F_1_ titration data genetic distance (0.074 cM). Interval lengths are calculated according to the panmolecule, and these distances are used to calculate cM/Mb.
(DOCX)

S9 Table Crossover distributions across the *RAC1 R* gene hotspot in Col×Wl F_1_ analysed via pollen-typing. Crossover frequency (cM/Mb) was calculated using Col×Ler F_1_ titration data genetic distance (0.074 cM), and interval lengths according to the panmolecule.
(DOCX)

S10 Table Crossover distributions within the *RAC1* amplicon from Col×Mh F_1_ analysed via pollen-typing. Crossover frequency (cM/Mb) was calculated using Col×Mh F_1_ titration data genetic distance (0.064 cM), and interval lengths according to the panmolecule.
(DOCX)

S11 Table Adjacent window analysis of polymorphisms versus crossovers within the *RAC1* pollen-typing amplicon. The window size used was 500 bp, within which crossovers (CO) were counted, and the number of polymorphisms (Polys.), where SNPs were counted as one and indels by their length in bp. Analysis was performed against the pancoordinates. The row highlighted in grey is the final window which has a different size in each cross due to variable panmolecule lengths. This window was not included in the analysis plotted in Fig 4.
(DOCX)

S12 Table Adjacent window analysis of SNPs vs crossovers within the *RPP13* pollen-typing amplicon. The window size used was 500 bp, within which crossovers were counted, and the number of polymorphisms, where SNPs were counted as one and indels by their length in bp. Analysis was performed against the pancoordinates. The row highlighted in grey is the final window which has a different size in each cross due to variable panmolecule lengths. This window was not included in the analysis plotted in Fig 4.
(DOCX)

S13 Table Genetic distance within the *RAC1* amplicon in wild type and genetic backgrounds with altered meiotic recombination. Recombination rate was calculated using the Col×Ler panmolecule distance between the pollen-typing inner ASOs (9,482 bp).
(DOCX)

S14 Table Crossover distributions across the *RAC1 R* gene hotspot in Col×Ler F_1_ analysed via pollen-typing in wild type, *recq4a recq4b*, *fancm* and *recq4a recq4b fancm*. Crossover events were identified using Sanger sequencing of amplifications from single molecules. Interval lengths are calculated according to the Col×Ler panmolecule, and these distances are used to calculate cM/Mb.
(DOCX)

S15 Table Genetic distance of the *RAC1* amplicon in wild type and *msh2* mutant in Col×Ler. Recombination rate was calculated using the Col×Ler panmolecule distance between the pollen-typing inner ASOs (9,482 bp).
(DOCX)

S16 Table Significance testing of genetic distance of the *RAC1* amplicon in wild type and mutant backgrounds. We used the mean crossovers and parentals from wild type and mutants to construct 2×2 contingeny tables and perform Chi-square tests.
(DOCX)

S17 Table Mapping reads and filtering during *RAC1* pollen-seq in wild type and *fancm*, *recq4a recq4b* and *fancm recq4a recq4b* mutants. The ‘Total’ column lists the numbers of read pairs obtained. The number of read pairs surviving sequential analysis filters are listed in order to identify *RAC1* crossover read pairs. Paired end reads (end1 and end2) were separated and aligned to the Col or Ler *RAC1* parental template sequences, allowing only exact matches (Mapped). Read pair ends that mapped uniquely to either Col or Ler were kept (Unique). Read pair ends (1 and 2) that mapped to Col and Ler were identified (Matched), where the Ler mapping read had a lower coordinate than the Col mapping read (Orientate), and that were on opposite strands (Strand). Table (A) shows the reads obtained from libraries generated from ~300 crossovers from wild type, *fancm*, *recq4a recq4b* and *fancm recq4a recq4b*, while Table (B) shows those obtained from ~1,000 crossover libraries for the same genotypes.
(DOCX)

S18 Table Correlation between polymorphisms and crossover reads in pollen-sequencing data. Using adjacent windows of the indicated size, correlations (Spearman’s) were performed against crossover reads pairs and polymorphism density calculated against the Col×Ler panmolecule. *P* values are printed below the correlation coefficient in parentheses.
(DOCX)

S19 Table Pollen-typing allele specific primers for *RAC1* and *RPP13*. Red highlighting indicates the position of a Col/Ler SNP. If the oligo sequence is entirely red, it hybridizes to a sequence only present in one accession (indel).
(DOCX)

S20 Table *RAC1* and *RPP13* pollen typing PCR parameters. Primer combinations are listed for use in *RAC1* and *RPP13* pollen typing amplifications, together with the PCR parameters used.
(DOCX)
