# Supplementary figures and images for: Interhomolog polymorphism shapes meiotic crossover within the Arabidopsis RAC1 and RPP13 disease resistance genes
Source: PLoS Genet. 2018 Dec 13;14(12):e1007843. doi: 10.1371/journal.pgen.1007843 (PMC6307820; doi:10.1371/journal.pgen.1007843)

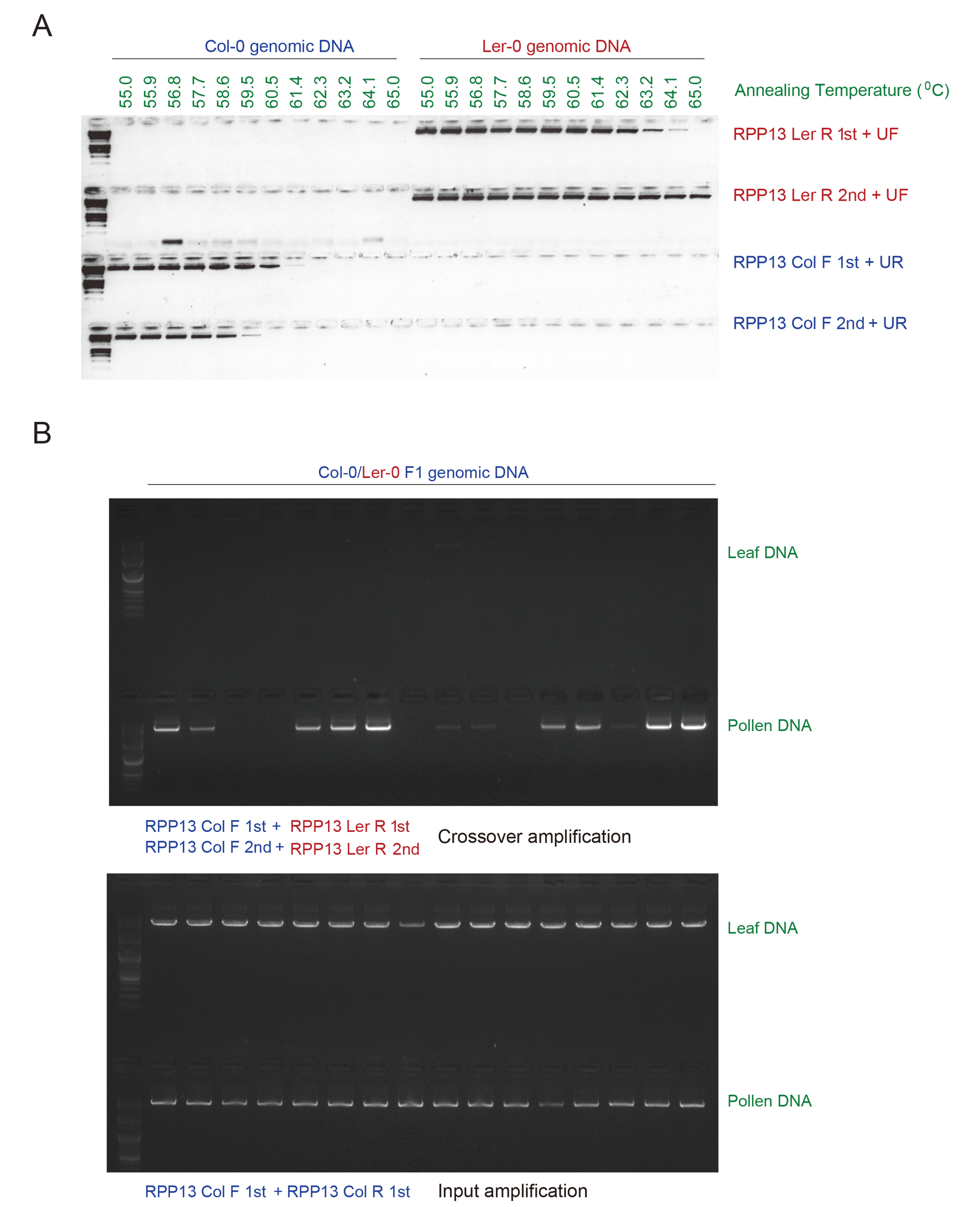

Supplement: S1 Fig — (A) Representative ethidium bromide-stained agarose gels showing optimisation of allele-specific amplification of RPP13. The indicated allele-specific oligonucleotides (ASOs) were used with universal primers (UF and UR) on either Col or Ler genomic DNA templates. A range of annealing temperatures were used, which are printed above the gel in green. (B) RPP13 crossover molecule amplification was performed from leaf or pollen DNA extracted from Col/Ler F1 plants. PCR bands of crossover molecules were detected strongly in pollen ampiflications, but not using leaf DNA (upper). A control PCR amplification for input DNA amount is shown by amplifying with Col-ASO forward and reverse primers (lower), which amplifies parental molecules. (TIF) [file pgen.1007843.s001.tif]

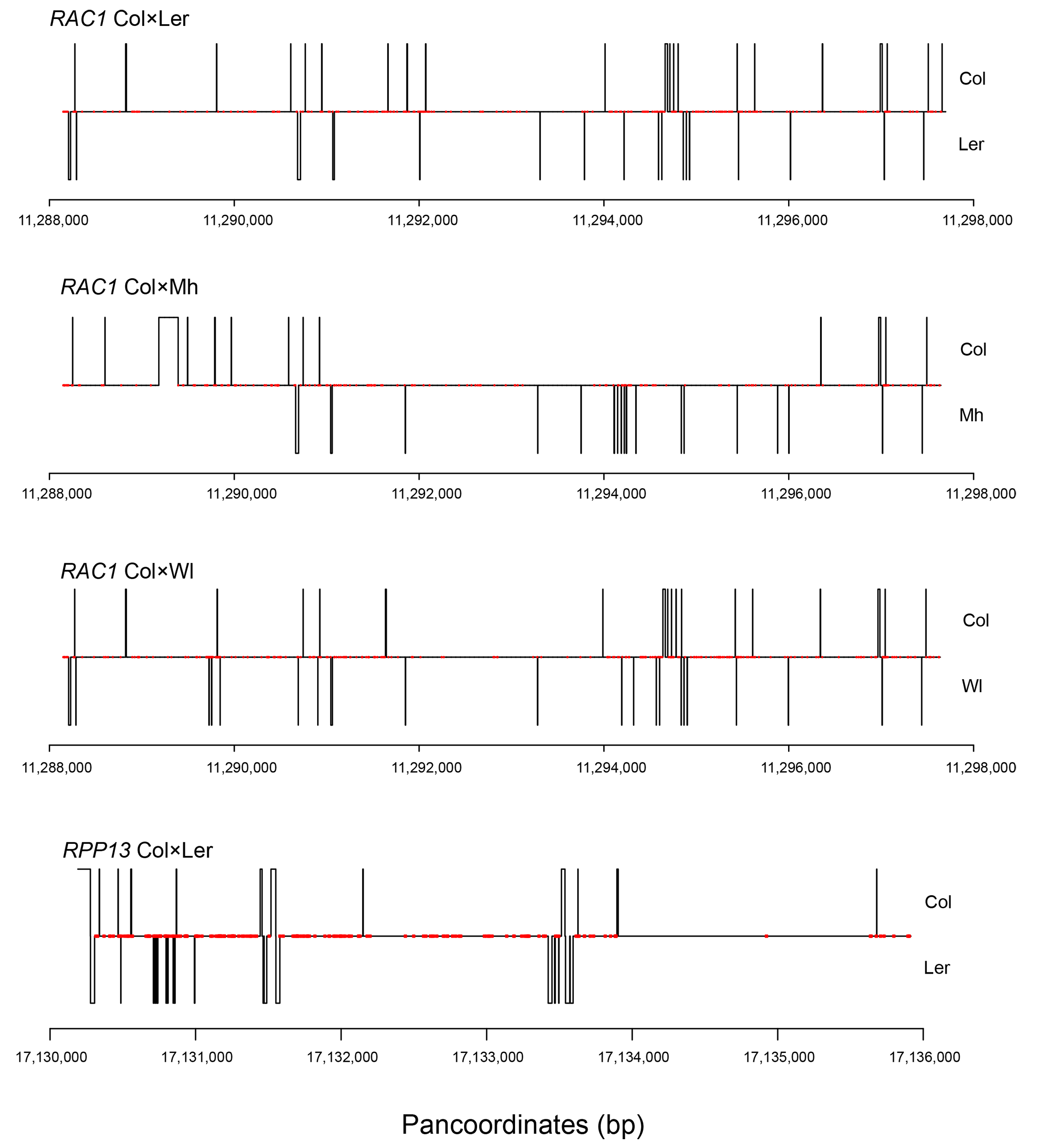

Supplement: S2 Fig — Plots representing panmolecules for RAC1 from Col×Ler, Col×Mh and Col×Wl crossoes and RPP13 from a Col×Ler cross. The panmolecule coordinates are shown along the x-axis and start relative to the cognate position in the TAIR10 Col reference sequence. The position of single nucleotide polymorphisms (SNPs) are indicated by red dots along the plot. Indels are indicated by deviation of the plot line either above (Col) or below (Ler, Mh or Wl) the axis, with indel length indicated by the length of the deviation. (TIF) [file pgen.1007843.s002.tif]

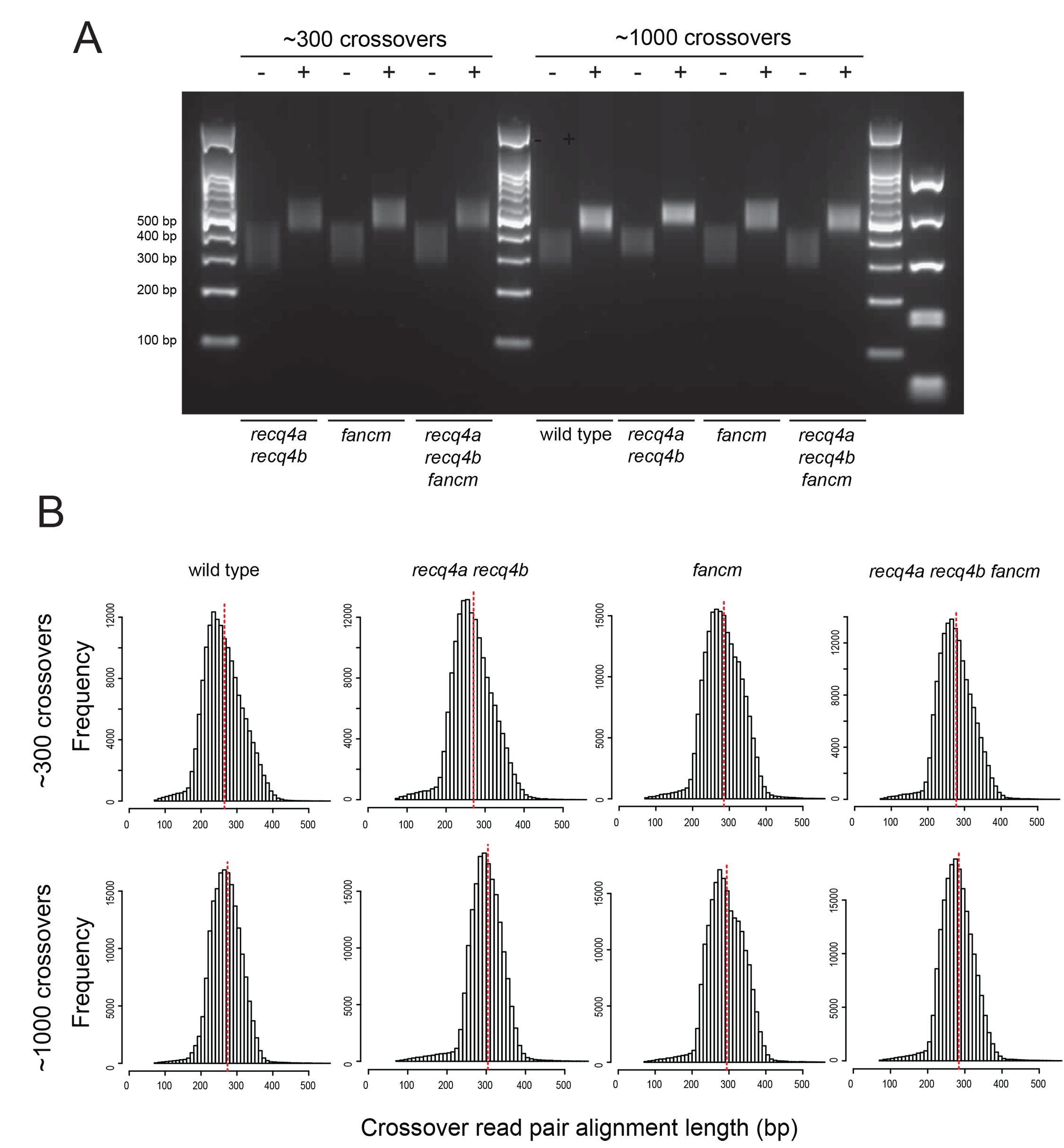

Supplement: S3 Fig — (A) Ethidium bromide stained agarose gel showing the size of final crossover libraries. The library is shown before adapter ligation (‘-‘) and after adapter ligation, PCR amplification and size selection (‘+’). The shift on the gel corresponds to the ligation of adapters (2×60 bp = 120 bp). Libraries constructed using ~300 or ~1,000 independent crossover molecules are indicated. (B) Histograms showing the size distribution of the distance between filtered crossover reads, according to genotype and crossover library. Libraries were constructed using either ~300 or ~1,000 independent crossover molecules and analysed separately. The red dotted lines represent the mean crossover read distances for each library. (TIF) [file pgen.1007843.s003.tif]

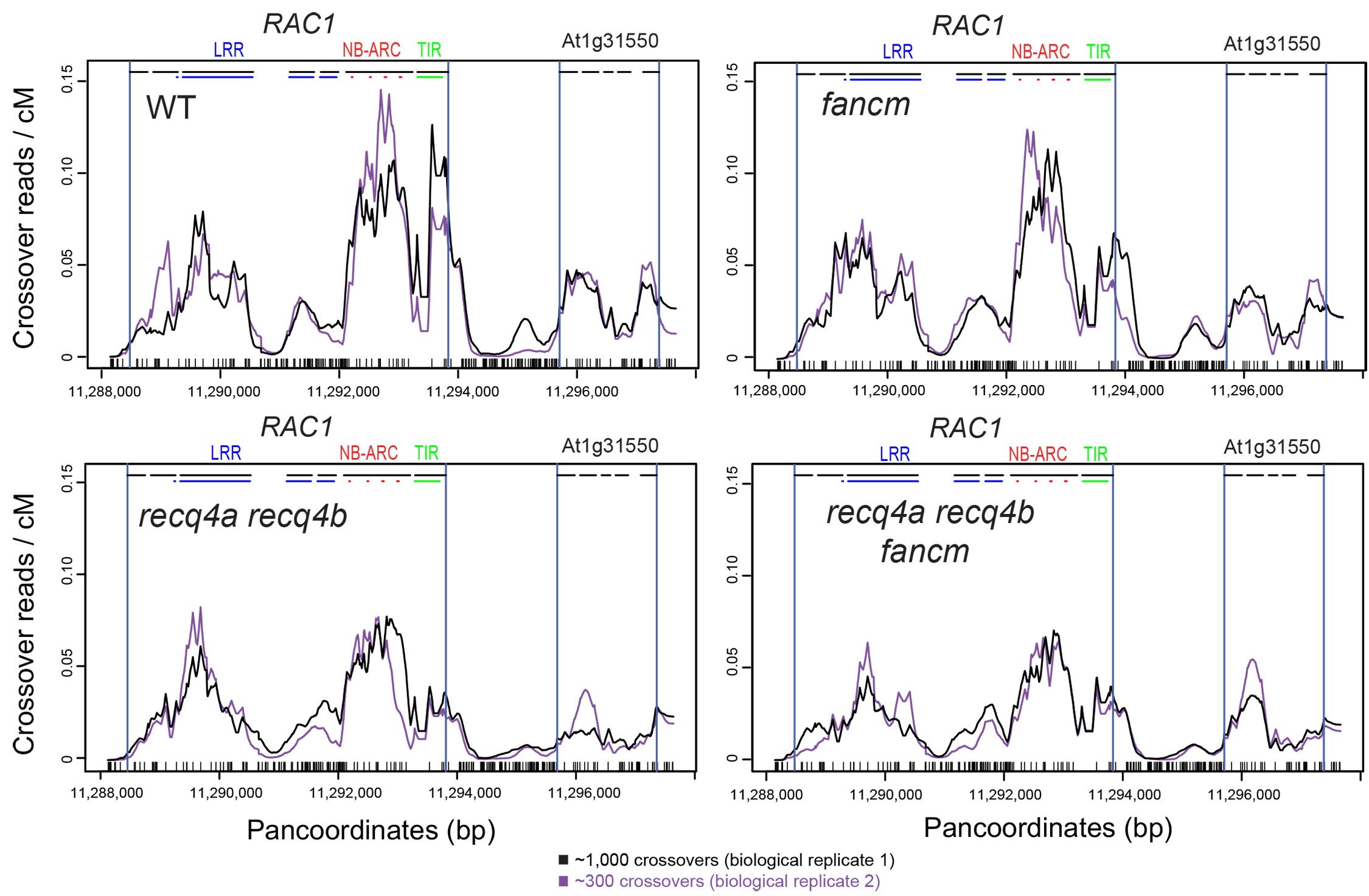

Supplement: S4 Fig — The coverage of crossover reads aligned against the Col×Ler panmolecule was calculated and normalized by the total number of reads analysed, and also by RAC1 genetic distance (cM), measured previously by titration. For each genotype two biological replicate libraries were analysed, constructed with amplifications from an estimated ~300 (purple) or ~1,000 (black) independent crossover molecules. Col×Ler polymorphisms are indicated by black ticks on the x-axis. Gene TSS and TTS are indicated by vertical blue lines, and exons by horizontal black lines. (TIF) [file pgen.1007843.s004.tif]

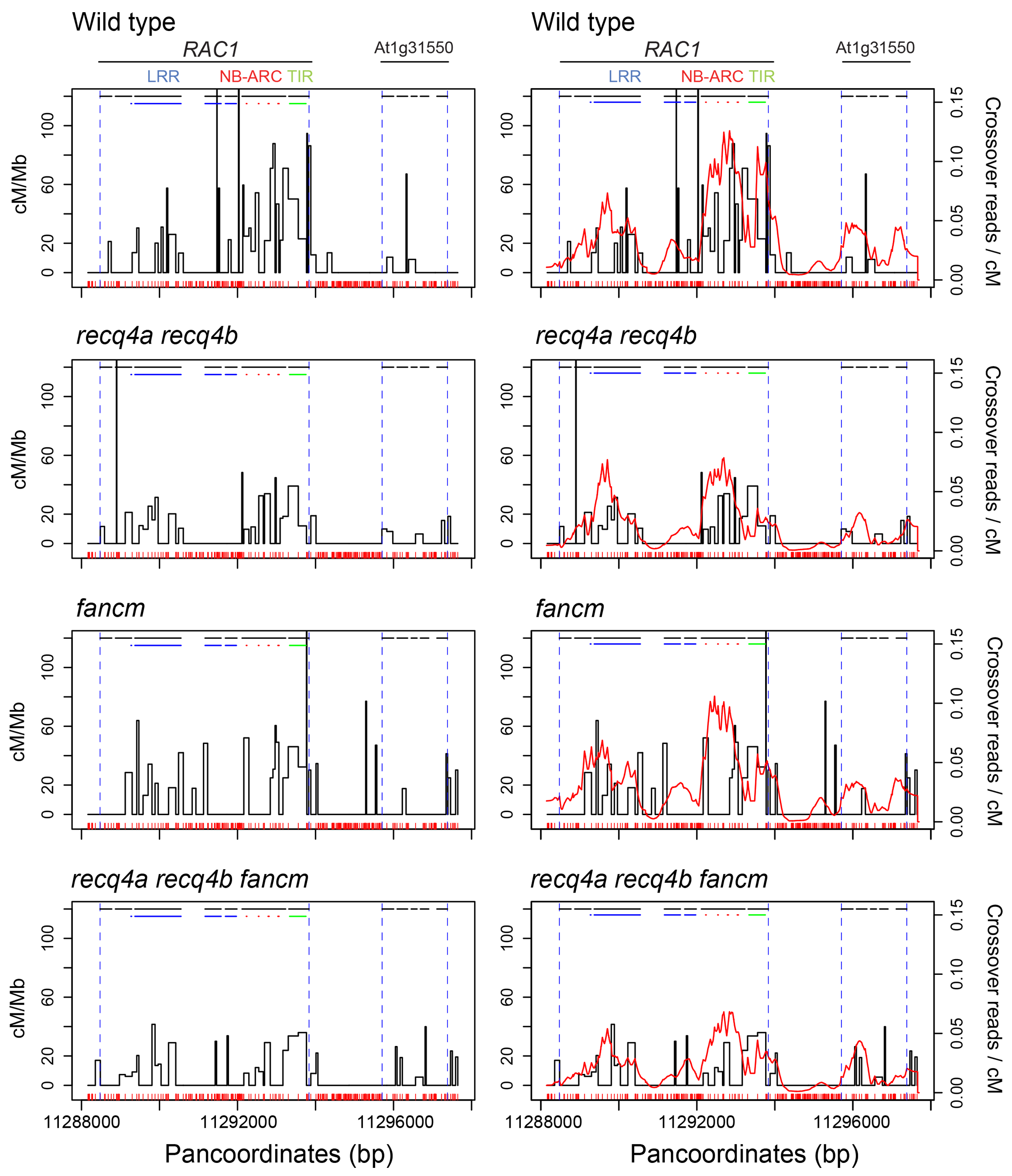

Supplement: S5 Fig — Crossover frequency (cM/Mb) within the region of the RAC1 disease resistance gene measured using titration and Sanger sequencing of single crossover molecules from Col×Ler wild type (0.095 cM), recq4a recq4b (0.059 cM), fancm (0.083 cM) and recq4a recq4b fancm (0.55 cM) pollen F1 genomic DNA. Recombination is plotted against the panmolecules, which include all bases from both parental accessions. Gene TSS/TTS are indicated by vertical dotted lines and exons by horizontal black lines. The position of RAC1 TIR (green), NB-ARC (red) and LRR (blue) domain-encoding sequences are indicated by the horizontal lines. SNPs (red) and indels (black) are indicated by the ticks on the x-axis. On the right, the Sanger data are overlaid with the coverage of crossover reads normalized by the total number of reads analysed and also normalized by RAC1 genetic distance (cM) (red). Col×Ler polymorphisms are indicated by black ticks on the x-axis. (TIF) [file pgen.1007843.s005.tif]
