# Supplementary material for: Interhomolog polymorphism shapes meiotic crossover within the Arabidopsis RAC1 and RPP13 disease resistance genes
Source: PLoS Genet. 2018 Dec 13;14(12):e1007843. doi: 10.1371/journal.pgen.1007843 (PMC6307820; doi:10.1371/journal.pgen.1007843)
Supplement: S1 Table — The panmolecule physical distance between the inner pollen-typing ASOs is 5,626 bp. (DOCX) [file pgen.1007843.s006.docx]

**S1 Table. Recombination rate calculated via pollen-typing across the *RPP13* disease resistance gene in Col×Ler.**

| Parentals/μl | 13,632 |
| --- | --- |
| Crossovers/μl | 7.53 |
| cM | 0.055 |
| cM S.D. | 0.009 |
| cM/Mb | 9.78 |
