# Supplementary material for: Interhomolog polymorphism shapes meiotic crossover within the Arabidopsis RAC1 and RPP13 disease resistance genes
Source: PLoS Genet. 2018 Dec 13;14(12):e1007843. doi: 10.1371/journal.pgen.1007843 (PMC6307820; doi:10.1371/journal.pgen.1007843)
Supplement: S6 Table — Interval lengths are calculated according to the panmolecule, and these distances are used to calculate cM/Mb. (DOCX) [file pgen.1007843.s011.docx]

**S6 Table. Crossover distributions within the *RPP13* amplicon from Col×Ler F_1_ analysed via pollen-typing.**

| TAIR coordinate | Pan coordinate | Col | Ler | Interval length (bp) | Crossovers | cM/Mb |
| --- | --- | --- | --- | --- | --- | --- |
| 17130191 | 17130191 | TAGTATACACCCATTATCTTCTTCTGAGTTTCCTCTCTGTCTCTGCTTAGTTTTTTTCAAGCTTGGACCTCGATTTCATTTAAATC | - | 87 | 0 | 0 |
| - | 17130278 | - | AGTATACACCCATTATTTCATTAAAACA | 41 | 0 | 0 |
| 17130291 | 17130319 | C | G | 19 | 0 | 0 |
| 17130310 | 17130338 | AT | - | 37 | 0 | 0 |
| 17130347 | 17130375 | G | T | 39 | 0 | 0 |
| 17130386 | 17130414 | G | T | 3 | 0 | 0 |
| 17130389 | 17130417 | A | T | 22 | 0 | 0 |
| 17130411 | 17130439 | C | T | 28 | 0 | 0 |
| 17130439 | 17130467 | TT | - | 9 | 0 | 0 |
| 17130448 | 17130476 | T | C | 6 | 0 | 0 |
| 17130454 | 17130482 | G | A | 4 | 0 | 0 |
| - | 17130486 | - | A | 7 | 0 | 0 |
| 17130464 | 17130493 | T | A | 23 | 0 | 0 |
| 17130487 | 17130516 | A | T | 17 | 0 | 0 |
| 17130504 | 17130533 | T | C | 7 | 0 | 0 |
| 17130511 | 17130540 | T | A | 8 | 0 | 0 |
| 17130519 | 17130548 | G | C | 7 | 0 | 0 |
| 17130526 | 17130555 | TTCTT | - | 11 | 0 | 0 |
| 17130537 | 17130566 | A | T | 4 | 0 | 0 |
| 17130541 | 17130570 | G | A | 94 | 0 | 0 |
| 17130635 | 17130664 | G | A | 12 | 0 | 0 |
| 17130647 | 17130676 | C | T | 1 | 0 | 0 |
| 17130648 | 17130677 | T | C | 4 | 0 | 0 |
| 17130652 | 17130681 | G | A | 1 | 0 | 0 |
| 17130653 | 17130682 | T | A | 6 | 0 | 0 |
| 17130659 | 17130688 | G | T | 2 | 0 | 0 |
| 17130661 | 17130690 | C | A | 20 | 0 | 0 |
| - | 17130710 | - | TCTAG | 7 | 0 | 0 |
| 17130683 | 17130717 | C | T | 2 | 0 | 0 |
| 17130685 | 17130719 | T | A | 3 | 0 | 0 |
| - | 17130722 | - | TGACT | 8 | 0 | 0 |
| 17130691 | 17130730 | T | C | 1 | 0 | 0 |
| 17130692 | 17130731 | T | A | 2 | 0 | 0 |
| - | 17130733 | - | AGACAAAT | 11 | 0 | 0 |
| 17130697 | 17130744 | C | A | 4 | 0 | 0 |
| 17130701 | 17130748 | G | T | 11 | 0 | 0 |
| 17130712 | 17130759 | T | C | 1 | 0 | 0 |
| 17130713 | 17130760 | T | C | 3 | 0 | 0 |
| 17130716 | 17130763 | C | T | 2 | 0 | 0 |
| 17130718 | 17130765 | T | A | 29 | 0 | 0 |
| 17130747 | 17130794 | A | T | 1 | 0 | 0 |
| 17130748 | 17130795 | G | A | 2 | 0 | 0 |
| - | 17130797 | - | CTT | 5 | 0 | 0 |
| 17130752 | 17130802 | T | C | 1 | 0 | 0 |
| 17130753 | 17130803 | G | T | 4 | 0 | 0 |
| - | 17130807 | - | TCAT | 5 | 0 | 0 |
| 17130758 | 17130812 | A | C | 11 | 0 | 0 |
| 17130769 | 17130823 | A | T | 23 | 0 | 0 |
| - | 17130846 | G | AA | 4 | 0 | 0 |
| 17130795 | 17130850 | C | T | 4 | 0 | 0 |
| - | 17130854 | - | AATTC | 8 | 0 | 0 |
| 17130802 | 17130862 | C | G | 5 | 0 | 0 |
| 17130807 | 17130867 | CAC | - | 8 | 0 | 0 |
| 17130815 | 17130875 | T | A | 1 | 0 | 0 |
| 17130816 | 17130876 | C | G | 15 | 0 | 0 |
| 17130831 | 17130891 | T | G | 10 | 0 | 0 |
| 17130841 | 17130901 | C | T | 31 | 0 | 0 |
| 17130872 | 17130932 | C | T | 1 | 0 | 0 |
| 17130873 | 17130933 | T | C | 4 | 0 | 0 |
| 17130877 | 17130937 | T | G | 3 | 0 | 0 |
| 17130880 | 17130940 | C | G | 3 | 0 | 0 |
| 17130883 | 17130943 | G | A | 1 | 0 | 0 |
| 17130884 | 17130944 | T | C | 1 | 0 | 0 |
| 17130885 | 17130945 | C | A | 47 | 0 | 0 |
| - | 17130992 | - | CCT | 13 | 0 | 0 |
| 17130942 | 17131005 | T | G | 5 | 0 | 0 |
| 17130947 | 17131010 | C | T | 3 | 0 | 0 |
| 17130950 | 17131013 | A | T | 1 | 0 | 0 |
| 17130951 | 17131014 | A | C | 11 | 0 | 0 |
| 17130962 | 17131025 | T | C | 23 | 0 | 0 |
| 17130985 | 17131048 | C | G | 61 | 0 | 0 |
| 17131046 | 17131109 | C | G | 10 | 0 | 0 |
| 17131056 | 17131119 | C | T | 25 | 0 | 0 |
| 17131081 | 17131144 | T | C | 10 | 0 | 0 |
| 17131091 | 17131154 | G | C | 7 | 0 | 0 |
| 17131098 | 17131161 | G | A | 4 | 0 | 0 |
| 17131102 | 17131165 | T | G | 1 | 0 | 0 |
| 17131103 | 17131166 | T | C | 3 | 0 | 0 |
| 17131106 | 17131169 | G | C | 1 | 0 | 0 |
| 17131107 | 17131170 | T | A | 1 | 0 | 0 |
| 17131108 | 17131171 | G | C | 1 | 0 | 0 |
| 17131109 | 17131172 | G | T | 1 | 0 | 0 |
| 17131110 | 17131173 | G | T | 3 | 0 | 0 |
| 17131113 | 17131176 | T | C | 2 | 0 | 0 |
| 17131115 | 17131178 | G | C | 1 | 0 | 0 |
| 17131116 | 17131179 | C | T | 7 | 0 | 0 |
| 17131123 | 17131186 | C | T | 13 | 0 | 0 |
| 17131136 | 17131199 | C | T | 3 | 0 | 0 |
| 17131139 | 17131202 | T | C | 1 | 0 | 0 |
| 17131140 | 17131203 | C | T | 29 | 0 | 0 |
| 17131169 | 17131232 | T | G | 16 | 0 | 0 |
| 17131185 | 17131248 | T | C | 5 | 0 | 0 |
| 17131190 | 17131253 | C | G | 5 | 0 | 0 |
| 17131195 | 17131258 | C | A | 1 | 0 | 0 |
| 17131196 | 17131259 | A | T | 1 | 0 | 0 |
| 17131197 | 17131260 | T | A | 5 | 0 | 0 |
| 17131202 | 17131265 | G | T | 2 | 0 | 0 |
| 17131204 | 17131267 | A | G | 4 | 0 | 0 |
| 17131208 | 17131271 | C | T | 30 | 0 | 0 |
| 17131238 | 17131301 | G | T | 24 | 0 | 0 |
| 17131262 | 17131325 | A | G | 12 | 0 | 0 |
| 17131274 | 17131337 | G | C | 21 | 0 | 0 |
| 17131295 | 17131358 | A | G | 18 | 0 | 0 |
| 17131313 | 17131376 | C | T | 8 | 0 | 0 |
| 17131321 | 17131384 | A | G | 3 | 0 | 0 |
| 17131324 | 17131387 | A | T | 21 | 0 | 0 |
| 17131345 | 17131408 | T | G | 15 | 0 | 0 |
| 17131360 | 17131423 | G | T | 21 | 0 | 0 |
| 17131381 | 17131444 | GAAATAATTGTCA | - | 20 | 0 | 0 |
| - | 17131464 | - | CACTAG | 7 | 0 | 0 |
| - | 17131471 | - | TGCATCTAGAGATGCATC | 39 | 0 | 0 |
| 17131423 | 17131510 | C | T | 8 | 0 | 0 |
| 17131431 | 17131518 | TAATAGCTGCTATATCAAAATCGTTGATAGAAT | - | 34 | 0 | 0 |
| - | 17131552 | - | AAAAGCTAGGTAATAGCTGAAATAATA | 32 | 0 | 0 |
| 17131470 | 17131584 | C | G | 22 | 0 | 0 |
| 17131492 | 17131606 | G | T | 68 | 0 | 0 |
| 17131560 | 17131674 | A | T | 8 | 0 | 0 |
| 17131568 | 17131682 | A | G | 6 | 0 | 0 |
| 17131574 | 17131688 | A | C | 12 | 0 | 0 |
| 17131586 | 17131700 | T | C | 1 | 0 | 0 |
| 17131587 | 17131701 | G | A | 1 | 0 | 0 |
| 17131588 | 17131702 | G | C | 1 | 0 | 0 |
| 17131589 | 17131703 | T | C | 2 | 0 | 0 |
| 17131591 | 17131705 | G | T | 1 | 0 | 0 |
| 17131592 | 17131706 | A | G | 17 | 0 | 0 |
| 17131609 | 17131723 | G | A | 10 | 0 | 0 |
| 17131619 | 17131733 | A | G | 15 | 0 | 0 |
| 17131634 | 17131748 | C | T | 1 | 0 | 0 |
| 17131635 | 17131749 | G | A | 20 | 0 | 0 |
| 17131655 | 17131769 | T | C | 4 | 0 | 0 |
| 17131659 | 17131773 | A | T | 2 | 0 | 0 |
| 17131661 | 17131775 | T | A | 11 | 0 | 0 |
| 17131672 | 17131786 | C | T | 38 | 0 | 0 |
| 17131710 | 17131824 | C | G | 69 | 0 | 0 |
| 17131779 | 17131893 | C | G | 27 | 0 | 0 |
| 17131806 | 17131920 | C | G | 6 | 0 | 0 |
| 17131812 | 17131926 | C | T | 28 | 0 | 0 |
| 17131840 | 17131954 | A | G | 8 | 0 | 0 |
| 17131848 | 17131962 | G | A | 14 | 0 | 0 |
| 17131862 | 17131976 | T | G | 21 | 0 | 0 |
| 17131883 | 17131997 | A | G | 3 | 0 | 0 |
| 17131886 | 17132000 | A | T | 1 | 0 | 0 |
| 17131887 | 17132001 | T | C | 9 | 0 | 0 |
| 17131896 | 17132010 | C | T | 13 | 0 | 0 |
| 17131909 | 17132023 | A | C | 9 | 0 | 0 |
| 17131918 | 17132032 | T | G | 25 | 0 | 0 |
| 17131943 | 17132057 | A | C | 2 | 0 | 0 |
| 17131945 | 17132059 | G | A | 15 | 0 | 0 |
| 17131960 | 17132074 | C | T | 50 | 0 | 0 |
| 17132010 | 17132124 | G | A | 1 | 0 | 0 |
| 17132011 | 17132125 | C | G | 9 | 0 | 0 |
| 17132020 | 17132134 | A | G | 15 | 0 | 0 |
| 17132035 | 17132149 | AAT | - | 34 | 0 | 0 |
| 17132069 | 17132183 | C | T | 20 | 1 | 63 |
| 17132089 | 17132203 | A | C | 243 | 1 | 5 |
| 17132332 | 17132446 | C | T | 57 | 2 | 44 |
| 17132389 | 17132503 | G | C | 68 | 0 | 0 |
| 17132457 | 17132571 | A | G | 16 | 0 | 0 |
| 17132473 | 17132587 | C | T | 31 | 0 | 0 |
| 17132504 | 17132618 | A | G | 12 | 0 | 0 |
| 17132516 | 17132630 | G | A | 10 | 1 | 125 |
| 17132526 | 17132640 | C | T | 22 | 1 | 57 |
| 17132548 | 17132662 | T | A | 45 | 1 | 28 |
| 17132593 | 17132707 | G | A | 8 | 0 | 0 |
| 17132601 | 17132715 | C | T | 19 | 0 | 0 |
| 17132620 | 17132734 | C | T | 2 | 0 | 0 |
| 17132622 | 17132736 | T | C | 34 | 0 | 0 |
| 17132656 | 17132770 | T | C | 21 | 0 | 0 |
| 17132677 | 17132791 | G | A | 24 | 0 | 0 |
| 17132701 | 17132815 | C | A | 11 | 0 | 0 |
| 17132712 | 17132826 | C | A | 163 | 2 | 15 |
| 17132875 | 17132989 | G | C | 15 | 0 | 0 |
| 17132890 | 17133004 | A | G | 15 | 0 | 0 |
| 17132905 | 17133019 | A | G | 4 | 0 | 0 |
| 17132909 | 17133023 | C | T | 18 | 0 | 0 |
| 17132927 | 17133041 | C | T | 101 | 2 | 25 |
| 17133028 | 17133142 | T | C | 49 | 0 | 0 |
| 17133077 | 17133191 | A | G | 2 | 0 | 0 |
| 17133079 | 17133193 | C | T | 52 | 1 | 24 |
| 17133131 | 17133245 | G | C | 20 | 0 | 0 |
| 17133151 | 17133265 | T | C | 9 | 0 | 0 |
| 17133160 | 17133274 | C | G | 10 | 0 | 0 |
| 17133170 | 17133284 | G | C | 2 | 0 | 0 |
| 17133172 | 17133286 | C | A | 3 | 0 | 0 |
| 17133175 | 17133289 | A | C | 6 | 0 | 0 |
| 17133181 | 17133295 | T | A | 69 | 0 | 0 |
| 17133250 | 17133364 | C | T | 31 | 0 | 0 |
| 17133281 | 17133395 | C | A | 12 | 0 | 0 |
| 17133293 | 17133407 | A | G | 16 | 0 | 0 |
| - | 17133423 | - | GTTTCTTTATTTCAACTTTATTTCAA | 44 | 0 | 0 |
| - | 17133467 | - | CGTA | 11 | 0 | 0 |
| 17133334 | 17133478 | A | G | 10 | 0 | 0 |
| 17133344 | 17133488 | A | T | 7 | 0 | 0 |
| - | 17133495 | - | AT | 5 | 0 | 0 |
| 17133354 | 17133500 | C | A | 14 | 0 | 0 |
| 17133368 | 17133514 | TTATCTCAAAACTATGCATCACCT | - | 28 | 0 | 0 |
| - | 17133542 | - | CTCGAAACTATGTAGTTTGTAATTATGTC | 32 | 0 | 0 |
| - | 17133574 | - | AATAATTCTAACTTTATTTC | 45 | 0 | 0 |
| 17133424 | 17133619 | T | G | 8 | 0 | 0 |
| 17133432 | 17133627 | T | - | 3 | 0 | 0 |
| 17133435 | 17133630 | C | A | 9 | 0 | 0 |
| 17133444 | 17133639 | T | C | 37 | 0 | 0 |
| 17133481 | 17133676 | C | T | 1 | 0 | 0 |
| 17133482 | 17133677 | T | A | 39 | 0 | 0 |
| 17133521 | 17133716 | C | A | 23 | 0 | 0 |
| 17133544 | 17133739 | G | T | 80 | 0 | 0 |
| 17133624 | 17133819 | G | T | 37 | 0 | 0 |
| 17133661 | 17133856 | C | A | 31 | 0 | 0 |
| 17133692 | 17133887 | G | A | 3 | 0 | 0 |
| 17133695 | 17133890 | A | G | 2 | 0 | 0 |
| 17133697 | 17133892 | C | T | 1 | 0 | 0 |
| 17133698 | 17133893 | A | T | 2 | 0 | 0 |
| 17133700 | 17133895 | CTCTATCAC | - | 1032 | 15 | 18 |
| 17134732 | 17134927 | C | T | 714 | 16 | 28 |
| 17135446 | 17135641 | A | G | 5 | 1 | 250 |
| 17135451 | 17135646 | C | A | 34 | 0 | 0 |
| 17135485 | 17135680 | T | - | 2 | 0 | 0 |
| 17135487 | 17135682 | T | G | 29 | 0 | 0 |
| 17135516 | 17135711 | A | G | 24 | 0 | 0 |
| 17135540 | 17135735 | T | G | 67 | 0 | 0 |
| 17135607 | 17135802 | A | T | 97 | 0 | 0 |
| 17135704 | 17135899 | T | C | 7 | 0 | 0 |
| 17135711 | 17135906 | A | C | 2 | 0 | 0 |
| 17135713 | 17135908 | T | A | - | 0 | 0 |
