# Supplementary material for: Interhomolog polymorphism shapes meiotic crossover within the Arabidopsis RAC1 and RPP13 disease resistance genes
Source: PLoS Genet. 2018 Dec 13;14(12):e1007843. doi: 10.1371/journal.pgen.1007843 (PMC6307820; doi:10.1371/journal.pgen.1007843)
Supplement: S7 Table — Recombination rate (cM/Mb) was calculated by dividing genetic distance (cM) by panmolecule physical length. A chi-square test using a 2×2 contingency table was used to test for a significant difference between the genotypes. (DOCX) [file pgen.1007843.s012.docx]

**S7 Table. Recombination rate calculated via pollen-typing across the *RAC1* disease resistance gene in Col×Ler and Col×Mh.**

|  | Col×Ler | Col×Mh |
| --- | --- | --- |
| Parentals/μl | 1,169.3 | 4,655.0 |
| Crossovers/μl | 0.86 | 3.00 |
| cM | 0.074 | 0.064 |
| cM S.D. | 0.012 | 0.008 |
| bp | 9,482 | 9,465 |
| cM/Mb | 7.80 | 6.76 |
| *P* value | n.d. | 0.309 |
