# Supplementary material for: Interhomolog polymorphism shapes meiotic crossover within the Arabidopsis RAC1 and RPP13 disease resistance genes
Source: PLoS Genet. 2018 Dec 13;14(12):e1007843. doi: 10.1371/journal.pgen.1007843 (PMC6307820; doi:10.1371/journal.pgen.1007843)
Supplement: S9 Table — Crossover frequency (cM/Mb) was calculated using Col×Ler F1 titration data genetic distance (0.074 cM), and interval lengths according to the panmolecule. (DOCX) [file pgen.1007843.s014.docx]

**S9 Table. Crossover distributions across the *RAC1* *R* gene hotspot in Col×Wl F_1_ analysed via pollen-typing.**

| TAIR coordinate | Pancoordinate | Col | Wl | Interval length (bp) | Crossovers | cM/Mb |
| --- | --- | --- | --- | --- | --- | --- |
| 11288165 | 11288165 | A | T | 16 | 0 | 0 |
| 11288181 | 11288181 | A | G | 11 | 0 | 0 |
| 11288192 | 11288192 | T | A | 7 | 0 | 0 |
| 11288199 | 11288199 | T | C | 9 | 0 | 0 |
| - | 11288208 | - | CTCTCTACATTACCATCTTCAG | 55 | 0 | 0 |
| 11288241 | 11288263 | C | T | 1 | 0 | 0 |
| 11288242 | 11288264 | C | T | 9 | 0 | 0 |
| 11288251 | 11288273 | A | - | 1 | 0 | 0 |
| 11288252 | 11288274 | A | G | 12 | 0 | 0 |
| - | 11288286 | - | TT | 69 | 1 | 12 |
| 11288331 | 11288355 | C | A | 127 | 1 | 6 |
| 11288458 | 11288482 | A | T | 108 | 0 | 0 |
| 11288566 | 11288590 | A | C | 19 | 0 | 0 |
| 11288585 | 11288609 | C | A | 25 | 0 | 0 |
| 11288610 | 11288634 | C | A | 52 | 0 | 0 |
| 11288662 | 11288686 | C | T | 84 | 1 | 10 |
| 11288746 | 11288770 | C | T | 54 | 0 | 0 |
| 11288800 | 11288824 | GACTGTGA | - | 72 | 0 | 0 |
| 11288872 | 11288896 | A | G | 26 | 0 | 0 |
| 11288898 | 11288922 | G | T | 33 | 1 | 24 |
| 11288931 | 11288955 | C | G | 8 | 0 | 0 |
| 11288939 | 11288963 | T | C | 1 | 0 | 0 |
| 11288940 | 11288964 | G | A | 9 | 0 | 0 |
| 11288949 | 11288973 | G | T | 79 | 0 | 0 |
| 11289028 | 11289052 | T | G | 3 | 0 | 0 |
| 11289031 | 11289055 | A | G | 66 | 0 | 0 |
| 11289097 | 11289121 | T | C | 161 | 3 | 15 |
| 11289258 | 11289282 | A | C | 38 | 1 | 21 |
| 11289296 | 11289320 | G | T | 150 | 0 | 0 |
| 11289446 | 11289470 | T | A | 5 | 0 | 0 |
| 11289451 | 11289475 | A | G | 28 | 0 | 0 |
| 11289479 | 11289503 | A | G | 85 | 0 | 0 |
| 11289564 | 11289588 | T | C | 9 | 0 | 0 |
| 11289573 | 11289597 | T | C | 1 | 0 | 0 |
| 11289574 | 11289598 | G | T | 29 | 1 | 28 |
| 11289603 | 11289627 | G | T | 69 | 0 | 0 |
| 11289672 | 11289696 | C | T | 3 | 0 | 0 |
| 11289675 | 11289699 | T | A | 1 | 0 | 0 |
| 11289676 | 11289700 | A | C | 1 | 0 | 0 |
| 11289677 | 11289701 | T | G | 2 | 0 | 0 |
| 11289679 | 11289703 | A | T | 2 | 0 | 0 |
| 11289681 | 11289705 | A | G | 1 | 0 | 0 |
| 11289682 | 11289706 | G | A | 2 | 0 | 0 |
| 11289684 | 11289708 | A | T | 2 | 0 | 0 |
| 11289686 | 11289710 | T | C | 4 | 0 | 0 |
| 11289690 | 11289714 | C | T | 2 | 0 | 0 |
| 11289692 | 11289716 | C | T | 1 | 0 | 0 |
| 11289693 | 11289717 | C | G | 2 | 0 | 0 |
| 11289695 | 11289719 | T | C | 1 | 0 | 0 |
| 11289696 | 11289720 | A | T | 1 | 0 | 0 |
| 11289697 | 11289721 | A | T | 2 | 0 | 0 |
| 11289699 | 11289723 | A | T | 1 | 0 | 0 |
| 11289700 | 11289724 | T | C | 1 | 0 | 0 |
| 11289701 | 11289725 | C | G | 1 | 0 | 0 |
| - | 11289726 | - | GAA | 5 | 0 | 0 |
| 11289704 | 11289731 | T | A | 1 | 0 | 0 |
| 11289705 | 11289732 | C | G | 2 | 0 | 0 |
| 11289707 | 11289734 | T | A | 1 | 0 | 0 |
| 11289708 | 11289735 | T | G | 3 | 0 | 0 |
| 11289711 | 11289738 | T | A | 4 | 0 | 0 |
| 11289715 | 11289742 | T | G | 2 | 0 | 0 |
| 11289717 | 11289744 | C | A | 6 | 0 | 0 |
| 11289723 | 11289750 | A | C | 2 | 0 | 0 |
| 11289725 | 11289752 | A | T | 1 | 0 | 0 |
| 11289726 | 11289753 | T | C | 1 | 0 | 0 |
| - | 11289754 | - | CGA | 4 | 0 | 0 |
| 11289728 | 11289758 | T | C | 3 | 0 | 0 |
| 11289731 | 11289761 | T | A | 1 | 0 | 0 |
| 11289732 | 11289762 | A | C | 3 | 0 | 0 |
| 11289735 | 11289765 | C | G | 5 | 0 | 0 |
| 11289740 | 11289770 | T | G | 29 | 0 | 0 |
| 11289769 | 11289799 | T | C | 3 | 0 | 0 |
| 11289772 | 11289802 | T | C | 5 | 0 | 0 |
| 11289777 | 11289807 | C | T | 1 | 0 | 0 |
| 11289778 | 11289808 | G | T | 2 | 0 | 0 |
| 11289780 | 11289810 | G | T | 1 | 0 | 0 |
| 11289781 | 11289811 | C | A | 2 | 0 | 0 |
| 11289783 | 11289813 | C | T | 1 | 0 | 0 |
| 11289784 | 11289814 | ACTG | - | 6 | 0 | 0 |
| 11289790 | 11289820 | C | T | 4 | 0 | 0 |
| 11289794 | 11289824 | T | A | 1 | 0 | 0 |
| 11289795 | 11289825 | C | G | 5 | 0 | 0 |
| 11289800 | 11289830 | T | A | 1 | 0 | 0 |
| 11289801 | 11289831 | T | C | 2 | 0 | 0 |
| 11289803 | 11289833 | G | T | 2 | 0 | 0 |
| 11289805 | 11289835 | T | A | 1 | 0 | 0 |
| 11289806 | 11289836 | A | G | 1 | 0 | 0 |
| 11289807 | 11289837 | C | T | 1 | 0 | 0 |
| 11289808 | 11289838 | C | T | 2 | 0 | 0 |
| 11289810 | 11289840 | A | C | 1 | 0 | 0 |
| 11289811 | 11289841 | T | C | 1 | 0 | 0 |
| 11289812 | 11289842 | G | A | 5 | 0 | 0 |
| - | 11289847 | - | C | 3 | 0 | 0 |
| 11289819 | 11289850 | T | C | 42 | 0 | 0 |
| 11289861 | 11289892 | T | G | 79 | 0 | 0 |
| 11289940 | 11289971 | G | A | 43 | 0 | 0 |
| 11289983 | 11290014 | G | A | 85 | 0 | 0 |
| 11290068 | 11290099 | C | T | 45 | 1 | 18 |
| 11290113 | 11290144 | T | C | 1 | 0 | 0 |
| 11290114 | 11290145 | A | C | 74 | 0 | 0 |
| 11290188 | 11290219 | T | G | 75 | 3 | 32 |
| 11290263 | 11290294 | C | T | 58 | 0 | 0 |
| 11290321 | 11290352 | G | A | 7 | 0 | 0 |
| 11290328 | 11290359 | C | T | 1 | 0 | 0 |
| 11290329 | 11290360 | T | C | 5 | 0 | 0 |
| 11290334 | 11290365 | C | G | 7 | 0 | 0 |
| 11290341 | 11290372 | A | C | 4 | 0 | 0 |
| 11290345 | 11290376 | A | G | 50 | 1 | 16 |
| 11290395 | 11290426 | T | A | 8 | 0 | 0 |
| 11290403 | 11290434 | A | C | 4 | 0 | 0 |
| 11290407 | 11290438 | A | G | 1 | 0 | 0 |
| 11290408 | 11290439 | G | C | 35 | 0 | 0 |
| 11290443 | 11290474 | T | G | 19 | 0 | 0 |
| 11290462 | 11290493 | G | C | 2 | 0 | 0 |
| 11290464 | 11290495 | G | T | 3 | 0 | 0 |
| 11290467 | 11290498 | T | A | 1 | 0 | 0 |
| 11290468 | 11290499 | C | A | 1 | 0 | 0 |
| 11290469 | 11290500 | T | C | 1 | 0 | 0 |
| 11290470 | 11290501 | C | A | 5 | 0 | 0 |
| 11290475 | 11290506 | T | C | 49 | 0 | 0 |
| 11290524 | 11290555 | G | A | 3 | 0 | 0 |
| 11290527 | 11290558 | G | T | 125 | 1 | 6 |
| 11290652 | 11290683 | G | A | 2 | 0 | 0 |
| 11290654 | 11290685 | T | C | 7 | 0 | 0 |
| - | 11290692 | - | T | 2 | 0 | 0 |
| 11290662 | 11290694 | G | A | 25 | 1 | 32 |
| 11290687 | 11290719 | A | C | 26 | 0 | 0 |
| 11290713 | 11290745 | A | - | 69 | 0 | 0 |
| 11290782 | 11290814 | G | A | 10 | 0 | 0 |
| 11290792 | 11290824 | C | T | 29 | 0 | 0 |
| 11290821 | 11290853 | G | A | 5 | 0 | 0 |
| 11290826 | 11290858 | A | T | 11 | 0 | 0 |
| 11290837 | 11290869 | A | G | 35 | 1 | 23 |
| - | 11290904 | - | T | 6 | 0 | 0 |
| 11290877 | 11290910 | G | C | 15 | 0 | 0 |
| 11290892 | 11290925 | AT | - | 7 | 0 | 0 |
| 11290899 | 11290932 | T | A | 5 | 0 | 0 |
| 11290904 | 11290937 | T | A | 62 | 0 | 0 |
| 11290966 | 11290999 | T | A | 13 | 0 | 0 |
| 11290979 | 11291012 | T | A | 31 | 0 | 0 |
| 11291010 | 11291043 | C | T | 2 | 0 | 0 |
| - | 11291045 | - | ACAAGTGTACAAAGTTT | 37 | 0 | 0 |
| 11291032 | 11291082 | T | C | 4 | 0 | 0 |
| 11291036 | 11291086 | A | T | 20 | 0 | 0 |
| 11291056 | 11291106 | G | A | 26 | 0 | 0 |
| 11291082 | 11291132 | G | T | 21 | 0 | 0 |
| 11291103 | 11291153 | T | C | 25 | 0 | 0 |
| 11291128 | 11291178 | C | T | 13 | 0 | 0 |
| 11291141 | 11291191 | T | G | 7 | 0 | 0 |
| 11291148 | 11291198 | T | G | 13 | 0 | 0 |
| 11291161 | 11291211 | C | T | 1 | 0 | 0 |
| 11291162 | 11291212 | G | C | 41 | 0 | 0 |
| 11291203 | 11291253 | T | A | 5 | 0 | 0 |
| 11291208 | 11291258 | T | C | 64 | 0 | 0 |
| 11291272 | 11291322 | T | G | 60 | 1 | 13 |
| 11291332 | 11291382 | T | A | 1 | 0 | 0 |
| 11291333 | 11291383 | T | G | 2 | 0 | 0 |
| 11291335 | 11291385 | G | A | 43 | 0 | 0 |
| 11291378 | 11291428 | C | T | 20 | 0 | 0 |
| 11291398 | 11291448 | C | T | 8 | 0 | 0 |
| 11291406 | 11291456 | T | G | 1 | 0 | 0 |
| 11291407 | 11291457 | T | A | 55 | 0 | 0 |
| 11291462 | 11291512 | G | C | 4 | 0 | 0 |
| 11291466 | 11291516 | G | A | 6 | 0 | 0 |
| 11291472 | 11291522 | C | A | 3 | 0 | 0 |
| 11291475 | 11291525 | T | C | 23 | 0 | 0 |
| 11291498 | 11291548 | T | C | 42 | 2 | 38 |
| 11291540 | 11291590 | A | C | 16 | 0 | 0 |
| 11291556 | 11291606 | T | C | 12 | 0 | 0 |
| 11291568 | 11291618 | G | A | 12 | 0 | 0 |
| 11291580 | 11291630 | C | A | 6 | 0 | 0 |
| 11291586 | 11291636 | A | - | 4 | 0 | 0 |
| 11291590 | 11291640 | A | - | 4 | 0 | 0 |
| 11291594 | 11291644 | A | - | 24 | 0 | 0 |
| 11291618 | 11291668 | G | A | 33 | 2 | 49 |
| 11291651 | 11291701 | T | C | 70 | 1 | 11 |
| 11291721 | 11291771 | A | G | 80 | 1 | 10 |
| - | 11291851 | - | CAT | 70 | 2 | 23 |
| 11291868 | 11291921 | G | C | 11 | 0 | 0 |
| 11291879 | 11291932 | A | G | 83 | 6 | 58 |
| 11291962 | 11292015 | C | T | 229 | 3 | 11 |
| 11292191 | 11292244 | G | A | 23 | 2 | 70 |
| 11292214 | 11292267 | C | T | 537 | 11 | 16 |
| 11292751 | 11292804 | G | A | 12 | 0 | 0 |
| 11292763 | 11292816 | A | G | 28 | 1 | 29 |
| 11292791 | 11292844 | T | C | 6 | 0 | 0 |
| 11292797 | 11292850 | C | G | 384 | 9 | 19 |
| 11293181 | 11293234 | A | G | 45 | 0 | 0 |
| 11293226 | 11293279 | A | T | 2 | 0 | 0 |
| - | 11293281 | - | TAA | 41 | 1 | 20 |
| 11293266 | 11293322 | C | A | 283 | 7 | 20 |
| 11293549 | 11293605 | C | T | 140 | 0 | 0 |
| 11293689 | 11293745 | C | A | 109 | 3 | 22 |
| 11293798 | 11293854 | A | C | 134 | 3 | 18 |
| 11293932 | 11293988 | C | - | 48 | 1 | 17 |
| 11293980 | 11294036 | G | T | 9 | 0 | 0 |
| 11293989 | 11294045 | T | C | 1 | 0 | 0 |
| 11293990 | 11294046 | T | G | 17 | 0 | 0 |
| 11294007 | 11294063 | A | C | 36 | 1 | 22 |
| 11294043 | 11294099 | T | A | 15 | 0 | 0 |
| 11294058 | 11294114 | T | C | 36 | 0 | 0 |
| 11294094 | 11294150 | C | A | 14 | 0 | 0 |
| 11294108 | 11294164 | C | T | 28 | 0 | 0 |
| - | 11294192 | - | AA | 5 | 0 | 0 |
| 11294139 | 11294197 | C | T | 2 | 0 | 0 |
| 11294141 | 11294199 | C | T | 37 | 0 | 0 |
| 11294178 | 11294236 | A | C | 6 | 0 | 0 |
| 11294184 | 11294242 | G | C | 26 | 0 | 0 |
| 11294210 | 11294268 | A | C | 7 | 0 | 0 |
| 11294217 | 11294275 | T | A | 46 | 0 | 0 |
| - | 11294321 | - | T | 73 | 0 | 0 |
| 11294335 | 11294394 | G | A | 1 | 0 | 0 |
| 11294336 | 11294395 | T | A | 13 | 0 | 0 |
| 11294349 | 11294408 | A | T | 2 | 0 | 0 |
| 11294351 | 11294410 | T | G | 8 | 0 | 0 |
| 11294359 | 11294418 | A | T | 8 | 0 | 0 |
| 11294367 | 11294426 | A | T | 1 | 0 | 0 |
| 11294368 | 11294427 | G | A | 16 | 0 | 0 |
| 11294384 | 11294443 | A | T | 3 | 0 | 0 |
| 11294387 | 11294446 | C | A | 5 | 0 | 0 |
| 11294392 | 11294451 | C | T | 4 | 0 | 0 |
| 11294396 | 11294455 | A | C | 48 | 1 | 17 |
| 11294444 | 11294503 | G | C | 25 | 0 | 0 |
| 11294469 | 11294528 | A | G | 21 | 0 | 0 |
| 11294490 | 11294549 | A | C | 9 | 0 | 0 |
| 11294499 | 11294558 | A | C | 1 | 0 | 0 |
| 11294500 | 11294559 | T | A | 7 | 0 | 0 |
| - | 11294566 | - | T | 19 | 0 | 0 |
| 11294525 | 11294585 | C | A | 4 | 0 | 0 |
| 11294529 | 11294589 | T | A | 9 | 0 | 0 |
| 11294538 | 11294598 | A | G | 4 | 0 | 0 |
| - | 11294602 | - | T | 9 | 0 | 0 |
| 11294550 | 11294611 | A | T | 5 | 0 | 0 |
| 11294555 | 11294616 | T | G | 16 | 0 | 0 |
| 11294571 | 11294632 | T | C | 6 | 0 | 0 |
| 11294577 | 11294638 | GGTGAGAGCTTAAAAACCCACAAAAA | - | 52 | 0 | 0 |
| 11294629 | 11294690 | T | - | 9 | 0 | 0 |
| 11294638 | 11294699 | T | G | 15 | 0 | 0 |
| 11294653 | 11294714 | A | C | 7 | 0 | 0 |
| 11294660 | 11294721 | A | T | 8 | 0 | 0 |
| 11294668 | 11294729 | G | A | 2 | 0 | 0 |
| 11294670 | 11294731 | A | - | 1 | 0 | 0 |
| 11294671 | 11294732 | C | T | 11 | 0 | 0 |
| 11294682 | 11294743 | C | T | 6 | 0 | 0 |
| 11294688 | 11294749 | T | A | 6 | 0 | 0 |
| 11294694 | 11294755 | G | C | 25 | 0 | 0 |
| 11294719 | 11294780 | GT | - | 23 | 0 | 0 |
| 11294742 | 11294803 | C | A | 17 | 0 | 0 |
| 11294759 | 11294820 | A | T | 15 | 0 | 0 |
| - | 11294835 | - | GC | 5 | 0 | 0 |
| 11294777 | 11294840 | A | - | 26 | 0 | 0 |
| - | 11294866 | - | A | 11 | 0 | 0 |
| 11294813 | 11294877 | T | C | 23 | 0 | 0 |
| - | 11294900 | - | TGT | 41 | 0 | 0 |
| 11294874 | 11294941 | C | A | 31 | 0 | 0 |
| 11294905 | 11294972 | A | G | 11 | 0 | 0 |
| 11294916 | 11294983 | A | C | 19 | 0 | 0 |
| 11294935 | 11295002 | G | A | 2 | 0 | 0 |
| 11294937 | 11295004 | T | A | 23 | 0 | 0 |
| 11294960 | 11295027 | C | A | 20 | 0 | 0 |
| 11294980 | 11295047 | T | A | 11 | 0 | 0 |
| 11294991 | 11295058 | A | G | 47 | 1 | 17 |
| 11295038 | 11295105 | G | T | 53 | 0 | 0 |
| 11295091 | 11295158 | C | A | 35 | 1 | 23 |
| 11295126 | 11295193 | C | A | 25 | 0 | 0 |
| 11295151 | 11295218 | G | A | 17 | 0 | 0 |
| 11295168 | 11295235 | A | T | 4 | 0 | 0 |
| 11295172 | 11295239 | T | C | 1 | 0 | 0 |
| 11295173 | 11295240 | T | C | 9 | 0 | 0 |
| 11295182 | 11295249 | G | A | 5 | 0 | 0 |
| 11295187 | 11295254 | C | T | 6 | 0 | 0 |
| 11295193 | 11295260 | C | A | 2 | 0 | 0 |
| 11295195 | 11295262 | C | G | 22 | 0 | 0 |
| 11295217 | 11295284 | T | C | 17 | 0 | 0 |
| 11295234 | 11295301 | A | T | 28 | 0 | 0 |
| 11295262 | 11295329 | C | A | 11 | 0 | 0 |
| 11295273 | 11295340 | T | G | 16 | 0 | 0 |
| 11295289 | 11295356 | G | A | 1 | 0 | 0 |
| 11295290 | 11295357 | G | A | 9 | 0 | 0 |
| 11295299 | 11295366 | C | T | 19 | 0 | 0 |
| 11295318 | 11295385 | A | T | 35 | 0 | 0 |
| 11295353 | 11295420 | TC | - | 11 | 0 | 0 |
| 11295364 | 11295431 | C | T | 2 | 0 | 0 |
| - | 11295433 | - | T | 10 | 0 | 0 |
| 11295375 | 11295443 | A | G | 3 | 0 | 0 |
| 11295378 | 11295446 | C | G | 1 | 0 | 0 |
| 11295379 | 11295447 | C | T | 9 | 0 | 0 |
| 11295388 | 11295456 | A | T | 2 | 0 | 0 |
| 11295390 | 11295458 | G | A | 16 | 0 | 0 |
| 11295406 | 11295474 | T | A | 13 | 0 | 0 |
| 11295419 | 11295487 | G | A | 24 | 0 | 0 |
| 11295443 | 11295511 | A | T | 36 | 0 | 0 |
| 11295479 | 11295547 | T | A | 17 | 0 | 0 |
| 11295496 | 11295564 | G | T | 28 | 0 | 0 |
| 11295524 | 11295592 | T | A | 17 | 0 | 0 |
| 11295541 | 11295609 | G | - | 3 | 0 | 0 |
| 11295544 | 11295612 | T | C | 15 | 0 | 0 |
| 11295559 | 11295627 | T | A | 9 | 0 | 0 |
| 11295568 | 11295636 | T | A | 41 | 0 | 0 |
| 11295609 | 11295677 | A | T | 127 | 1 | 6 |
| 11295736 | 11295804 | C | G | 154 | 3 | 16 |
| 11295890 | 11295958 | T | G | 31 | 0 | 0 |
| 11295921 | 11295989 | G | A | 5 | 0 | 0 |
| - | 11295994 | - | TAG | 30 | 0 | 0 |
| 11295953 | 11296024 | C | A | 41 | 0 | 0 |
| 11295994 | 11296065 | A | G | 79 | 0 | 0 |
| 11296073 | 11296144 | C | T | 57 | 2 | 28 |
| 11296130 | 11296201 | C | T | 96 | 0 | 0 |
| 11296226 | 11296297 | T | G | 24 | 0 | 0 |
| 11296250 | 11296321 | T | C | 19 | 1 | 42 |
| 11296269 | 11296340 | TGGA | - | 22 | 0 | 0 |
| 11296291 | 11296362 | A | C | 178 | 4 | 18 |
| 11296469 | 11296540 | T | C | 193 | 0 | 0 |
| 11296662 | 11296733 | A | T | 23 | 0 | 0 |
| 11296685 | 11296756 | T | C | 22 | 0 | 0 |
| 11296707 | 11296778 | C | T | 7 | 0 | 0 |
| 11296714 | 11296785 | A | C | 27 | 0 | 0 |
| 11296741 | 11296812 | T | C | 81 | 0 | 0 |
| 11296822 | 11296893 | C | A | 42 | 0 | 0 |
| 11296864 | 11296935 | T | A | 12 | 0 | 0 |
| 11296876 | 11296947 | T | C | 18 | 0 | 0 |
| 11296894 | 11296965 | TTGACATAAGAAACCTAAGAA | - | 44 | 0 | 0 |
| - | 11297009 | - | AAA | 7 | 0 | 0 |
| 11296942 | 11297016 | G | A | 5 | 0 | 0 |
| 11296947 | 11297021 | C | T | 2 | 0 | 0 |
| 11296949 | 11297023 | T | C | 5 | 0 | 0 |
| 11296954 | 11297028 | G | A | 8 | 0 | 0 |
| 11296962 | 11297036 | A | G | 2 | 0 | 0 |
| 11296964 | 11297038 | C | T | 2 | 0 | 0 |
| 11296966 | 11297040 | T | C | 3 | 0 | 0 |
| 11296969 | 11297043 | C | - | 4 | 0 | 0 |
| 11296973 | 11297047 | A | G | 6 | 0 | 0 |
| 11296979 | 11297053 | C | T | 6 | 0 | 0 |
| 11296985 | 11297059 | C | A | 13 | 0 | 0 |
| 11296998 | 11297072 | C | T | 48 | 4 | 67 |
| 11297046 | 11297120 | G | A | 21 | 0 | 0 |
| 11297067 | 11297141 | T | G | 23 | 0 | 0 |
| 11297090 | 11297164 | T | G | 42 | 0 | 0 |
| 11297132 | 11297206 | T | G | 80 | 0 | 0 |
| 11297212 | 11297286 | C | A | 6 | 0 | 0 |
| 11297218 | 11297292 | A | T | 1 | 0 | 0 |
| 11297219 | 11297293 | A | G | 28 | 0 | 0 |
| 11297247 | 11297321 | C | A | 41 | 0 | 0 |
| 11297288 | 11297362 | A | T | 8 | 0 | 0 |
| 11297296 | 11297370 | A | G | 68 | 0 | 0 |
| - | 11297438 | - | G | 46 | 0 | 0 |
| 11297409 | 11297484 | A | - | 3 | 0 | 0 |
| 11297412 | 11297487 | G | T | 32 | 0 | 0 |
| 11297444 | 11297519 | A | G | 10 | 0 | 0 |
| 11297454 | 11297529 | T | C | 7 | 0 | 0 |
| 11297461 | 11297536 | T | G | 31 | 0 | 0 |
| 11297492 | 11297567 | A | G | 56 | 0 | 0 |
| 11297548 | 11297623 | G | A | - | 0 | 0 |
