# Supplementary material for: Interhomolog polymorphism shapes meiotic crossover within the Arabidopsis RAC1 and RPP13 disease resistance genes
Source: PLoS Genet. 2018 Dec 13;14(12):e1007843. doi: 10.1371/journal.pgen.1007843 (PMC6307820; doi:10.1371/journal.pgen.1007843)
Supplement: S10 Table — Crossover frequency (cM/Mb) was calculated using Col×Mh F1 titration data genetic distance (0.064 cM), and interval lengths according to the panmolecule. (DOCX) [file pgen.1007843.s015.docx]

**S10 Table. Crossover distributions within the *RAC1* amplicon from Col×Mh F_1_ analysed via pollen-typing.**

| TAIR coordinate | Pan  coordinate | Col | Mh | Interval length (bp) | Crossovers | cM/Mb |
| --- | --- | --- | --- | --- | --- | --- |
| 11288165 | 11288165 | A | T | 16 | 0 | 0 |
| 11288181 | 11288181 | A | G | 11 | 0 | 0 |
| 11288192 | 11288192 | T | A | 7 | 0 | 0 |
| 11288199 | 11288199 | T | C | 8 | 0 | 0 |
| 11288207 | 11288207 | T | G | 35 | 0 | 0 |
| 11288242 | 11288242 | C | A | 5 | 0 | 0 |
| 11288247 | 11288247 | G | C | 4 | 0 | 0 |
| 11288251 | 11288251 | A | - | 1 | 0 | 0 |
| 11288252 | 11288252 | A | G | 66 | 0 | 0 |
| 11288318 | 11288318 | T | A | 13 | 0 | 0 |
| 11288331 | 11288331 | C | A | 235 | 0 | 0 |
| 11288566 | 11288566 | A | C | 19 | 0 | 0 |
| 11288585 | 11288585 | C | A | 16 | 0 | 0 |
| 11288601 | 11288601 | T | - | 175 | 1 | 3 |
| 11288776 | 11288776 | T | A | 164 | 0 | 0 |
| 11288940 | 11288940 | G | A | 157 | 2 | 7 |
| 11289097 | 11289097 | T | C | 87 | 0 | 0 |
| 11289184 | 11289184 | CCTCTAAGTGTCGTATTTACCCCATCCGTTTAGTTTGGACGTAGACACCCTAATATTACTTATATGAAGTGATATAGCTGCAAAAGAATCTAGTTGTCAATAATGCAAAAAGAATTGAGAGATGAATAGTCATTATGTTACAATTATTGCTTAATGCTTACCCGCATGCGCTTCGTGCTTCTCTCAGTTTCTACATTGATATCTCCAG | - | 209 | 0 | 0 |
| 11289393 | 11289393 | G | A | 53 | 0 | 0 |
| 11289446 | 11289446 | T | A | 5 | 0 | 0 |
| 11289451 | 11289451 | A | G | 43 | 0 | 0 |
| 11289494 | 11289494 | AAT | - | 66 | 0 | 0 |
| 11289560 | 11289560 | T | C | 4 | 0 | 0 |
| 11289564 | 11289564 | T | C | 9 | 0 | 0 |
| 11289573 | 11289573 | T | C | 1 | 0 | 0 |
| 11289574 | 11289574 | G | C | 2 | 0 | 0 |
| 11289576 | 11289576 | C | T | 108 | 0 | 0 |
| 11289684 | 11289684 | A | C | 17 | 0 | 0 |
| 11289701 | 11289701 | C | A | 4 | 0 | 0 |
| 11289705 | 11289705 | C | A | 6 | 0 | 0 |
| 11289711 | 11289711 | T | C | 70 | 1 | 7 |
| 11289781 | 11289781 | C | A | 7 | 0 | 0 |
| 11289788 | 11289788 | GCTCCT | - | 14 | 0 | 0 |
| 11289802 | 11289802 | A | C | 1 | 0 | 0 |
| 11289803 | 11289803 | G | T | 58 | 0 | 0 |
| 11289861 | 11289861 | T | G | 10 | 0 | 0 |
| 11289871 | 11289871 | C | T | 59 | 0 | 0 |
| 11289930 | 11289930 | T | G | 13 | 0 | 0 |
| 11289943 | 11289943 | G | C | 24 | 0 | 0 |
| 11289967 | 11289967 | TGA | - | 37 | 0 | 0 |
| 11290004 | 11290004 | A | C | 1 | 0 | 0 |
| 11290005 | 11290005 | A | T | 5 | 0 | 0 |
| 11290010 | 11290010 | C | T | 1 | 0 | 0 |
| 11290011 | 11290011 | T | C | 1 | 0 | 0 |
| 11290012 | 11290012 | G | A | 4 | 0 | 0 |
| 11290016 | 11290016 | A | T | 7 | 0 | 0 |
| 11290023 | 11290023 | T | G | 45 | 0 | 0 |
| 11290068 | 11290068 | C | T | 56 | 1 | 9 |
| 11290124 | 11290124 | C | T | 85 | 1 | 6 |
| 11290209 | 11290209 | C | T | 119 | 3 | 13 |
| 11290328 | 11290328 | C | T | 17 | 0 | 0 |
| 11290345 | 11290345 | A | G | 52 | 1 | 10 |
| 11290397 | 11290397 | A | G | 5 | 0 | 0 |
| 11290402 | 11290402 | G | C | 1 | 0 | 0 |
| 11290403 | 11290403 | A | C | 5 | 0 | 0 |
| 11290408 | 11290408 | G | C | 2 | 0 | 0 |
| 11290410 | 11290410 | C | T | 33 | 0 | 0 |
| 11290443 | 11290443 | T | G | 19 | 0 | 0 |
| 11290462 | 11290462 | G | C | 2 | 0 | 0 |
| 11290464 | 11290464 | G | T | 3 | 0 | 0 |
| 11290467 | 11290467 | T | C | 1 | 0 | 0 |
| 11290468 | 11290468 | C | A | 1 | 0 | 0 |
| 11290469 | 11290469 | T | C | 1 | 0 | 0 |
| 11290470 | 11290470 | C | G | 5 | 0 | 0 |
| 11290475 | 11290475 | T | G | 1 | 0 | 0 |
| 11290476 | 11290476 | G | T | 4 | 0 | 0 |
| 11290480 | 11290480 | G | T | 108 | 0 | 0 |
| 11290588 | 11290588 | T | - | 64 | 0 | 0 |
| 11290652 | 11290652 | G | A | 2 | 0 | 0 |
| 11290654 | 11290654 | T | C | 8 | 0 | 0 |
| 11290662 | 11290662 | G | T | 2 | 0 | 0 |
| - | 11290664 | - | ATGTATTATGGGTAAATCTAAACAAGAATAAA | 55 | 0 | 0 |
| 11290687 | 11290719 | A | C | 26 | 0 | 0 |
| 11290713 | 11290745 | A | - | 27 | 0 | 0 |
| 11290740 | 11290772 | C | T | 42 | 0 | 0 |
| 11290782 | 11290814 | G | A | 59 | 1 | 9 |
| 11290841 | 11290873 | C | T | 36 | 0 | 0 |
| 11290877 | 11290909 | G | C | 6 | 0 | 0 |
| 11290883 | 11290915 | T | A | 6 | 0 | 0 |
| 11290889 | 11290921 | TTA | - | 10 | 0 | 0 |
| 11290899 | 11290931 | T | A | 5 | 0 | 0 |
| 11290904 | 11290936 | T | A | 62 | 0 | 0 |
| 11290966 | 11290998 | T | A | 13 | 0 | 0 |
| 11290979 | 11291011 | T | A | 31 | 0 | 0 |
| 11291010 | 11291042 | C | T | 1 | 0 | 0 |
| - | 11291043 | - | ACAAGTGTACAAAGTTT | 38 | 0 | 0 |
| 11291032 | 11291081 | T | C | 4 | 0 | 0 |
| 11291036 | 11291085 | A | T | 20 | 0 | 0 |
| 11291056 | 11291105 | G | A | 26 | 0 | 0 |
| 11291082 | 11291131 | G | T | 21 | 0 | 0 |
| 11291103 | 11291152 | T | C | 38 | 1 | 14 |
| 11291141 | 11291190 | T | G | 20 | 0 | 0 |
| 11291161 | 11291210 | C | G | 42 | 0 | 0 |
| 11291203 | 11291252 | T | C | 69 | 0 | 0 |
| 11291272 | 11291321 | T | G | 118 | 1 | 4 |
| 11291390 | 11291439 | C | T | 6 | 0 | 0 |
| 11291396 | 11291445 | C | G | 2 | 0 | 0 |
| 11291398 | 11291447 | C | G | 9 | 0 | 0 |
| 11291407 | 11291456 | T | G | 13 | 0 | 0 |
| 11291420 | 11291469 | C | T | 15 | 1 | 34 |
| 11291435 | 11291484 | T | C | 2 | 0 | 0 |
| 11291437 | 11291486 | C | T | 1 | 0 | 0 |
| 11291438 | 11291487 | A | G | 24 | 0 | 0 |
| 11291462 | 11291511 | G | C | 4 | 0 | 0 |
| 11291466 | 11291515 | G | A | 6 | 0 | 0 |
| 11291472 | 11291521 | C | A | 3 | 0 | 0 |
| 11291475 | 11291524 | T | C | 65 | 2 | 16 |
| 11291540 | 11291589 | A | C | 16 | 0 | 0 |
| 11291556 | 11291605 | T | C | 160 | 2 | 6 |
| 11291716 | 11291765 | T | G | 2 | 0 | 0 |
| 11291718 | 11291767 | T | C | 77 | 1 | 7 |
| 11291795 | 11291844 | C | T | 6 | 0 | 0 |
| - | 11291850 | - | AAT | 70 | 2 | 15 |
| 11291868 | 11291920 | G | C | 11 | 0 | 0 |
| 11291879 | 11291931 | A | G | 23 | 2 | 45 |
| 11291902 | 11291954 | C | A | 94 | 2 | 11 |
| 11291996 | 11292048 | T | C | 65 | 1 | 8 |
| 11292061 | 11292113 | C | G | 178 | 8 | 23 |
| 11292239 | 11292291 | T | A | 140 | 6 | 22 |
| 11292379 | 11292431 | T | C | 89 | 4 | 23 |
| 11292468 | 11292520 | T | C | 46 | 0 | 0 |
| 11292514 | 11292566 | T | C | 44 | 1 | 12 |
| 11292558 | 11292610 | C | T | 26 | 0 | 0 |
| 11292584 | 11292636 | T | G | 21 | 0 | 0 |
| 11292605 | 11292657 | T | C | 10 | 0 | 0 |
| 11292615 | 11292667 | A | G | 148 | 8 | 28 |
| 11292763 | 11292815 | A | G | 123 | 2 | 8 |
| 11292886 | 11292938 | A | T | 97 | 4 | 21 |
| 11292983 | 11293035 | G | T | 6 | 0 | 0 |
| 11292989 | 11293041 | C | T | 26 | 0 | 0 |
| 11293015 | 11293067 | T | C | 53 | 2 | 19 |
| 11293068 | 11293120 | A | G | 632 | 30 | 24 |
| - | 11293752 | - | AG | 141 | 2 | 7 |
| 11293837 | 11293893 | A | G | 5 | 0 | 0 |
| 11293842 | 11293898 | T | C | 57 | 0 | 0 |
| 11293899 | 11293955 | G | C | 63 | 1 | 8 |
| 11293962 | 11294018 | T | A | 10 | 0 | 0 |
| 11293972 | 11294028 | G | A | 1 | 0 | 0 |
| 11293973 | 11294029 | T | A | 74 | 0 | 0 |
| 11294047 | 11294103 | T | C | 6 | 0 | 0 |
| - | 11294109 | - | ATTAT | 39 | 1 | 13 |
| - | 11294148 | - | A | 3 | 0 | 0 |
| 11294089 | 11294151 | G | A | 8 | 0 | 0 |
| 11294097 | 11294159 | C | T | 28 | 0 | 0 |
| - | 11294187 | - | AC | 7 | 0 | 0 |
| 11294130 | 11294194 | C | T | 2 | 0 | 0 |
| 11294132 | 11294196 | T | C | 3 | 0 | 0 |
| 11294135 | 11294199 | A | T | 20 | 1 | 26 |
| 11294155 | 11294219 | C | A | 1 | 0 | 0 |
| - | 11294220 | - | A | 3 | 0 | 0 |
| 11294158 | 11294223 | G | C | 10 | 0 | 0 |
| 11294168 | 11294233 | A | C | 4 | 0 | 0 |
| 11294172 | 11294237 | T | G | 4 | 0 | 0 |
| - | 11294241 | - | CAGTA | 23 | 0 | 0 |
| 11294194 | 11294264 | T | A | 13 | 0 | 0 |
| 11294207 | 11294277 | T | A | 8 | 0 | 0 |
| 11294215 | 11294285 | C | A | 10 | 0 | 0 |
| 11294225 | 11294295 | C | T | 51 | 1 | 10 |
| - | 11294346 | A | - | 51 | 0 | 0 |
| 11294326 | 11294397 | T | A | 13 | 0 | 0 |
| 11294339 | 11294410 | A | G | 35 | 0 | 0 |
| 11294374 | 11294445 | A | T | 19 | 0 | 0 |
| 11294393 | 11294464 | C | A | 64 | 0 | 0 |
| 11294457 | 11294528 | A | G | 14 | 0 | 0 |
| 11294471 | 11294542 | T | G | 19 | 0 | 0 |
| 11294490 | 11294561 | T | C | 113 | 4 | 18 |
| 11294603 | 11294674 | G | A | 163 | 0 | 0 |
| - | 11294837 | - | T | 30 | 0 | 0 |
| - | 11294867 | - | A | 10 | 0 | 0 |
| 11294804 | 11294877 | T | C | 368 | 2 | 3 |
| 11295172 | 11295245 | T | C | 22 | 0 | 0 |
| 11295194 | 11295267 | C | G | 83 | 0 | 0 |
| 11295277 | 11295350 | T | C | 5 | 0 | 0 |
| 11295282 | 11295355 | G | A | 87 | 0 | 0 |
| - | 11295442 | - | T | 96 | 0 | 0 |
| 11295464 | 11295538 | C | T | 31 | 1 | 17 |
| 11295495 | 11295569 | G | C | 28 | 1 | 18 |
| 11295523 | 11295597 | T | A | 17 | 0 | 0 |
| 11295540 | 11295614 | G | A | 27 | 0 | 0 |
| 11295567 | 11295641 | T | A | 65 | 0 | 0 |
| 11295632 | 11295706 | A | T | 103 | 3 | 15 |
| 11295735 | 11295809 | C | G | 70 | 2 | 15 |
| - | 11295879 | - | A | 85 | 0 | 0 |
| 11295889 | 11295964 | T | G | 31 | 0 | 0 |
| 11295920 | 11295995 | G | A | 5 | 0 | 0 |
| - | 11296000 | - | AGT | 30 | 1 | 17 |
| 11295952 | 11296030 | C | A | 41 | 0 | 0 |
| 11295993 | 11296071 | A | G | 79 | 0 | 0 |
| 11296072 | 11296150 | C | T | 57 | 0 | 0 |
| 11296129 | 11296207 | C | T | 96 | 2 | 11 |
| 11296225 | 11296303 | T | G | 24 | 0 | 0 |
| 11296249 | 11296327 | T | C | 19 | 0 | 0 |
| 11296268 | 11296346 | TGGA | - | 23 | 0 | 0 |
| 11296291 | 11296369 | A | C | 178 | 2 | 6 |
| 11296469 | 11296547 | T | C | 193 | 2 | 5 |
| 11296662 | 11296740 | A | T | 23 | 0 | 0 |
| 11296685 | 11296763 | T | C | 22 | 0 | 0 |
| 11296707 | 11296785 | C | T | 7 | 0 | 0 |
| 11296714 | 11296792 | A | C | 27 | 0 | 0 |
| 11296741 | 11296819 | T | C | 81 | 1 | 6 |
| 11296822 | 11296900 | C | A | 42 | 0 | 0 |
| 11296864 | 11296942 | T | A | 12 | 0 | 0 |
| 11296876 | 11296954 | T | C | 19 | 0 | 0 |
| 11296895 | 11296973 | TTGACATAAGAAACCTAAGAA | - | 40 | 0 | 0 |
| - | 11297013 | - | AAA | 15 | 0 | 0 |
| 11296947 | 11297028 | C | T | 2 | 0 | 0 |
| 11296949 | 11297030 | T | C | 5 | 0 | 0 |
| 11296954 | 11297035 | G | A | 8 | 0 | 0 |
| 11296962 | 11297043 | A | G | 2 | 0 | 0 |
| 11296964 | 11297045 | C | T | 2 | 0 | 0 |
| 11296966 | 11297047 | T | C | 3 | 0 | 0 |
| 11296969 | 11297050 | C | - | 4 | 0 | 0 |
| 11296973 | 11297054 | A | G | 6 | 0 | 0 |
| 11296979 | 11297060 | C | T | 6 | 0 | 0 |
| 11296985 | 11297066 | C | A | 13 | 0 | 0 |
| 11296998 | 11297079 | C | T | 48 | 1 | 11 |
| 11297046 | 11297127 | G | A | 86 | 0 | 0 |
| 11297132 | 11297213 | T | G | 80 | 1 | 6 |
| 11297212 | 11297293 | C | A | 6 | 0 | 0 |
| 11297218 | 11297299 | A | T | 1 | 0 | 0 |
| 11297219 | 11297300 | A | G | 28 | 0 | 0 |
| 11297247 | 11297328 | C | A | 41 | 0 | 0 |
| 11297288 | 11297369 | A | T | 8 | 0 | 0 |
| 11297296 | 11297377 | A | G | 67 | 4 | 31 |
| - | 11297444 | - | G | 49 | 0 | 0 |
| 11297411 | 11297493 | A | - | 1 | 0 | 0 |
| 11297412 | 11297494 | G | T | 32 | 0 | 0 |
| 11297444 | 11297526 | A | G | 10 | 0 | 0 |
| 11297454 | 11297536 | T | C | 7 | 0 | 0 |
| 11297461 | 11297543 | T | G | 31 | 0 | 0 |
| 11297492 | 11297574 | A | G | 56 | 0 | 0 |
| 11297548 | 11297630 | G | A | - | 0 | 0 |
