# Supplementary material for: Interhomolog polymorphism shapes meiotic crossover within the Arabidopsis RAC1 and RPP13 disease resistance genes
Source: PLoS Genet. 2018 Dec 13;14(12):e1007843. doi: 10.1371/journal.pgen.1007843 (PMC6307820; doi:10.1371/journal.pgen.1007843)
Supplement: S11 Table — The window size used was 500 bp, within which crossovers (CO) were counted, and the number of polymorphisms (Polys.), where SNPs were counted as one and indels by their length in bp. Analysis was performed against the pancoordinates. The row highlighted in grey is the final window which has a different size in each cross due to variable panmolecule lengths. This window was not included in the analysis plotted in Fig 4. (DOCX) [file pgen.1007843.s016.docx]

**S11 Table**. **Adjacent window analysis of polymorphisms versus crossovers within the *RAC1* pollen-typing amplicon.**

|  | Col×Ler | | Col×Wl | | Col×Mh | |
| --- | --- | --- | --- | --- | --- | --- |
| Pancoordinates | Polys. | COs | Polys. | COs | Polys. | COs |
| 11,288,165 - 11,288,665 | 36 | 0 | 37 | 2 | 14 | 0.4 |
| 11,288,665 - 11,289,165 | 18 | 10 | 19 | 2.8 | 3 | 2.6 |
| 11,289,165 - 11,289,665 | 5 | 9.7 | 9 | 4.2 | 220 | 0 |
| 11,289,665 - 11,290,165 | 9 | 12.3 | 71 | 1 | 29 | 2.5 |
| 11,290,165 - 11,290,665 | 14 | 8.8 | 22 | 4.9 | 24 | 4.5 |
| 11,290,665 - 11,291,165 | 69 | 0.2 | 42 | 2.1 | 67 | 1.3 |
| 11,291,165 - 11,291,665 | 28 | 4 | 27 | 3 | 18 | 5.5 |
| 11,291,665 - 11,292,165 | 50 | 4.3 | 9 | 14 | 11 | 11.5 |
| 11,292,165 - 11,292,665 | 5 | 23.1 | 2 | 11.2 | 7 | 16.7 |
| 11,292,665 - 11,293,165 | 9 | 51.6 | 4 | 11.2 | 7 | 18.2 |
| 11,293,165 - 11,293,665 | 3 | 62.2 | 7 | 9.6 | 0 | 23.7 |
| 11,293,665 - 11,294,165 | 13 | 21 | 11 | 8 | 17 | 8.1 |
| 11,294,165 - 11,294,665 | 58 | 4.8 | 59 | 1 | 27 | 5.7 |
| 11,294,665 - 11,295,165 | 29 | 0 | 32 | 1.2 | 4 | 1.9 |
| 11,295,165 - 11,295,665 | 35 | 1 | 36 | 0.8 | 10 | 2.4 |
| 11,295,665 - 11,296,165 | 10 | 5 | 10 | 4.7 | 11 | 6 |
| 11,296,165 - 11,296,665 | 10 | 12.1 | 9 | 6.3 | 9 | 5.2 |
| 11,296,665 - 11,297,165 | 43 | 7.9 | 47 | 4 | 44 | 2.8 |
| 11,297,165  – … | 15 | 2 | 15 | 0 | 15 | 5 |
| Total | 459 | 240 | 468 | 92 | 537 | 124 |
