# Supplementary material for: Interhomolog polymorphism shapes meiotic crossover within the Arabidopsis RAC1 and RPP13 disease resistance genes
Source: PLoS Genet. 2018 Dec 13;14(12):e1007843. doi: 10.1371/journal.pgen.1007843 (PMC6307820; doi:10.1371/journal.pgen.1007843)
Supplement: S13 Table — Recombination rate was calculated using the Col×Ler panmolecule distance between the pollen-typing inner ASOs (9,482 bp). (DOCX) [file pgen.1007843.s018.docx]

**S13 Table. Genetic distance within the *RAC1* amplicon in wild type and genetic backgrounds with altered meiotic recombination.**

|  | WT | *recq4a recq4b* | *fancm* | *recq4a recq4b fancm* |
| --- | --- | --- | --- | --- |
| Parentals/μl | 10,618.8 | 24,396.7 | 18,467.2 | 18,754.9 |
| Crossovers/μl | 10.07 | 14.47 | 15.28 | 10.22 |
| cM | 0.095 | 0.059 | 0.083 | 0.055 |
| cM S.D. | 0.013 | 0.009 | 0.009 | 0.007 |
| cM/Mb | 10.01 | 6.22 | 8.75 | 5.80 |

|  | WT | *figl* | *figl fancm* | *HEI10* |
| --- | --- | --- | --- | --- |
| Parentals/μl | 6,447.4 | 8,909.8 | 11,730.7 | 8,557.3 |
| Crossovers/μl | 5.89 | 6.49 | 7.19 | 8.30 |
| cM | 0.091 | 0.073 | 0.061 | 0.097 |
| cM S.D. | 0.011 | 0.011 | 0.006 | 0.010 |
| cM/Mb | 9.60 | 7.70 | 6.43 | 10.23 |
