# Supplementary material for: Interhomolog polymorphism shapes meiotic crossover within the Arabidopsis RAC1 and RPP13 disease resistance genes
Source: PLoS Genet. 2018 Dec 13;14(12):e1007843. doi: 10.1371/journal.pgen.1007843 (PMC6307820; doi:10.1371/journal.pgen.1007843)
Supplement: S14 Table — Crossover events were identified using Sanger sequencing of amplifications from single molecules. Interval lengths are calculated according to the Col×Ler panmolecule, and these distances are used to calculate cM/Mb. (DOCX) [file pgen.1007843.s019.docx]

**S14 Table. Crossover distributions across the *RAC1* *R* gene hotspot in Col×Ler F_1_ analysed via pollen-typing in wild type, *recq4a recq4b*, *fancm* and *recq4a recq4b fancm*.**

|  |  |  | wild type | | *recq4a recq4b* | | *fancm* | | *recq4a recq4b fancm* | |
| --- | --- | --- | --- | --- | --- | --- | --- | --- | --- | --- |
| TAIR10 coordinates | Pan coordinates | Interval length (bp) | COs | cM/Mb | COs | cM/Mb | COs | cM/Mb | COs | cM/Mb |
| 11288165 | 11288165 | 16 | 0 | 0 | 0 | 0 | 0 | 0 | 0 | 0 |
| 11288181 | 11288181 | 11 | 0 | 0 | 0 | 0 | 0 | 0 | 0 | 0 |
| 11288192 | 11288192 | 7 | 0 | 0 | 0 | 0 | 0 | 0 | 0 | 0 |
| 11288199 | 11288199 | 9 | 0 | 0 | 0 | 0 | 0 | 0 | 0 | 0 |
| - | 11288208 | 55 | 0 | 0 | 0 | 0 | 0 | 0 | 0 | 0 |
| 11288241 | 11288263 | 1 | 0 | 0 | 0 | 0 | 0 | 0 | 0 | 0 |
| 11288242 | 11288264 | 9 | 0 | 0 | 0 | 0 | 0 | 0 | 0 | 0 |
| 11288251 | 11288273 | 1 | 0 | 0 | 0 | 0 | 0 | 0 | 0 | 0 |
| 11288252 | 11288274 | 12 | 0 | 0 | 0 | 0 | 0 | 0 | 0 | 0 |
| - | 11288286 | 69 | 0 | 0 | 0 | 0 | 0 | 0 | 0 | 0 |
| 11288331 | 11288355 | 127 | 0 | 0 | 0 | 0 | 0 | 0 | 2 | 17.0 |
| 11288458 | 11288482 | 108 | 0 | 0 | 1 | 11.6 | 0 | 0 | 0 | 0 |
| 11288566 | 11288590 | 19 | 0 | 0 | 0 | 0 | 0 | 0 | 0 | 0 |
| 11288585 | 11288609 | 77 | 0 | 0 | 0 | 0 | 0 | 0 | 0 | 0 |
| 11288662 | 11288686 | 76 | 1 | 21.2 | 0 | 0 | 0 | 0 | 0 | 0 |
| 11288738 | 11288762 | 62 | 0 | 0 | 0 | 0 | 0 | 0 | 0 | 0 |
| 11288800 | 11288824 | 72 | 0 | 0 | 0 | 0 | 0 | 0 | 0 | 0 |
| 11288872 | 11288896 | 9 | 0 | 0 | 1 | 139.5 | 0 | 0 | 0 | 0 |
| 11288881 | 11288905 | 17 | 0 | 0 | 0 | 0 | 0 | 0 | 0 | 0 |
| 11288898 | 11288922 | 23 | 0 | 0 | 0 | 0 | 0 | 0 | 0 | 0 |
| 11288921 | 11288945 | 8 | 0 | 0 | 0 | 0 | 0 | 0 | 0 | 0 |
| 11288929 | 11288953 | 1 | 0 | 0 | 0 | 0 | 0 | 0 | 0 | 0 |
| 11288930 | 11288954 | 19 | 0 | 0 | 0 | 0 | 0 | 0 | 0 | 0 |
| 11288949 | 11288973 | 148 | 0 | 0 | 0 | 0 | 0 | 0 | 1 | 7.3 |
| 11289097 | 11289121 | 178 | 0 | 0 | 3 | 21.2 | 3 | 28.5 | 1 | 6.1 |
| 11289275 | 11289299 | 118 | 1 | 13.6 | 0 | 0 | 0 | 0 | 1 | 9.1 |
| 11289393 | 11289417 | 53 | 1 | 30.4 | 0 | 0 | 2 | 63.9 | 1 | 20.3 |
| 11289446 | 11289470 | 5 | 0 | 0 | 0 | 0 | 0 | 0 | 0 | 0 |
| 11289451 | 11289475 | 103 | 0 | 0 | 1 | 12.2 | 0 | 0 | 0 | 0 |
| 11289554 | 11289578 | 130 | 0 | 0 | 1 | 9.7 | 1 | 13.0 | 0 | 0 |
| 11289684 | 11289708 | 99 | 0 | 0 | 2 | 25.4 | 2 | 34.2 | 0 | 0 |
| 11289783 | 11289807 | 78 | 0 | 0 | 1 | 16.1 | 0 | 0 | 3 | 41.5 |
| 11289861 | 11289885 | 80 | 1 | 20.1 | 2 | 31.4 | 1 | 21.2 | 1 | 13.5 |
| 11289941 | 11289965 | 75 | 0 | 0 | 0 | 0 | 0 | 0 | 1 | 14.4 |
| 11290016 | 11290040 | 52 | 1 | 31.0 | 0 | 0 | 0 | 0 | 0 | 0 |
| 11290068 | 11290092 | 69 | 0 | 0 | 0 | 0 | 0 | 0 | 0 | 0 |
| 11290137 | 11290161 | 22 | 0 | 0 | 0 | 0 | 0 | 0 | 0 | 0 |
| 11290159 | 11290183 | 28 | 1 | 57.5 | 0 | 0 | 0 | 0 | 0 | 0 |
| 11290187 | 11290211 | 10 | 0 | 0 | 0 | 0 | 0 | 0 | 0 | 0 |
| 11290197 | 11290221 | 10 | 0 | 0 | 0 | 0 | 0 | 0 | 0 | 0 |
| 11290207 | 11290231 | 186 | 3 | 26.0 | 3 | 20.2 | 2 | 18.2 | 5 | 29.0 |
| 11290393 | 11290417 | 2 | 0 | 0 | 0 | 0 | 0 | 0 | 0 | 0 |
| 11290395 | 11290419 | 1 | 0 | 0 | 0 | 0 | 0 | 0 | 0 | 0 |
| 11290396 | 11290420 | 2 | 0 | 0 | 0 | 0 | 0 | 0 | 0 | 0 |
| 11290398 | 11290422 | 1 | 0 | 0 | 0 | 0 | 0 | 0 | 0 | 0 |
| 11290399 | 11290423 | 5 | 0 | 0 | 0 | 0 | 0 | 0 | 0 | 0 |
| 11290404 | 11290428 | 6 | 0 | 0 | 0 | 0 | 0 | 0 | 0 | 0 |
| 11290410 | 11290434 | 33 | 0 | 0 | 0 | 0 | 0 | 0 | 0 | 0 |
| 11290443 | 11290467 | 21 | 0 | 0 | 0 | 0 | 0 | 0 | 0 | 0 |
| 11290464 | 11290488 | 121 | 1 | 13.3 | 1 | 10.4 | 3 | 42.0 | 0 | 0 |
| 11290585 | 11290609 | 67 | 0 | 0 | 0 | 0 | 0 | 0 | 0 | 0 |
| 11290652 | 11290676 | 2 | 0 | 0 | 0 | 0 | 0 | 0 | 0 | 0 |
| 11290654 | 11290678 | 8 | 0 | 0 | 0 | 0 | 0 | 0 | 0 | 0 |
| 11290662 | 11290686 | 2 | 0 | 0 | 0 | 0 | 0 | 0 | 0 | 0 |
| - | 11290688 | 55 | 0 | 0 | 0 | 0 | 0 | 0 | 0 | 0 |
| 11290687 | 11290743 | 26 | 0 | 0 | 0 | 0 | 0 | 0 | 0 | 0 |
| 11290713 | 11290769 | 27 | 0 | 0 | 0 | 0 | 0 | 0 | 0 | 0 |
| 11290740 | 11290796 | 13 | 0 | 0 | 0 | 0 | 0 | 0 | 0 | 0 |
| 11290753 | 11290809 | 29 | 0 | 0 | 0 | 0 | 0 | 0 | 0 | 0 |
| 11290782 | 11290838 | 95 | 0 | 0 | 0 | 0 | 1 | 17.8 | 0 | 0 |
| 11290877 | 11290933 | 6 | 0 | 0 | 0 | 0 | 0 | 0 | 0 | 0 |
| 11290883 | 11290939 | 6 | 0 | 0 | 0 | 0 | 0 | 0 | 0 | 0 |
| 11290889 | 11290945 | 10 | 0 | 0 | 0 | 0 | 0 | 0 | 0 | 0 |
| 11290899 | 11290955 | 67 | 0 | 0 | 0 | 0 | 0 | 0 | 0 | 0 |
| 11290966 | 11291022 | 13 | 0 | 0 | 0 | 0 | 0 | 0 | 0 | 0 |
| 11290979 | 11291035 | 31 | 0 | 0 | 0 | 0 | 0 | 0 | 0 | 0 |
| 11291010 | 11291066 | 1 | 0 | 0 | 0 | 0 | 0 | 0 | 0 | 0 |
| - | 11291067 | 38 | 0 | 0 | 0 | 0 | 0 | 0 | 0 | 0 |
| 11291032 | 11291105 | 4 | 0 | 0 | 0 | 0 | 0 | 0 | 0 | 0 |
| 11291036 | 11291109 | 20 | 0 | 0 | 0 | 0 | 0 | 0 | 0 | 0 |
| 11291056 | 11291129 | 105 | 0 | 0 | 0 | 0 | 3 | 48.4 | 0 | 0 |
| 11291161 | 11291234 | 19 | 0 | 0 | 0 | 0 | 0 | 0 | 0 | 0 |
| 11291180 | 11291253 | 83 | 0 | 0 | 0 | 0 | 0 | 0 | 0 | 0 |
| 11291263 | 11291336 | 9 | 0 | 0 | 0 | 0 | 0 | 0 | 0 | 0 |
| 11291272 | 11291345 | 62 | 0 | 0 | 0 | 0 | 0 | 0 | 0 | 0 |
| 11291334 | 11291407 | 7 | 0 | 0 | 0 | 0 | 0 | 0 | 0 | 0 |
| 11291341 | 11291414 | 13 | 0 | 0 | 0 | 0 | 0 | 0 | 0 | 0 |
| 11291354 | 11291427 | 36 | 0 | 0 | 0 | 0 | 0 | 0 | 1 | 30.0 |
| 11291390 | 11291463 | 6 | 0 | 0 | 0 | 0 | 0 | 0 | 0 | 0 |
| 11291396 | 11291469 | 2 | 0 | 0 | 0 | 0 | 0 | 0 | 0 | 0 |
| 11291398 | 11291471 | 5 | 1 | 322.0 | 0 | 0 | 0 | 0 | 0 | 0 |
| 11291403 | 11291476 | 4 | 0 | 0 | 0 | 0 | 0 | 0 | 0 | 0 |
| 11291407 | 11291480 | 13 | 0 | 0 | 0 | 0 | 0 | 0 | 0 | 0 |
| 11291420 | 11291493 | 15 | 0 | 0 | 0 | 0 | 0 | 0 | 0 | 0 |
| 11291435 | 11291508 | 2 | 0 | 0 | 0 | 0 | 0 | 0 | 0 | 0 |
| 11291437 | 11291510 | 1 | 0 | 0 | 0 | 0 | 0 | 0 | 0 | 0 |
| 11291438 | 11291511 | 28 | 1 | 57.5 | 0 | 0 | 0 | 0 | 0 | 0 |
| 11291466 | 11291539 | 9 | 0 | 0 | 0 | 0 | 0 | 0 | 0 | 0 |
| 11291475 | 11291548 | 1 | 0 | 0 | 0 | 0 | 0 | 0 | 0 | 0 |
| 11291476 | 11291549 | 54 | 0 | 0 | 0 | 0 | 0 | 0 | 0 | 0 |
| 11291530 | 11291603 | 2 | 0 | 0 | 0 | 0 | 0 | 0 | 0 | 0 |
| 11291532 | 11291605 | 8 | 0 | 0 | 0 | 0 | 0 | 0 | 0 | 0 |
| 11291540 | 11291613 | 17 | 0 | 0 | 0 | 0 | 0 | 0 | 0 | 0 |
| 11291557 | 11291630 | 23 | 0 | 0 | 0 | 0 | 0 | 0 | 0 | 0 |
| 11291580 | 11291653 | 8 | 0 | 0 | 0 | 0 | 0 | 0 | 0 | 0 |
| 11291588 | 11291661 | 2 | 0 | 0 | 0 | 0 | 0 | 0 | 0 | 0 |
| 11291590 | 11291663 | 28 | 0 | 0 | 0 | 0 | 0 | 0 | 0 | 0 |
| 11291618 | 11291691 | 3 | 0 | 0 | 0 | 0 | 0 | 0 | 0 | 0 |
| 11291621 | 11291694 | 29 | 0 | 0 | 0 | 0 | 0 | 0 | 0 | 0 |
| 11291650 | 11291723 | 4 | 0 | 0 | 0 | 0 | 0 | 0 | 0 | 0 |
| 11291654 | 11291727 | 4 | 0 | 0 | 0 | 0 | 0 | 0 | 0 | 0 |
| 11291658 | 11291731 | 32 | 0 | 0 | 0 | 0 | 0 | 0 | 1 | 33.7 |
| 11291690 | 11291763 | 72 | 1 | 22.4 | 0 | 0 | 0 | 0 | 0 | 0 |
| 11291762 | 11291835 | 17 | 0 | 0 | 0 | 0 | 0 | 0 | 0 | 0 |
| 11291779 | 11291852 | 9 | 0 | 0 | 0 | 0 | 0 | 0 | 0 | 0 |
| 11291788 | 11291861 | 4 | 0 | 0 | 0 | 0 | 0 | 0 | 0 | 0 |
| 11291792 | 11291865 | 4 | 0 | 0 | 0 | 0 | 0 | 0 | 0 | 0 |
| 11291796 | 11291869 | 22 | 0 | 0 | 0 | 0 | 0 | 0 | 0 | 0 |
| 11291818 | 11291891 | 50 | 0 | 0 | 0 | 0 | 0 | 0 | 0 | 0 |
| 11291868 | 11291941 | 11 | 0 | 0 | 0 | 0 | 0 | 0 | 0 | 0 |
| 11291879 | 11291952 | 38 | 0 | 0 | 0 | 0 | 0 | 0 | 0 | 0 |
| 11291917 | 11291990 | 13 | 0 | 0 | 0 | 0 | 0 | 0 | 0 | 0 |
| - | 11292003 | 4 | 0 | 0 | 0 | 0 | 0 | 0 | 0 | 0 |
| - | 11292007 | 10 | 0 | 0 | 0 | 0 | 0 | 0 | 0 | 0 |
| 11291940 | 11292017 | 10 | 0 | 0 | 0 | 0 | 0 | 0 | 0 | 0 |
| 11291950 | 11292027 | 12 | 1 | 134.2 | 0 | 0 | 0 | 0 | 0 | 0 |
| 11291962 | 11292039 | 7 | 0 | 0 | 0 | 0 | 0 | 0 | 0 | 0 |
| 11291969 | 11292046 | 2 | 0 | 0 | 0 | 0 | 0 | 0 | 0 | 0 |
| 11291971 | 11292048 | 1 | 0 | 0 | 0 | 0 | 0 | 0 | 0 | 0 |
| 11291972 | 11292049 | 3 | 0 | 0 | 0 | 0 | 0 | 0 | 0 | 0 |
| 11291975 | 11292052 | 15 | 0 | 0 | 0 | 0 | 0 | 0 | 0 | 0 |
| 11291990 | 11292067 | 5 | 0 | 0 | 0 | 0 | 0 | 0 | 0 | 0 |
| 11291995 | 11292072 | 18 | 0 | 0 | 0 | 0 | 0 | 0 | 0 | 0 |
| 11292013 | 11292090 | 17 | 0 | 0 | 0 | 0 | 0 | 0 | 0 | 0 |
| 11292030 | 11292107 | 1 | 0 | 0 | 0 | 0 | 0 | 0 | 0 | 0 |
| 11292031 | 11292108 | 1 | 0 | 0 | 0 | 0 | 0 | 0 | 0 | 0 |
| 11292032 | 11292109 | 26 | 0 | 0 | 1 | 48.3 | 0 | 0 | 0 | 0 |
| 11292058 | 11292135 | 27 | 1 | 59.6 | 0 | 0 | 0 | 0 | 0 | 0 |
| 11292085 | 11292162 | 130 | 2 | 24.8 | 1 | 9.7 | 4 | 52.1 | 1 | 8.3 |
| 11292215 | 11292292 | 53 | 1 | 30.4 | 0 | 0 | 0 | 0 | 0 | 0 |
| 11292268 | 11292345 | 111 | 1 | 14.5 | 1 | 11.3 | 0 | 0 | 0 | 0 |
| 11292379 | 11292456 | 89 | 3 | 54.3 | 0 | 0 | 0 | 0 | 1 | 12.1 |
| 11292468 | 11292545 | 116 | 0 | 0 | 3 | 32.5 | 0 | 0 | 1 | 9.3 |
| 11292584 | 11292661 | 21 | 0 | 0 | 0 | 0 | 0 | 0 | 0 | 0 |
| 11292605 | 11292682 | 10 | 0 | 0 | 0 | 0 | 0 | 0 | 0 | 0 |
| 11292615 | 11292692 | 148 | 2 | 21.8 | 4 | 33.9 | 0 | 0 | 4 | 29.1 |
| 11292763 | 11292840 | 68 | 3 | 71.0 | 0 | 0 | 1 | 24.9 | 0 | 0 |
| 11292831 | 11292908 | 55 | 3 | 87.8 | 0 | 0 | 1 | 30.8 | 0 | 0 |
| 11292886 | 11292963 | 28 | 0 | 0 | 1 | 44.8 | 1 | 60.5 | 0 | 0 |
| 11292914 | 11292991 | 69 | 2 | 46.7 | 0 | 0 | 2 | 49.1 | 0 | 0 |
| 11292983 | 11293060 | 32 | 0 | 0 | 0 | 0 | 0 | 0 | 0 | 0 |
| 11293015 | 11293092 | 73 | 1 | 22.1 | 1 | 17.2 | 0 | 0 | 0 | 0 |
| 11293088 | 11293165 | 136 | 6 | 71.0 | 2 | 18.5 | 2 | 24.9 | 3 | 23.8 |
| - | 11293301 | 257 | 8 | 50.1 | 8 | 39.1 | 7 | 46.1 | 8 | 33.6 |
| 11293479 | 11293558 | 210 | 3 | 23.0 | 2 | 12.0 | 4 | 32.3 | 7 | 35.9 |
| 11293689 | 11293768 | 9 | 0 | 0 | 0 | 0 | 1 | 188.2 | 0 | 0 |
| - | 11293777 | 17 | 1 | 94.7 | 0 | 0 | 0 | 0 | 0 | 0 |
| 11293713 | 11293794 | 29 | 0 | 0 | 0 | 0 | 0 | 0 | 0 | 0 |
| 11293742 | 11293823 | 56 | 3 | 86.3 | 0 | 0 | 1 | 30.2 | 0 | 0 |
| 11293798 | 11293879 | 133 | 1 | 12.1 | 2 | 18.9 | 0 | 0,0 | 1 | 8.1 |
| 11293931 | 11294012 | 49 | 0 | 0 | 0 | 0 | 1 | 34.6 | 1 | 22.0 |
| 11293980 | 11294061 | 9 | 0 | 0 | 0 | 0 | 0 | 0 | 0 | 0 |
| 11293989 | 11294070 | 1 | 0 | 0 | 0 | 0 | 0 | 0 | 0 | 0 |
| 11293990 | 11294071 | 17 | 0 | 0 | 0 | 0 | 0 | 0 | 0 | 0 |
| 11294007 | 11294088 | 36 | 0 | 0 | 0 | 0 | 0 | 0 | 0 | 0 |
| 11294043 | 11294124 | 15 | 0 | 0 | 0 | 0 | 0 | 0 | 0 | 0 |
| 11294058 | 11294139 | 36 | 0 | 0 | 0 | 0 | 0 | 0 | 0 | 0 |
| 11294094 | 11294175 | 14 | 0 | 0 | 0 | 0 | 0 | 0 | 0 | 0 |
| 11294108 | 11294189 | 20 | 0 | 0 | 0 | 0 | 0 | 0 | 0 | 0 |
| - | 11294209 | 13 | 0 | 0 | 0 | 0 | 0 | 0 | 0 | 0 |
| 11294139 | 11294222 | 2 | 0 | 0 | 0 | 0 | 0 | 0 | 0 | 0 |
| 11294141 | 11294224 | 37 | 0 | 0 | 0 | 0 | 0 | 0 | 0 | 0 |
| 11294178 | 11294261 | 6 | 0 | 0 | 0 | 0 | 0 | 0 | 0 | 0 |
| 11294184 | 11294267 | 26 | 0 | 0 | 0 | 0 | 0 | 0 | 0 | 0 |
| 11294210 | 11294293 | 7 | 0 | 0 | 0 | 0 | 0 | 0 | 0 | 0 |
| 11294217 | 11294300 | 119 | 1 | 13.5 | 0 | 0 | 0 | 0 | 0 | 0 |
| 11294336 | 11294419 | 13 | 0 | 0 | 0 | 0 | 0 | 0 | 0 | 0 |
| 11294349 | 11294432 | 2 | 0 | 0 | 0 | 0 | 0 | 0 | 0 | 0 |
| 11294351 | 11294434 | 8 | 0 | 0 | 0 | 0 | 0 | 0 | 0 | 0 |
| 11294359 | 11294442 | 8 | 0 | 0 | 0 | 0 | 0 | 0 | 0 | 0 |
| 11294367 | 11294450 | 1 | 0 | 0 | 0 | 0 | 0 | 0 | 0 | 0 |
| 11294368 | 11294451 | 16 | 0 | 0 | 0 | 0 | 0 | 0 | 0 | 0 |
| 11294384 | 11294467 | 3 | 0 | 0 | 0 | 0 | 0 | 0 | 0 | 0 |
| 11294387 | 11294470 | 5 | 0 | 0 | 0 | 0 | 0 | 0 | 0 | 0 |
| 11294392 | 11294475 | 4 | 0 | 0 | 0 | 0 | 0 | 0 | 0 | 0 |
| 11294396 | 11294479 | 48 | 0 | 0 | 0 | 0 | 0 | 0 | 0 | 0 |
| 11294444 | 11294527 | 25 | 0 | 0 | 0 | 0 | 0 | 0 | 0 | 0 |
| 11294469 | 11294552 | 21 | 0 | 0 | 0 | 0 | 0 | 0 | 0 | 0 |
| 11294490 | 11294573 | 9 | 0 | 0 | 0 | 0 | 0 | 0 | 0 | 0 |
| 11294499 | 11294582 | 1 | 0 | 0 | 0 | 0 | 0 | 0 | 0 | 0 |
| 11294500 | 11294583 | 6 | 0 | 0 | 0 | 0 | 0 | 0 | 0 | 0 |
| - | 11294589 | 20 | 0 | 0 | 0 | 0 | 0 | 0 | 0 | 0 |
| 11294525 | 11294609 | 4 | 0 | 0 | 0 | 0 | 0 | 0 | 0 | 0 |
| 11294529 | 11294613 | 9 | 0 | 0 | 0 | 0 | 0 | 0 | 0 | 0 |
| - | 11294622 | 2 | 0 | 0 | 0 | 0 | 0 | 0 | 0 | 0 |
| 11294539 | 11294624 | 11 | 0 | 0 | 0 | 0 | 0 | 0 | 0 | 0 |
| 11294550 | 11294635 | 21 | 0 | 0 | 0 | 0 | 0 | 0 | 0 | 0 |
| 11294571 | 11294656 | 2 | 0 | 0 | 0 | 0 | 0 | 0 | 0 | 0 |
| 11294573 | 11294658 | 52 | 0 | 0 | 0 | 0 | 0 | 0 | 0 | 0 |
| 11294625 | 11294710 | 28 | 0 | 0 | 0 | 0 | 0 | 0 | 0 | 0 |
| 11294653 | 11294738 | 7 | 0 | 0 | 0 | 0 | 0 | 0 | 0 | 0 |
| 11294660 | 11294745 | 9 | 0 | 0 | 0 | 0 | 0 | 0 | 0 | 0 |
| 11294669 | 11294754 | 2 | 0 | 0 | 0 | 0 | 0 | 0 | 0 | 0 |
| 11294671 | 11294756 | 11 | 0 | 0 | 0 | 0 | 0 | 0 | 0 | 0 |
| 11294682 | 11294767 | 6 | 0 | 0 | 0 | 0 | 0 | 0 | 0 | 0 |
| 11294688 | 11294773 | 6 | 0 | 0 | 0 | 0 | 0 | 0 | 0 | 0 |
| 11294694 | 11294779 | 21 | 0 | 0 | 0 | 0 | 0 | 0 | 0 | 0 |
| 11294715 | 11294800 | 27 | 0 | 0 | 0 | 0 | 0 | 0 | 0 | 0 |
| 11294742 | 11294827 | 17 | 0 | 0 | 0 | 0 | 0 | 0 | 0 | 0 |
| 11294759 | 11294844 | 15 | 0 | 0 | 0 | 0 | 0 | 0 | 0 | 0 |
| - | 11294859 | 5 | 0 | 0 | 0 | 0 | 0 | 0 | 0 | 0 |
| 11294777 | 11294864 | 26 | 0 | 0 | 0 | 0 | 0 | 0 | 0 | 0 |
| - | 11294890 | 11 | 0 | 0 | 0 | 0 | 0 | 0 | 0 | 0 |
| 11294813 | 11294901 | 23 | 0 | 0 | 0 | 0 | 0 | 0 | 0 | 0 |
| - | 11294924 | 41 | 0 | 0 | 0 | 0 | 0 | 0 | 0 | 0 |
| 11294874 | 11294965 | 31 | 0 | 0 | 0 | 0 | 0 | 0 | 0 | 0 |
| 11294905 | 11294996 | 11 | 0 | 0 | 0 | 0 | 0 | 0 | 0 | 0 |
| 11294916 | 11295007 | 19 | 0 | 0 | 0 | 0 | 0 | 0 | 0 | 0 |
| 11294935 | 11295026 | 2 | 0 | 0 | 0 | 0 | 0 | 0 | 0 | 0 |
| 11294937 | 11295028 | 23 | 0 | 0 | 0 | 0 | 0 | 0 | 0 | 0 |
| 11294960 | 11295051 | 20 | 0 | 0 | 0 | 0 | 0 | 0 | 0 | 0 |
| 11294980 | 11295071 | 11 | 0 | 0 | 0 | 0 | 0 | 0 | 0 | 0 |
| 11294991 | 11295082 | 47 | 0 | 0 | 0 | 0 | 0 | 0 | 0 | 0 |
| 11295038 | 11295129 | 53 | 0 | 0 | 0 | 0 | 0 | 0 | 0 | 0 |
| 11295091 | 11295182 | 35 | 0 | 0 | 0 | 0 | 0 | 0 | 0 | 0 |
| 11295126 | 11295217 | 25 | 0 | 0 | 0 | 0 | 0 | 0 | 0 | 0 |
| 11295151 | 11295242 | 17 | 0 | 0 | 0 | 0 | 0 | 0 | 0 | 0 |
| 11295168 | 11295259 | 4 | 0 | 0 | 0 | 0 | 0 | 0 | 0 | 0 |
| 11295172 | 11295263 | 1 | 0 | 0 | 0 | 0 | 0 | 0 | 0 | 0 |
| 11295173 | 11295264 | 9 | 0 | 0 | 0 | 0 | 0 | 0 | 0 | 0 |
| 11295182 | 11295273 | 5 | 0 | 0 | 0 | 0 | 0 | 0 | 0 | 0 |
| 11295187 | 11295278 | 6 | 0 | 0 | 0 | 0 | 0 | 0 | 0 | 0 |
| 11295193 | 11295284 | 2 | 0 | 0 | 0 | 0 | 0 | 0 | 0 | 0 |
| 11295195 | 11295286 | 22 | 0 | 0 | 0 | 0 | 1 | 77.0 | 0 | 0 |
| 11295217 | 11295308 | 17 | 0 | 0 | 0 | 0 | 0 | 0 | 0 | 0 |
| 11295234 | 11295325 | 28 | 0 | 0 | 0 | 0 | 0 | 0 | 0 | 0 |
| 11295262 | 11295353 | 11 | 0 | 0 | 0 | 0 | 0 | 0 | 0 | 0 |
| 11295273 | 11295364 | 16 | 0 | 0 | 0 | 0 | 0 | 0 | 0 | 0 |
| 11295289 | 11295380 | 1 | 0 | 0 | 0 | 0 | 0 | 0 | 0 | 0 |
| 11295290 | 11295381 | 9 | 0 | 0 | 0 | 0 | 0 | 0 | 0 | 0 |
| 11295299 | 11295390 | 52 | 0 | 0 | 0 | 0 | 0 | 0 | 0 | 0 |
| 11295351 | 11295442 | 7 | 0 | 0 | 0 | 0 | 0 | 0 | 0 | 0 |
| - | 11295449 | 7 | 0 | 0 | 0 | 0 | 0 | 0 | 0 | 0 |
| 11295364 | 11295456 | 11 | 0 | 0 | 0 | 0 | 0 | 0 | 0 | 0 |
| 11295375 | 11295467 | 3 | 0 | 0 | 0 | 0 | 0 | 0 | 0 | 0 |
| 11295378 | 11295470 | 1 | 0 | 0 | 0 | 0 | 0 | 0 | 0 | 0 |
| 11295379 | 11295471 | 9 | 0 | 0 | 0 | 0 | 0 | 0 | 0 | 0 |
| 11295388 | 11295480 | 18 | 0 | 0 | 0 | 0 | 0 | 0 | 0 | 0 |
| 11295406 | 11295498 | 37 | 0 | 0 | 0 | 0 | 0 | 0 | 0 | 0 |
| 11295443 | 11295535 | 36 | 0 | 0 | 0 | 0 | 1 | 47.1 | 0 | 0 |
| 11295479 | 11295571 | 17 | 0 | 0 | 0 | 0 | 0 | 0 | 0 | 0 |
| 11295496 | 11295588 | 28 | 0 | 0 | 0 | 0 | 0 | 0 | 0 | 0 |
| 11295524 | 11295616 | 17 | 0 | 0 | 0 | 0 | 0 | 0 | 0 | 0 |
| 11295541 | 11295633 | 3 | 0 | 0 | 0 | 0 | 0 | 0 | 0 | 0 |
| 11295544 | 11295636 | 16 | 0 | 0 | 0 | 0 | 0 | 0 | 0 | 0 |
| 11295560 | 11295652 | 5 | 0 | 0 | 0 | 0 | 0 | 0 | 0 | 0 |
| 11295565 | 11295657 | 3 | 0 | 0 | 0 | 0 | 0 | 0 | 0 | 0 |
| 11295568 | 11295660 | 33 | 0 | 0 | 0 | 0 | 0 | 0 | 0 | 0 |
| 11295601 | 11295693 | 8 | 0 | 0 | 0 | 0 | 0 | 0 | 0 | 0 |
| 11295609 | 11295701 | 127 | 0 | 0 | 1 | 9.9 | 0 | 0 | 0 | 0 |
| 11295736 | 11295828 | 154 | 1 | 10.5 | 1 | 8.2 | 0 | 0 | 0 | 0 |
| 11295890 | 11295982 | 31 | 0 | 0 | 0 | 0 | 0 | 0 | 0 | 0 |
| 11295921 | 11296013 | 5 | 0 | 0 | 0 | 0 | 0 | 0 | 0 | 0 |
| - | 11296018 | 30 | 0 | 0 | 0 | 0 | 0 | 0 | 0 | 0 |
| 11295953 | 11296048 | 41 | 0 | 0 | 0 | 0 | 0 | 0 | 1 | 26.3 |
| 11295994 | 11296089 | 79 | 0 | 0 | 0 | 0 | 0 | 0 | 0 | 0 |
| 11296073 | 11296168 | 57 | 0 | 0 | 0 | 0 | 0 | 0 | 1 | 18.9 |
| 11296130 | 11296225 | 96 | 0 | 0 | 0 | 0 | 1 | 17.6 | 0 | 0 |
| 11296226 | 11296321 | 24 | 1 | 67.1 | 0 | 0 | 0 | 0 | 0 | 0 |
| 11296250 | 11296345 | 19 | 0 | 0 | 0 | 0 | 0 | 0 | 0 | 0 |
| 11296269 | 11296364 | 22 | 0 | 0 | 0 | 0 | 0 | 0 | 0 | 0 |
| 11296291 | 11296386 | 178 | 1 | 9.0 | 0 | 0 | 0 | 0 | 0 | 0 |
| 11296469 | 11296564 | 193 | 0 | 0 | 1 | 6.5 | 0 | 0 | 1 | 5.6 |
| 11296662 | 11296757 | 23 | 0 | 0 | 0 | 0 | 0 | 0 | 0 | 0 |
| 11296685 | 11296780 | 22 | 0 | 0 | 0 | 0 | 0 | 0 | 0 | 0 |
| 11296707 | 11296802 | 7 | 0 | 0 | 0 | 0 | 0 | 0 | 0 | 0 |
| 11296714 | 11296809 | 27 | 0 | 0 | 0 | 0 | 0 | 0 | 1 | 39.9 |
| 11296741 | 11296836 | 81 | 0 | 0 | 0 | 0 | 0 | 0 | 0 | 0 |
| 11296822 | 11296917 | 42 | 0 | 0 | 0 | 0 | 0 | 0 | 0 | 0 |
| 11296864 | 11296959 | 12 | 0 | 0 | 0 | 0 | 0 | 0 | 0 | 0 |
| 11296876 | 11296971 | 19 | 0 | 0 | 0 | 0 | 0 | 0 | 0 | 0 |
| 11296895 | 11296990 | 40 | 0 | 0 | 0 | 0 | 0 | 0 | 0 | 0 |
| - | 11297030 | 15 | 0 | 0 | 0 | 0 | 0 | 0 | 0 | 0 |
| 11296947 | 11297045 | 2 | 0 | 0 | 0 | 0 | 0 | 0 | 0 | 0 |
| 11296949 | 11297047 | 5 | 0 | 0 | 0 | 0 | 0 | 0 | 0 | 0 |
| 11296954 | 11297052 | 8 | 0 | 0 | 0 | 0 | 0 | 0 | 0 | 0 |
| 11296962 | 11297060 | 2 | 0 | 0 | 0 | 0 | 0 | 0 | 0 | 0 |
| 11296964 | 11297062 | 2 | 0 | 0 | 0 | 0 | 0 | 0 | 0 | 0 |
| 11296966 | 11297064 | 3 | 0 | 0 | 0 | 0 | 0 | 0 | 0 | 0 |
| 11296969 | 11297067 | 4 | 0 | 0 | 0 | 0 | 0 | 0 | 0 | 0 |
| 11296973 | 11297071 | 6 | 0 | 0 | 0 | 0 | 0 | 0 | 0 | 0 |
| 11296979 | 11297077 | 6 | 0 | 0 | 0 | 0 | 0 | 0 | 0 | 0 |
| 11296985 | 11297083 | 13 | 0 | 0 | 0 | 0 | 0 | 0 | 0 | 0 |
| 11296998 | 11297096 | 134 | 0 | 0 | 0 | 0 | 0 | 0 | 0 | 0 |
| 11297132 | 11297230 | 80 | 0 | 0 | 1 | 15.7 | 0 | 0 | 0 | 0 |
| 11297212 | 11297310 | 6 | 0 | 0 | 0 | 0 | 0 | 0 | 0 | 0 |
| 11297218 | 11297316 | 1 | 0 | 0 | 0 | 0 | 0 | 0 | 0 | 0 |
| 11297219 | 11297317 | 28 | 0 | 0 | 0 | 0 | 0 | 0 | 0 | 0 |
| 11297247 | 11297345 | 41 | 0 | 0 | 0 | 0 | 1 | 41.3 | 0 | 0 |
| 11297288 | 11297386 | 8 | 0 | 0 | 0 | 0 | 0 | 0 | 0 | 0 |
| 11297296 | 11297394 | 68 | 0 | 0 | 1 | 18.5 | 1 | 24.9 | 0 | 0 |
| - | 11297462 | 46 | 0 | 0 | 0 | 0 | 0 | 0 | 1 | 23.4 |
| 11297409 | 11297508 | 3 | 0 | 0 | 0 | 0 | 0 | 0 | 0 | 0 |
| 11297412 | 11297511 | 32 | 0 | 0 | 0 | 0 | 0 | 0 | 0 | 0 |
| 11297444 | 11297543 | 10 | 0 | 0 | 0 | 0 | 0 | 0 | 0 | 0 |
| 11297454 | 11297553 | 7 | 0 | 0 | 0 | 0 | 0 | 0 | 0 | 0 |
| 11297461 | 11297560 | 31 | 0 | 0 | 0 | 0 | 0 | 0 | 0 | 0 |
| 11297492 | 11297591 | 56 | 0 | 0 | 0 | 0 | 1 | 30.2 | 1 | 19.3 |
| 11297548 | 11297647 | 16 | 0 | 0 | 0 | 0 | 0 | 0 | 0 | 0 |
|  |  | 9383 | 59 |  | 47 |  | 49 |  | 51 |  |
