# Supplementary material for: Interhomolog polymorphism shapes meiotic crossover within the Arabidopsis RAC1 and RPP13 disease resistance genes
Source: PLoS Genet. 2018 Dec 13;14(12):e1007843. doi: 10.1371/journal.pgen.1007843 (PMC6307820; doi:10.1371/journal.pgen.1007843)
Supplement: S15 Table — Recombination rate was calculated using the Col×Ler panmolecule distance between the pollen-typing inner ASOs (9,482 bp). (DOCX) [file pgen.1007843.s020.docx]

**S15 Table. Genetic distance of the *RAC1* amplicon in wild type and *msh2* mutant in Col×Ler.**

|  | WT | *msh2* |
| --- | --- | --- |
| Parentals/μl | 3,575.7 | 2,812.9 |
| Crossovers/μl | 3.33 | 1.85 |
| cM | 0.093 | 0.066 |
| cM S.D. | 0.012 | 0.009 |
| cM/Mb | 9.81 | 6.96 |
