# Supplementary material for: Interhomolog polymorphism shapes meiotic crossover within the Arabidopsis RAC1 and RPP13 disease resistance genes
Source: PLoS Genet. 2018 Dec 13;14(12):e1007843. doi: 10.1371/journal.pgen.1007843 (PMC6307820; doi:10.1371/journal.pgen.1007843)
Supplement: S16 Table — We used the mean crossovers and parentals from wild type and mutants to construct 2×2 contingeny tables and perform Chi-square tests. (DOCX) [file pgen.1007843.s021.docx]

**S16 Table. Significance testing of genetic distance of the *RAC1* amplicon in wild type and mutant backgrounds.**

| **Genotype** | **Crossovers/μl** | **Parentals/μl** | **cM** | **St.Dev** | ***P* value** |
| --- | --- | --- | --- | --- | --- |
| Wild type1 | 10.07 | 10,618.8 | 0.095 | 0.013 | - |
| *recq4a recq4b* | 14.47 | 24,396.7 | 0.059 | 0.009 | 6.59×10^-32^ |
| *fancm* | 15.28 | 18,467.2 | 0.083 | 0.009 | 4.67×10^-7^ |
| *recq4a recq4b fancm* | 10.22 | 18,754.9 | 0.055 | 0.007 | 6.60×10^-42^ |
|  |  |  |  |  |  |
| **Genotype** | **Crossovers/μl** | **Parentals/μl** | **cM** | **St.Dev** | ***P* value** |
| Wild type 2 | 5.89 | 6,447.4 | 0.091 | 0.011 | - |
| *figl1* | 6.49 | 8,909.8 | 0.073 | 0.011 | 2.05×10^-3^ |
| *figl1 fancm* | 7.19 | 11,730.7 | 0.061 | 0.006 | 1.61×10^-14^ |
| *HEI10* | 8.3 | 8,557.3 | 0.097 | 0.01 | 0.98 |
|  |  |  |  |  |  |
| **Genotype** | **Crossovers/μl** | **Parentals/μl** | **cM** | **St.Dev** | ***P* value** |
| Wild type 3 | 3.33 | 3,575.7 | 0.093 | 0.012 | - |
| *msh2* | 1.85 | 2,812.9 | 0.066 | 0.009 | 2.94×10^-4^ |
|  |  |  |  |  |  |
| **Genotype** | **Crossovers/μl** | **Parentals/μl** | **cM** | **St.Dev** | ***P* value** |
| Wild type 1 | 10.07 | 10,618.8 | 0.095 | 0.013 | - |
| Wild type 2 | 5.89 | 6,447.4 | 0.091 | 0.011 | 0.23 |
| Wild type 3 | 3.33 | 3,575.7 | 0.093 | 0.012 | 0.42 |
| Wild type 2 | 5.89 | 6,447.4 | 0.091 | 0.011 | - |
| Wild type 3 | 3.33 | 3,575.7 | 0.093 | 0.012 | 0.92 |
