# Supplementary material for: Interhomolog polymorphism shapes meiotic crossover within the Arabidopsis RAC1 and RPP13 disease resistance genes
Source: PLoS Genet. 2018 Dec 13;14(12):e1007843. doi: 10.1371/journal.pgen.1007843 (PMC6307820; doi:10.1371/journal.pgen.1007843)
Supplement: S17 Table — The ‘Total’ column lists the numbers of read pairs obtained. The number of read pairs surviving sequential analysis filters are listed in order to identify RAC1 crossover read pairs. Paired end reads (end1 and end2) were separated and aligned to the Col or Ler RAC1 parental template sequences, allowing only exact matches (Mapped). Read pair ends that mapped uniquely to either Col or Ler were kept (Unique). Read pair ends (1 and 2) that mapped to Col and Ler were identified (Matched), where the Ler mapping read had a lower coordinate than the Col mapping read (Orientate), and that were on opposite strands (Strand). Table (A) shows the reads obtained from libraries generated from ~300 crossovers from wild type, fancm, recq4a recq4b and fancm recq4a recq4b, while Table (B) shows those obtained from ~1,000 crossover libraries for the same genotypes. (DOCX) [file pgen.1007843.s022.docx]

**S17 Table. Mapping reads and filtering during *RAC1* pollen-seq in wild type and *fancm*, *recq4a recq4b* and *fancm recq4a recq4b* mutants.**

(A) ~300 crossover experiment

| **Wild type** |  |  |  |  |  |  |  |
| --- | --- | --- | --- | --- | --- | --- | --- |
| Total | name | Mapped | Unique | Match | Orientate | Strand | Final |
| 18,536,320 | end1-col | 9,288,238 | 5,664,209 | 93,933 | 83,972 | 83,911 | 154,702 |
| 18,536,320 | end2-ler | 7,858,999 | 4,809,390 |  |  |  |  |
| 18,536,320 | end1-ler | 8,320,145 | 4,696,116 | 81,214 | 70,826 | 70,791 |  |
| 18,536,320 | end2-col | 8,563,629 | 5,514,020 |  |  |  |  |
| ***recq4a recq4b*** |  |  |  |  |  |  |  |
| Total | name | Mapped | Unique | Match | Orientate | Strand | Final |
| 18,907,512 | end1-col | 9,501,612 | 5,954,578 | 96,651 | 86,784 | 86,683 | 157,621 |
| 18,907,512 | end2-ler | 7,602,955 | 4,569,987 |  |  |  |  |
| 18,907,512 | end1-ler | 8,068,192 | 4,521,158 | 80,959 | 71,023 | 70,938 |  |
| 18,907,512 | end2-col | 8,802,280 | 5,769,312 |  |  |  |  |
| ***fancm*** |  |  |  |  |  |  |  |
| Total | name | Mapped | Unique | Match | Orientate | Strand | Final |
| 22,724,732 | end1-col | 11,590,914 | 7,497,405 | 119,597 | 105,734 | 105,610 | 201,416 |
| 22,724,732 | end2-ler | 8,735,356 | 5,203,926 |  |  |  |  |
| 22,724,732 | end1-ler | 9,320,743 | 5,227,234 | 110,107 | 95,895 | 95,806 |  |
| 22,724,732 | end2-col | 10,776,827 | 7,245,397 |  |  |  |  |
| ***recq4a recq4b fancm*** |  |  |  |  |  |  |  |
| Total | name | Mapped | Unique | Match | Orientate | Strand | Final |
| 19,112,646 | end1-col | 9,302,147 | 5,785,295 | 92,393 | 83,146 | 83,079 | 156,609 |
| 19,112,646 | end2-ler | 7,978,908 | 4,934,895 |  |  |  |  |
| 19,112,646 | end1-ler | 8,443,895 | 4,927,043 | 83,160 | 73,593 | 73,530 |  |
| 19,112,646 | end2-col | 8,654,592 | 5,610,579 |  |  |  |  |

(B) ~1,000 crossover experiment

| **Wild type** |  |  |  |  |  |  |  |
| --- | --- | --- | --- | --- | --- | --- | --- |
| Total | name | Mapped | Unique | Match | Orientate | Strand | Final |
| 21,517,099 | end1-col | 10,504,979 | 6,567,827 | 101,999 | 91,729 | 91,645 | 181,507 |
| 21,517,099 | end2-ler | 9,598,462 | 6,044,095 |  |  |  |  |
| 21,517,099 | end1-ler | 10,058,451 | 6,121,299 | 100,491 | 89,951 | 89,862 |  |
| 21,517,099 | end2-col | 9,942,487 | 6,388120 |  |  |  |  |
| ***recq4a recq4b*** |  |  |  |  |  |  |  |
| Total | name | Mapped | Unique | Match | Orientate | Strand | Final |
| 20,762,371 | end1-col | 10,423,817 | 6,782,611 | 105,223 | 94,255 | 94,171 | 181,378 |
| 20,762,371 | end2-ler | 8,165,161 | 5,026,486 |  |  |  |  |
| 20,762,371 | end1-ler | 8,886,659 | 5,245,453 | 98,203 | 87,283 | 87,207 |  |
| 20,762,371 | end2-col | 9,590,156 | 6,451,481 |  |  |  |  |
| ***fancm*** |  |  |  |  |  |  |  |
| Total | name | Mapped | Unique | Match | Orientate | Strand | Final |
| 22,254,557 | end1-col | 11,303,163 | 7,335,433 | 117,136 | 104,444 | 104,370 | 203,645 |
| 22,254,557 | end2-ler | 8,984,683 | 5,508,776 |  |  |  |  |
| 22,254,557 | end1-ler | 9,615,108 | 5,647,378 | 112,102 | 99,347 | 99,275 |  |
| 22,254,557 | end2-col | 10,556,712 | 7,080,805 |  |  |  |  |
| ***recq4a recq4b fancm*** |  |  |  |  |  |  |  |
| Total | name | Mapped | Unique | Match | Orientate | Strand | Final |
| 22,148,284 | end1-col | 11,169,218 | 7,097,717 | 112,522 | 102,087 | 101,962 | 196,310 |
| 22,148,284 | end2-ler | 9,406,104 | 5,818,534 |  |  |  |  |
| 22,148,284 | end1-ler | 9,991,957 | 5,919,456 | 105,199 | 94,455 | 94,348 |  |
| 22,148,284 | end2-col | 10,453,400 | 6,865,830 |  |  |  |  |
