# Supplementary material for: Interhomolog polymorphism shapes meiotic crossover within the Arabidopsis RAC1 and RPP13 disease resistance genes
Source: PLoS Genet. 2018 Dec 13;14(12):e1007843. doi: 10.1371/journal.pgen.1007843 (PMC6307820; doi:10.1371/journal.pgen.1007843)
Supplement: S18 Table — Using adjacent windows of the indicated size, correlations (Spearman’s) were performed against crossover reads pairs and polymorphism density calculated against the Col×Ler panmolecule. P values are printed below the correlation coefficient in parentheses. (DOCX) [file pgen.1007843.s023.docx]

**S18 Table. Correlation between polymorphisms and crossover reads in pollen-sequencing data.**

| Genotype | Window size (bp) | | |  | | |
| --- | --- | --- | --- | --- | --- | --- |
|  | 50 bp | 100 bp | 150 bp | 200 bp | 250 bp | 300 bp |
| Wild type | -0.399  (1.11×10^-8^) | -0.497  (2.99×10^-7^) | -0.584  (5.08×10^-7^) | -0.614  (4.35×10^-6^) | -0.636  (1.78×10^-5^) | -0.655  (6.38×10^-5^) |
| *fancm* | -0.338  (1.67×10^-6^) | -0.429  (1.44×10^-5^) | -0.448  (2.30×10^-4^) | -0.526  (1.50×10^-4^) | -0.580  (1.40×10^-4^) | -0.601  (3.50×10^-4^) |
| *recq4a recq4b* | -0.353  (5.65×10^-7^) | -0.437  (9.65×10^-6^) | -0.507  (2.25×10^-5^) | -0.541  (8.70×10^-5^) | -0.567  (2.0×10^-4^) | -0.589  (4.9×10^-4^) |
| *recq4a recq4b fancm* | -0.364  (2.34×10^-7^) | -0.450  (4.76×10^-6^) | -0.480  (6.87×10^-5^) | -0.549  (6.47×10^-5^) | -0.568  (2.00×10^-4^) | -0.616  (2.20×10^-4^) |
