# Supplementary material for: Interhomolog polymorphism shapes meiotic crossover within the Arabidopsis RAC1 and RPP13 disease resistance genes
Source: PLoS Genet. 2018 Dec 13;14(12):e1007843. doi: 10.1371/journal.pgen.1007843 (PMC6307820; doi:10.1371/journal.pgen.1007843)
Supplement: S19 Table — Red highlighting indicates the position of a Col/Ler SNP. If the oligo sequence is entirely red, it hybridizes to a sequence only present in one accession (indel). (DOCX) [file pgen.1007843.s024.docx]

**S19 Table. Pollen-typing allele specific primers for *RAC1* and *RPP13*.**

| Primer name | Sequence (5’-3’) |
| --- | --- |
| RAC1 Ler F 1^st^ | CTGACTTGAGTGATCGCAA |
| RAC1 Col F 1^st^ | AAAACGTGCAACCTAAGAAC |
| RAC1 Col R 1^st^ | ATTTCACCCGATGTAGTCC |
| RAC1 Ler F 2^nd^ | GTGGCCGCAAGCAAAAATAT |
| RAC1 Col F 2^nd^ | AACAGATTGGTCTCATTG |
| RAC1 Col R 2^nd^ | TAGTTTTTCTGACCCCAC |
| RPP13 Col F 1^st^ | GCT TGG ACC TCG ATT TC |
| RPP13 Ler R 1^st^ | CCG ACG GTC CAG ATC TG |
| RPP13 Col R 1^st^ | CAC CGA CGG TCC AGA TCG C |
| RPP13 Col F 2^nd^ | GGA CCT CGA TTT CAT TTA A |
| RPP13 Ler R 2^nd^ | GAT TCA ATA GCA GAT CG |
| RPP13 UR 2^nd^ | GTA AAA CGA TCA ACG ATC ATT C |
