# Supplementary material for: Interhomolog polymorphism shapes meiotic crossover within the Arabidopsis RAC1 and RPP13 disease resistance genes
Source: PLoS Genet. 2018 Dec 13;14(12):e1007843. doi: 10.1371/journal.pgen.1007843 (PMC6307820; doi:10.1371/journal.pgen.1007843)
Supplement: S20 Table — Primer combinations are listed for use in RAC1 and RPP13 pollen typing amplifications, together with the PCR parameters used. (DOCX) [file pgen.1007843.s025.docx]

**S20 Table. *RAC1* and *RPP13* pollen typing PCR parameters.**

| Hotspot | Target | First PCR  primers | Annealing temperature | Cycle number | Second PCR  primers | Annealing temperature | Cycle number |
| --- | --- | --- | --- | --- | --- | --- | --- |
| *RAC1* | Parental | RAC1 Col F 1^st^  RAC1 Col R 1^st^ | 60^o^C to 58 ^o^C (-0.4^o^C/cycle) | 32 | RAC1 Col F 2^nd^  RAC1 Col R 1^st^ | 60^o^C to 58 ^o^C (-0.4^o^C/cycle) | 32 |
| *RAC1* | Crossover | RAC1 Ler F 1^st^  RAC1 Col R 1^st^ | 65^o^C to 63 ^o^C (-0.4^o^C/cycle) | 32 | RAC1 Ler F 2^nd^  RAC1 Col F 2^nd^ | 65^o^C to 63 ^o^C (-0.4^o^C/cycle) | 33 |
| *RPP13* | Parental | RPP13 Col F 1^st^  RPP13 Col R 1^st^ | 62^o^C to 58 ^o^C (-0.5^o^C/cycle) | 28 | RPP13 Col F 2^nd^  RPP13 UR 2^nd^ | 62^o^C to 58 ^o^C (-0.5^o^C/cycle) | 30 |
| *RPP13* | Crossover | RPP13 Col F 1^st^  RPP13 Ler R 1^st^ | 62^o^C to 58 ^o^C (-0.5^o^C/cycle) | 28 | RPP13 Col F 2^nd^  RPP13 Ler R 2^nd^ | 62^o^C to 58 ^o^C (-0.5^o^C/cycle) | 30 |
